# Supplementary material for: PICDGI: A framework for predicting cancer driver genes through dynamic gene-gene interaction modeling of single-cell data
Source: PLoS Comput Biol. 2026 Apr 27;22(4):e1014143. doi: 10.1371/journal.pcbi.1014143 (PMC13119913; doi:10.1371/journal.pcbi.1014143)
Supplement: S1 Text — (DOCX) [file pcbi.1014143.s001.docx]

**S1 Text. Parameters of ARMA model**

The parameters $\boldsymbol{\phi}$**,** and $\boldsymbol{\Lambda}$ correspond to the matrix notation of the ARMA time-varying autoregressive and moving average coefficients in lag-polynomial form.

$$\boldsymbol{\phi}_{k}\boldsymbol{=}\left( \begin{matrix} 1 & {-\phi}_{k,1} & \ldots& {-\phi}_{k,m} & \cdots& 0 \\ 0 & 1 & {-\phi}_{k,1} & \cdots& {-\phi}_{k,m} & {-\phi}_{k,m+1} \\ 0 & 0 & \ddots& \ddots& \vdots& \vdots\\ \vdots& \ddots& \vdots& 1 & {-\phi}_{k,1} & {-\phi}_{k,2} \\ 0 & \vdots& 0 & 0 & 1 & {-\phi}_{k,1} \\ 0 & 0 & \cdots& 0 & 0 & 1 \end{matrix} \right)$$

$$\boldsymbol{\Lambda}_{k}\boldsymbol{=}\left( \begin{matrix} 1 & \lambda_{k,1} & \ldots& \lambda_{k,n} & \ldots& 0 \\ 0 & 1 & \lambda_{k,1} & \ldots& \lambda_{k,n} & \lambda_{k,n+1} \\ 0 & 0 & \ddots& \ddots& \vdots& \vdots\\ \vdots& \ddots& \vdots& 1 & \lambda_{k,1} & \lambda_{k,2} \\ 0 & \vdots& 0 & 0 & 1 & \lambda_{k,1} \\ 0 & 0 & \cdots& 0 & 0 & 1 \end{matrix} \right)$$
