# Supplementary material for: PICDGI: A framework for predicting cancer driver genes through dynamic gene-gene interaction modeling of single-cell data
Source: PLoS Comput Biol. 2026 Apr 27;22(4):e1014143. doi: 10.1371/journal.pcbi.1014143 (PMC13119913; doi:10.1371/journal.pcbi.1014143)
Supplement: S2 Text — (DOCX) [file pcbi.1014143.s002.docx]

**S2 Text. Optimization of the Hurst Parameter** $\boldsymbol{H}$ **in Covariance Computation**

For sequence lengths $k=\left\{ 1, 2, 3,\cdots\right\}$ corresponding to the pseudo time-series data, we compute the covariance matrix $\boldsymbol{C}_{\boldsymbol{u}_{\boldsymbol{k}}}$ for the zero-mean Gaussian vector $\boldsymbol{u}_{k}$ as:

$$\boldsymbol{C}_{\boldsymbol{u}_{\boldsymbol{k}}}=\sigma_{u}^{2}\boldsymbol{R}_{\boldsymbol{u}_{\boldsymbol{k}}}$$

where the correlation matrix $\boldsymbol{R}_{\boldsymbol{u}_{\boldsymbol{k}}}$ is defined as:

$$\boldsymbol{R}_{\boldsymbol{u}_{\boldsymbol{k}}}\boldsymbol{=}\left( \begin{matrix} \rho_{u}(0) & \rho_{u}(1) & \cdots& \rho_{u}(K-1) \\ \rho_{u}(1) & \rho_{u}(0) & \cdots& \rho_{u}(K-2) \\ \vdots& \vdots& \ddots& \vdots\\ \rho_{u}(K-2) & \rho_{u}(K-1) & \cdots& \rho_{u}(1) \\ \rho_{u}(K-1) & \rho_{u}(K-2) & \cdots& \rho_{u}(0) \end{matrix} \right)$$

With

$$\rho_{u}\left( \tau\right)=E\left[ u_{k+\tau}u_{k} \right]=\frac{1}{2}\left[ \left| \tau+1 \right|^{2H}-{2\left| \tau\right|}^{2H}+\left| \tau-1 \right|^{2H} \right]$$

We observe that the covariance matrix structure changes with different values of $H$, affecting how long-range dependencies are captured. A low $H$ underestimates these dependencies, while a high $H$ overestimates them. Our goal is to determine the optimal $H$ that minimizes the error, ensuring the best match to the observed covariance structure.

Since the variance $\sigma_{u}$ is independent on the Hurst exponent, we optimize $H$ by minimizing the difference between the estimated covariance matrix $C(H)$ (computed for a given $H$) and the observed covariance matrix $C^{obs}$(derived from real or simulated data). Here, the normalized covariance structure is given by the correlation matrix $\boldsymbol{R}_{\boldsymbol{u}_{\boldsymbol{k}}}$, such that $C(H) ={\sigma_{u}^{\boldsymbol{2}}\boldsymbol{R}}_{\boldsymbol{u}_{\boldsymbol{k}}}$**.**We formulate the optimization problem as:

$$H^{*}=arg \min_{H} \mathcal{L(}H)$$

Where the loss function $\mathcal{L(}H)$ is:

$$\mathcal{L(}H)=\sum_{i=1}^{K} \sum_{j=1}^{K} \left( C_{ij}\left( H \right)-C_{ij}^{obs} \right)^{2}$$

Thus, we obtain the optimal $H$ by:

$$H^{*}=arg \min_{H} \sum_{i=1}^{K} \sum_{j=1}^{K} \left( C_{ij}\left( H \right)-C_{ij}^{obs} \right)^{2}$$

By minimizing the sum of squared differences, we identify the best $H$ that accurately reflects the observed correlation structure.
