# Supplementary material for: PICDGI: A framework for predicting cancer driver genes through dynamic gene-gene interaction modeling of single-cell data
Source: PLoS Comput Biol. 2026 Apr 27;22(4):e1014143. doi: 10.1371/journal.pcbi.1014143 (PMC13119913; doi:10.1371/journal.pcbi.1014143)
Supplement: S3 Text — (DOCX) [file pcbi.1014143.s003.docx]

**S3 Text. Variational Bayesian inference**

We employ **Variational Bayesian inference** as a powerful framework for approximating complex posterior distributions in probabilistic models. Our primary objective is to transform the challenge of computing an intractable posterior distribution into an optimization problem, making inference computationally more feasible. This approach is particularly advantageous for models with hidden variables, where exact inference is impractical due to high-dimensional integrations.

Our variational inference framework begins by defining a joint probability density function over the observed variables $\boldsymbol{z}_{k}$ and the hidden (local and global) random variables $\boldsymbol{h}_{k}=\left\{ \boldsymbol{x}_{k},\boldsymbol{\varphi} \right\}$:

$$p\left( \boldsymbol{z}_{k},\boldsymbol{h}_{k} \right)=p\left( \boldsymbol{h}_{k} \right)p\left( \boldsymbol{z}_{k}| \boldsymbol{h}_{k} \right) (1)$$

where $p\left( \boldsymbol{h}_{k} \right)$ represents the prior distribution over the hidden variables, and $p\left( \boldsymbol{z}_{k}|\boldsymbol{h}_{k} \right)$denotes the likelihood function. Our goal is to approximate the posterior distribution $p\left( h/z \right)$ and compute the marginal likelihood for model comparison.

Given an observed data point $\boldsymbol{z}_{k}$, our primary objectives of variational inference are:

- Estimating the posterior distribution $p\left( \boldsymbol{h}_{k}|\boldsymbol{z}_{k} \right)$, which characterizes the conditional density of $\boldsymbol{h}$ given $\boldsymbol{z}$**.**
- Computing the marginal likelihood (also known as the model evidence) using:

$$ln p\left( \boldsymbol{z}_{k} \right)=\ln\int_{\boldsymbol{h}} p\left( \boldsymbol{z}_{k},\boldsymbol{h}_{k} \right)d\boldsymbol{h}. (2)$$

The log model evidence serves as a basis for comparing different models based on their ability to explain the observed data $\boldsymbol{z}_{k}$. However, since our model involves multiple hidden random variables, such as local and global variables, it leads to computational intractability. Therefore, we employ variational inference to bypass direct integration by approximating the log model evidence using a lower bound. This reformulation transforms the inference problem into an optimization problem by decomposing the log model evidence as follows:

$$ln p\left( \boldsymbol{z}_{k} \right)=F\left[ q(\boldsymbol{h}_{k}) \right]+KL\left[ \left. q\left( \boldsymbol{h}_{k} \right) \right\|p\left( \boldsymbol{h}_{k}|\boldsymbol{z}_{k} \right) \right] (3)$$

where $q\left( \boldsymbol{h}_{k} \right)$ is a variational distribution that approximates the intractable posterior $p\left( {\boldsymbol{h}_{k}}/{\boldsymbol{z}_{k}} \right)$. Since $\boldsymbol{h}_{k}$includes both local and global hidden variables, the decomposition in Equation (3) consists of two key information-theoretic terms:

- **Variational Free Energy (ELBO)**, defined as:

$$F\left[ q(\boldsymbol{h}_{k}) \right]=\int_{\boldsymbol{h}} q(\boldsymbol{h}_{k})\ln\frac{p\left( \boldsymbol{z}_{k},\boldsymbol{h}_{k} \right)}{q\left( \boldsymbol{h}_{k} \right)}d\boldsymbol{h} (4)$$

- **Kullback-Leibler (KL) Divergence**, which quantifies the divergence between the variational distribution $q(\boldsymbol{h}_{k})$ and the true posterior $p\left( \boldsymbol{h}_{k}|\boldsymbol{z}_{k} \right)$:

$$KL\left[ \left. q\left( \boldsymbol{h}_{k} \right) \right\|p\left( {\boldsymbol{h}_{k}}/{\boldsymbol{z}_{k}} \right) \right] =\int_{\boldsymbol{h}} q(\boldsymbol{h}_{k})\ln\frac{q\left( \boldsymbol{h}_{k} \right)}{p\left( \boldsymbol{h}_{k}|\boldsymbol{z}_{k} \right)}d\boldsymbol{h} (5)$$

By maximizing the variational free energy, we minimize the KL divergence between $q(\boldsymbol{h}_{k})$ and the true posterior $p\left( {\boldsymbol{h}_{k}}/{\boldsymbol{z}_{k}} \right)$, thereby achieving a more accurate approximation of the log model evidence. Since we can treat local and global hidden variables interchangeably, we rewrite Equation (4) as:

$$F\left[ q(\boldsymbol{x}_{k},\boldsymbol{\varphi}) \right]=\iint q(\boldsymbol{x}_{k},\boldsymbol{\varphi})\ln\frac{p\left( \boldsymbol{z}_{k},\boldsymbol{x}_{k},\boldsymbol{\varphi} \right)}{q\left( \boldsymbol{x}_{k},\boldsymbol{\varphi} \right)}d\boldsymbol{x}d\boldsymbol{\varphi} (6)$$

A key challenge we face in variational inference is striking a balance between the expressiveness of $q\left( \boldsymbol{x}_{k},\boldsymbol{\varphi} \right)$ to closely approximate the true posterior and ensuring computational tractability. To address this, we adopt the mean-field approximation, which assumes a fully factorized distribution over the hidden variables.

***Mean field approximation of the variational free energy***

Here, we provide a detailed explanation of the **mean field approximation** of the variational free energy, which we utilize to construct a simple bound for the **KL divergence[1, 2].** The mean field approximation assumes that the latent (hidden) variables are independent, simplifying the computational complexity of our inference problem. Specifically, we express this assumption as $q\left( \boldsymbol{x}_{k},\boldsymbol{\varphi} \right)\boldsymbol{=}q\left( \boldsymbol{x}_{k} \right)q\left( \boldsymbol{\varphi} \right)$, where $\boldsymbol{x}_{k}$​ and $\boldsymbol{\varphi}$ are local and global hidden variables, respectively. Our approach to applying the Mean Field approximation involves the following seven steps:

**Step 1**: Simplifying the Variational Free Energy

### We begin by simplifying the variational free energy, as expressed in Equation (6), under the assumption of independent latent variables:

$$F\left[ q\left( \boldsymbol{x}_{k} \right),q(\boldsymbol{\varphi}) \right]=\iint q\left( \boldsymbol{x}_{k} \right)q\left( \boldsymbol{\varphi} \right)ln\left[ \frac{p\left( \boldsymbol{z}_{k},\boldsymbol{x}_{k},\boldsymbol{\varphi} \right)}{q\left( \boldsymbol{x}_{k} \right)q\left( \boldsymbol{\varphi} \right)} \right]d\boldsymbol{x}d\boldsymbol{\varphi.} (7)$$

This equation involves the joint probability distribution of the observed data $\boldsymbol{z}_{k}$ and the hidden variables $\boldsymbol{x}_{k}$ and $\boldsymbol{\varphi}$. We apply the **quotient rule for logarithms** to break down the logarithmic term into two separate components:

$$F\left[ q\left( \boldsymbol{x}_{k} \right),q(\boldsymbol{\varphi}) \right]=\iint q\left( \boldsymbol{x}_{k} \right)q\left( \boldsymbol{\varphi} \right)[\ln p\left( \boldsymbol{z}_{k},\boldsymbol{x}_{k},\boldsymbol{\varphi} \right) -\ln q\left( \boldsymbol{x}_{k} \right)-\ln q\left( \boldsymbol{\varphi} \right)]d\boldsymbol{x}d\boldsymbol{\varphi} (8)$$

This simplification separates the terms involving the **likelihood** $\ln p\left( \boldsymbol{z}_{k},\boldsymbol{x}_{k},\boldsymbol{\varphi} \right)$ and the **variational distributions** $\ln q\left( \boldsymbol{x}_{k} \right)$and $\ln q\left( \boldsymbol{\varphi} \right)$

**Step 2:** Reformulation with Respect to $\boldsymbol{x}_{k}$

Next, we focus on the **local hidden variable** $\boldsymbol{x}_{k}$. The expression can be restructured as follows:

$$F\left[ q\left( \boldsymbol{x}_{k} \right),q(\boldsymbol{\varphi}) \right]=\iint q\left( \boldsymbol{x}_{k} \right)q\left( \boldsymbol{\varphi} \right)\ln\left( p\left( \boldsymbol{z}_{k},\boldsymbol{x}_{k},\boldsymbol{\varphi} \right)-\ln q(\boldsymbol{x}_{k}) \right)d\boldsymbol{x}d\boldsymbol{\varphi} -\int q\left( \boldsymbol{\varphi} \right)\ln q\left( \boldsymbol{\varphi} \right)\left[ \int q\left( \boldsymbol{x}_{k} \right)d\boldsymbol{x} \right]d\boldsymbol{\varphi} (9)$$

Since the integral of a probability density function $q\left( \boldsymbol{x}_{k} \right)$ is 1, the second term simplifies to: $\int q\left( \boldsymbol{\varphi} \right)\ln q\left( \boldsymbol{\varphi} \right)d\boldsymbol{\varphi}$.

This allows us to further simplify the variational free energy to:

$$F\left[ q\left( \boldsymbol{x}_{k} \right),q\left( \boldsymbol{\varphi} \right) \right]=\int q\left( \boldsymbol{x}_{k} \right)\left[ \int q\left( \boldsymbol{\varphi} \right)\ln p\left( \boldsymbol{z}_{k},\boldsymbol{x}_{k},\boldsymbol{\varphi} \right)d\boldsymbol{\varphi} \right]d\boldsymbol{x} -\int q\left( \boldsymbol{x}_{k} \right)\ln q\left( \boldsymbol{x}_{k} \right)d\boldsymbol{x-}\int q\left( \boldsymbol{\varphi} \right)\ln q\left( \boldsymbol{\varphi} \right)d\boldsymbol{\varphi} (10)$$

### **Step 3**: Simplifying the Free Energy Expression

### Now, we apply the fact that the integral of $\boldsymbol{q}\left( \boldsymbol{\varphi} \right)$ is 1, which simplifies the free energy expression further:

$$F\left[ q\left( \boldsymbol{x}_{k} \right),q\left( \boldsymbol{\varphi} \right) \right]=\int q\left( \boldsymbol{x}_{k} \right)\left[ \int q\left( \boldsymbol{\varphi} \right)\ln p\left( \boldsymbol{z}_{k},\boldsymbol{x}_{k},\boldsymbol{\varphi} \right)d\boldsymbol{\varphi} \right]d\boldsymbol{x} -\int q\left( \boldsymbol{x}_{k} \right)\ln q\left( \boldsymbol{x}_{k} \right)d\boldsymbol{x -c} \left( 11 \right)$$

Where $\boldsymbol{c}$ is a constant w.r.t. $q\left( \boldsymbol{x}_{k} \right)$**.** This equation represents the variational free energy as a combination of two terms: (1) The **expected log likelihood** of the data given the latent variables, and (2) The **entropy term** for the variational distribution over the local hidden variables.

### Step 4: Logarithmic Transformation of the Likelihood

### We continue simplifying the variational free energy by applying a logarithmic transformation to the likelihood term. This results in:

$$F\left[ q\left( \boldsymbol{x}_{k} \right),q\left( \boldsymbol{\varphi} \right) \right]=\int q\left( \boldsymbol{x}_{k} \right)\left[ \ln\left( \exp\int q\left( \boldsymbol{\varphi} \right)\ln p\left( \boldsymbol{z}_{k},\boldsymbol{x}_{k},\boldsymbol{\varphi} \right)d\boldsymbol{\varphi} \right) \right]d\boldsymbol{x} -\int q\left( \boldsymbol{x}_{k} \right)\ln q\left( \boldsymbol{x}_{k} \right)d\boldsymbol{x -c} (12)$$

This step effectively brings the expression into a form that is easier to optimize, as we now have an expectation of the logarithmic likelihood of the data over the variational distribution.

### Step 5: Final Expression and Kullback-Leibler Divergence

### At this point, we rewrite the variational free energy in terms of the **KL divergence** between the variational distribution and the posterior distribution:

$$F\left[ q\left( \boldsymbol{x}_{k} \right),q(\boldsymbol{\varphi}) \right]=\int q\left( \boldsymbol{x}_{k} \right)\left[ \ln\left( \frac{\exp\int q\left( \boldsymbol{\varphi} \right)\ln p\left( \boldsymbol{z}_{k},\boldsymbol{x}_{k},\boldsymbol{\varphi} \right)d\boldsymbol{\varphi}}{q\left( \boldsymbol{x}_{k} \right)} \right) \right]d\boldsymbol{x} \boldsymbol{-c (}13\boldsymbol{)}$$

This negative KL divergence represents the difference between the variational approximation and the true posterior. We now aim to **minimize** this KL divergence, which is equivalent to **maximizing** the variational free energy.

### Step 6: Maximizing the Variational Free Energy

### To maximize the variational free energy, we set:

$q\left( \boldsymbol{x}_{k} \right)\propto exp(\mathbb{E}_{q\left( \boldsymbol{\varphi} \right)}\boldsymbol{])}\boldsymbol{(}14\boldsymbol{)}$

This leads to the following equation:

$$\ln q\left( \boldsymbol{x}_{k} \right)=\int d\boldsymbol{\varphi}q\left( \boldsymbol{\varphi} \right)\ln p\left( \boldsymbol{z}_{k},\boldsymbol{x}_{k},\boldsymbol{\varphi} \right) (15)$$

This equation indicates that the variational distribution $q\left( \boldsymbol{x}_{k} \right)$ is determined by the **expected log likelihood** of the data over the global hidden variables $\boldsymbol{\varphi}$.

### Step 7: Joint Probability and Mean-Field Approximation

### Finally, we express the joint probability $\boldsymbol{p}\left( \boldsymbol{z}_{\boldsymbol{k}}\boldsymbol{,}\boldsymbol{x}_{\boldsymbol{k}}\boldsymbol{,\varphi} \right)$ as the product of the likelihood $\boldsymbol{p}\left( \boldsymbol{z}_{\boldsymbol{k}}\boldsymbol{|}\boldsymbol{x}_{\boldsymbol{k}}\boldsymbol{,\varphi} \right)$ and the joint posterior distribution for the global and local hidden variables:

$$p\left( \boldsymbol{z}_{k},\boldsymbol{x}_{k},\boldsymbol{\varphi} \right)=p\left( \boldsymbol{z}_{k}|\boldsymbol{x}_{k},\boldsymbol{\varphi} \right)p\left( \boldsymbol{x}_{k},\boldsymbol{\varphi} \right) (16)$$

Using the **mean-field approximation** $p\left( \boldsymbol{x}_{k},\boldsymbol{\varphi|}\boldsymbol{z}_{k} \right)\approx q\left( \boldsymbol{x}_{k} \right)q\left( \boldsymbol{\varphi} \right)$[3], we can transform this expression into:

$$p\left( \boldsymbol{z}_{k},\boldsymbol{x}_{k},\boldsymbol{\varphi} \right)=q\left( \boldsymbol{x}_{k} \right)q\left( \boldsymbol{\varphi} \right)p\left( \boldsymbol{z}_{k}|\boldsymbol{x}_{k}\boldsymbol{,\varphi} \right) (17)$$

Finally, we can rewrite the Equation 15 for $\ln q\left( \boldsymbol{x}_{k} \right)$ as:

$$\ln q\left( \boldsymbol{x}_{k}|\boldsymbol{\varphi} \right)=\int d\boldsymbol{\varphi}q\left( \boldsymbol{\varphi} \right)\ln\left[ q\left( \boldsymbol{x}_{k} \right)q\left( \boldsymbol{\varphi} \right)\prod_{k=1}^{N} p\left( z_{k}|\boldsymbol{x}_{k}\boldsymbol{,\varphi} \right) \right] (18)$$

This final expression provides a tractable form for the distribution of the dynamics of the local hidden variable (such as gene mutation) in terms of the global hidden variable (gene-gene interaction) and the observed data (mutation sequences).

### *Probability Density Function of the Dynamics of Gene Mutation*

### We follow Thurley et al.’s study [4] regarding the response time distributions of cell-to-cell communication networks, which demonstrate that the interaction between cells or genes can be described by gamma distributions. The gamma distribution is an asymmetric (right-skewed) distribution with a single peak at $\boldsymbol{k>0}$. Based on this, we model the global hidden variables $\boldsymbol{\varphi}$ as gamma-distributed random variables, such that:

$$q\left( \boldsymbol{\varphi} \right)=G\left( \boldsymbol{\varphi};\boldsymbol{a}_{\boldsymbol{\varphi}},\boldsymbol{b}_{\boldsymbol{\varphi}} \right) (19)$$

where the mean $\left\langle\varphi\right\rangle= {a_{\varphi}}/{b_{\varphi}},$ which depends on the shape and scale parameters $a$ and $b$.

We assume the $N$ observations $\boldsymbol{z=}z_{1:N}$ to be normally distributed, with both the mean and variance being random variables of the local and global hidden variables, respectively. This gives the likelihood:

$p\left( \boldsymbol{z}_{1:N}|\boldsymbol{x}_{1:N}\boldsymbol{,\varphi} \right)=\prod_{k} \mathcal{N}\left( z_{k}|x_{k}\boldsymbol{,\varphi} \right) (20)$

where $\mathcal{N}\left( z_{k}|x_{k}\boldsymbol{,}\varphi\right)$ is the normal distribution with the random mean $x_{k}$ and variance dependent on the global hidden variable $\varphi$. Therefore, for $q\left( \boldsymbol{x}_{k} \right)\mathcal{=N}\left( \boldsymbol{x}_{k}{;\mu}_{\boldsymbol{x}_{\boldsymbol{k}}},\boldsymbol{C}_{\boldsymbol{x}_{\boldsymbol{k}}} \right)$, and $q\left( \boldsymbol{\varphi} \right)$ given in Equation 19, the joint probability density function of the hidden variables and observations can be expressed as:

$$p\left( \boldsymbol{z}_{k},\boldsymbol{x}_{k},\boldsymbol{\varphi} \right)=\mathcal{N}\left( \boldsymbol{x}_{k}{;\mu}_{\boldsymbol{x}_{\boldsymbol{k}}},\boldsymbol{C}_{\boldsymbol{x}_{\boldsymbol{k}}} \right)G\left( \boldsymbol{\varphi};\boldsymbol{a}_{\boldsymbol{\varphi}},\boldsymbol{b}_{\boldsymbol{\varphi}} \right)\prod_{k} \mathcal{N}\left( z_{k}|x_{k}\boldsymbol{,\varphi} \right) (21)$$

The non-independent Gaussian-Gamma prior density in this equation belongs to the conjugate-exponential class, allowing us to derive an exact analytical solution for the posterior distribution. Thus, we report Equation (21) into Equation (18) as:

$$\ln q\left( \boldsymbol{x}_{k}|\boldsymbol{\varphi} \right)=\int d\boldsymbol{\varphi} G\left( \boldsymbol{\varphi};\boldsymbol{a}_{\boldsymbol{\varphi}},\boldsymbol{b}_{\boldsymbol{\varphi}} \right)ln\left[ G\left( \boldsymbol{\varphi};\boldsymbol{a}_{\boldsymbol{\varphi}},\boldsymbol{b}_{\boldsymbol{\varphi}} \right)\mathcal{N}\left( \boldsymbol{x}_{k}{;\mu}_{\boldsymbol{x}_{\boldsymbol{k}}},\boldsymbol{C}_{\boldsymbol{x}_{\boldsymbol{k}}} \right)\prod_{k} \mathcal{N}\left( z_{k}|x_{k}\boldsymbol{,\varphi} \right) \right] (22)$$

We simplify this expression into the following form:

$$\ln q\left( \boldsymbol{x}_{k}|\boldsymbol{\varphi} \right)=\int d\boldsymbol{\varphi}G\left( \boldsymbol{\varphi};\boldsymbol{a}_{\boldsymbol{\varphi}},\boldsymbol{b}_{\boldsymbol{\varphi}} \right)\ln G\left( \boldsymbol{\varphi};\boldsymbol{a}_{\boldsymbol{\varphi}},\boldsymbol{b}_{\boldsymbol{\varphi}} \right)+\int d\boldsymbol{\varphi}G\left( \boldsymbol{\varphi};\boldsymbol{a}_{\boldsymbol{\varphi}},\boldsymbol{b}_{\boldsymbol{\varphi}} \right)\ln\mathcal{N}\left( \boldsymbol{x}_{k}{;\mu}_{\boldsymbol{x}_{\boldsymbol{k}}},\boldsymbol{C}_{\boldsymbol{x}_{\boldsymbol{k}}} \right)+\sum_{k} \int d\boldsymbol{\varphi} G\left( \boldsymbol{\varphi};\boldsymbol{a}_{\boldsymbol{\varphi}},\boldsymbol{b}_{\boldsymbol{\varphi}} \right)\ln\mathcal{N}\left( z_{k}|x_{k}\boldsymbol{,\varphi} \right) (23)$$

We break down the expression for ln $q\left( \boldsymbol{x}_{k} \right)$ into three terms that are computable with respect to the variable of interest $\boldsymbol{x}_{k}$​. The first term, $\int d\varphi G\left( \boldsymbol{\varphi};\boldsymbol{a}_{\boldsymbol{\varphi}},\boldsymbol{b}_{\boldsymbol{\varphi}} \right)\ln G\left( \boldsymbol{\varphi};\boldsymbol{a}_{\boldsymbol{\varphi}},\boldsymbol{b}_{\boldsymbol{\varphi}} \right)$, does not depend on the local random variables or the latent state, so we can discard it and replace it with a normalization term $\mathcal{M}$. The second term, $\int d\boldsymbol{\varphi}G\left( \boldsymbol{\varphi};\boldsymbol{a}_{\boldsymbol{\varphi}},\boldsymbol{b}_{\boldsymbol{\varphi}} \right)\ln\mathcal{N}\left( \boldsymbol{x}_{k}{;\mu}_{\boldsymbol{x}_{\boldsymbol{k}}},\boldsymbol{C}_{\boldsymbol{x}_{\boldsymbol{k}}} \right)$, depends on $\boldsymbol{x}_{k}$ but not on $\boldsymbol{\varphi}$, so it simplifies to $\ln\mathcal{N}\left( \boldsymbol{x}_{k}{;\mu}_{\boldsymbol{x}_{\boldsymbol{k}}},\boldsymbol{C}_{\boldsymbol{x}_{\boldsymbol{k}}} \right)$. Consequently, we simplify Equation (23) as:

$$\ln q\left( \boldsymbol{x}_{k}|\boldsymbol{\varphi} \right)=\ln\mathcal{N}\left( \boldsymbol{x}_{k}{;\mu}_{\boldsymbol{x}_{\boldsymbol{k}}},\boldsymbol{C}_{\boldsymbol{x}_{\boldsymbol{k}}} \right)+\sum_{k} \int d\boldsymbol{\varphi} G\left( \boldsymbol{\varphi};\boldsymbol{a}_{\boldsymbol{\varphi}},\boldsymbol{b}_{\boldsymbol{\varphi}} \right)\ln\mathcal{N}\left( z_{k}|x_{k}\boldsymbol{,\varphi} \right)+\ln\frac{1}{\mathcal{M}} (24)$$

To evaluate the third term, we approximate the log-likelihood component as a quadratic form involving the distance between $z_{k}$ and $x_{k}$:

$${\ln\mathcal{N}\left( {z_{k}}/{\boldsymbol{x}_{k},}\boldsymbol{\varphi} \right)\approx-\frac{1}{2}\left( z_{k}-x_{k} \right)}^{T}\varphi\left( z_{k}-x_{k} \right)\mathcal{+e}$$

where the constant $\mathcal{e}$ ensures proper normalization and may absorb factors unrelated to $x_{k}$ and $\varphi$. The required integral then becomes the expected value of $\varphi$ under its Gamma distribution:

$$\sum_{k} \int d\varphi G\left( \boldsymbol{\varphi};\boldsymbol{a}_{\boldsymbol{\varphi}},\boldsymbol{b}_{\boldsymbol{\varphi}} \right)\ln\mathcal{N}\left( z_{k}|x_{k}\boldsymbol{,\varphi} \right)=-\sum_{k} \left( z_{k}-x_{k} \right)^{T}\left[ \int d\boldsymbol{\varphi} G\left( \boldsymbol{\varphi};\boldsymbol{a}_{\boldsymbol{\varphi}},\boldsymbol{b}_{\boldsymbol{\varphi}} \right)\boldsymbol{\varphi} \right]\left( z_{k}-x_{k} \right) (25)$$

Where we define the expectation:

$$\left\langle\varphi\right\rangle=\int d\boldsymbol{\varphi} G\left( \boldsymbol{\varphi};\boldsymbol{a}_{\boldsymbol{\varphi}},\boldsymbol{b}_{\boldsymbol{\varphi}} \right)\varphi$$

as the expected value of the global random variables. Therefore, substituting back, Equation (25) simplifies to:

$$\ln q\left( \boldsymbol{x}_{k}|\boldsymbol{\varphi} \right)=\ln\mathcal{N}\left( \boldsymbol{x}_{k}{;\mu}_{\boldsymbol{x}_{\boldsymbol{k}}},\boldsymbol{C}_{\boldsymbol{x}_{\boldsymbol{k}}} \right)-\sum_{k} \left( z_{k}-x_{k} \right)^{T}\left\langle\varphi\right\rangle\left( z_{k}-x_{k} \right)+\ln\frac{1}{\mathcal{M}} (26)$$

Exponentiating both sides yields the unnormalized posterior:

$$q\left( \boldsymbol{x}_{k}|\boldsymbol{\varphi} \right)=\frac{1}{\mathcal{M}}\mathcal{N}\left( \boldsymbol{x}_{k}{;\mu}_{\boldsymbol{x}_{\boldsymbol{k}}},\boldsymbol{C}_{\boldsymbol{x}_{\boldsymbol{k}}} \right)\exp\left[ -\sum_{k} \left( z_{k}-x_{k} \right)^{T}\left\langle\boldsymbol{\varphi} \right\rangle\left( z_{k}-x_{k} \right) \right] (27)$$

Here, the expected value of the global hidden variable $\varphi$ is characterized in terms of the shape and scale parameters as $\left\langle\varphi\right\rangle={\boldsymbol{a}_{\boldsymbol{\varphi}}\boldsymbol{b}_{\boldsymbol{\varphi}}}^{-1}$. The covariance $\boldsymbol{C}_{\boldsymbol{x}_{\boldsymbol{k}}}$ included in the approximated distribution $q\left( \boldsymbol{x}_{k} \right)$ provides the Hurst exponent, which is used as a measure of long-term memory in time series analysis. The Equation 27 represents the variational posterior over $x_{k}$, combining prior knowledge with observed data while accounting for uncertainty in the interaction structure through the expected value $\left\langle\varphi\right\rangle$.

**References**

1. Jain V, Koehler F, Mossel E, editors. The mean-field approximation: Information inequalities, algorithms, and complexity. Conference On Learning Theory; 2018: PMLR.

2. Zhu H, Leung H, He Z. A variational Bayesian approach to robust sensor fusion based on Student-t distribution. Information Sciences. 2013;221:201–14.

3. Keng B. Variational Bayes and the mean-field approximation. 2017.

4. Thurley K, Wu LF, Altschuler SJ. Modeling cell-to-cell communication networks using response-time distributions. Cell systems. 2018;6(3):355–67. e5.
