# Supplementary material for: PICDGI: A framework for predicting cancer driver genes through dynamic gene-gene interaction modeling of single-cell data
Source: PLoS Comput Biol. 2026 Apr 27;22(4):e1014143. doi: 10.1371/journal.pcbi.1014143 (PMC13119913; doi:10.1371/journal.pcbi.1014143)
Supplement: S4 Text — (DOCX) [file pcbi.1014143.s004.docx]

**S4 Text. Incorporating Gene-Gene Interactions to Enhance Cancer Driver Gene Prediction**

In this part of our study, we highlight the importance of accounting for gene-gene interactions when modeling gene expression dynamics for the prediction of cancer driver genes (CDGs). Accordingly, we propose a comparative framework involving two models: a **baseline model** that assumes independence among genes, and an **interaction-aware model** that integrates a gene-gene interaction matrix into the posterior distribution. We provide a formal mathematical formulation for both models and demonstrate that incorporating interaction structure significantly improves inference accuracy in the context of CDG prediction.

**Baseline Prediction Model (No Gene-Gene Interactions)**

We consider a simplified version of the variational posterior in Equation 27 of the Supplementary Text 3, $q\left( \boldsymbol{x}_{k}|\boldsymbol{\varphi} \right)$, by omitting the interaction term, to define the baseline probability distribution. In this baseline model, gene expression levels are assumed to be mutually independent, with no consideration of gene-gene interactions. This means that each gene’s expression is drawn independently from a normal distribution, with no influence from other genes. Given observed expression data $\boldsymbol{z}_{k}$, the prediction of gene expression for each gene $\boldsymbol{x}_{k}$ is based on the prior mean $\mu_{\boldsymbol{x}_{\boldsymbol{k}}}$ and covariance $\boldsymbol{C}_{\boldsymbol{x}_{\boldsymbol{k}}}$ as follows:

$$q_{baseline}\left( \boldsymbol{x}_{k} \right)=\frac{1}{\mathcal{M}}\mathcal{N}\left( \boldsymbol{x}_{k}{;\boldsymbol{\mu}}_{\boldsymbol{x}_{\boldsymbol{k}}},\boldsymbol{C}_{\boldsymbol{x}_{\boldsymbol{k}}} \right) (1)$$

Here, $\boldsymbol{\mu}_{\boldsymbol{x}_{\boldsymbol{k}}}$ is the mean vector, and $\boldsymbol{C}_{\boldsymbol{x}_{\boldsymbol{k}}}$ is the covariance matrix governing the system’s dynamics. The normalization constant $\mathcal{M}$ ensures that the probability density function integrates to one. The baseline prediction assumes that gene expressions are independent, so there are no gene-gene dependencies accounted for in the prediction model. The prediction is obtained by computing the **posterior mean** $\hat{x}_{k}^{baseline}$ as:

$$\hat{x}_{k}^{baseline}=\mu_{\boldsymbol{x}_{\boldsymbol{k}}} (2)$$

**Interaction-Aware Prediction (With Gene-Gene Interactions)**

The interaction-aware model, as derived in Equation 27 of the Supplementary Text 3, $q\left( \boldsymbol{x}_{k}|\boldsymbol{\varphi} \right)$, explicitly accounts for gene-gene interactions by introducing a penalty term that incorporates the gene interaction matrix $\left\langle\boldsymbol{\varphi} \right\rangle$. The posterior distribution, considering these interactions, is written as:

$$q_{interact}\left( \boldsymbol{x}_{k}|\boldsymbol{\varphi} \right)=\frac{1}{\mathcal{M}}\mathcal{N}\left( \boldsymbol{x}_{k}{;\mu}_{\boldsymbol{x}_{\boldsymbol{k}}},\boldsymbol{C}_{\boldsymbol{x}_{\boldsymbol{k}}} \right)\exp\left[ -\left( z_{k}-x_{k} \right)^{T}\left\langle\boldsymbol{\varphi} \right\rangle\left( z_{k}-x_{k} \right) \right] (3)$$

In this formulation, the additional exponential term penalizes large deviations between the predicted and observed expression vectors $\boldsymbol{x}_{\boldsymbol{k}}$ and $\boldsymbol{z}_{\boldsymbol{k}}$, where $\left\langle\boldsymbol{\varphi} \right\rangle$ governs how gene interactions affect these deviations.

The **interaction-aware prediction** takes the form of an optimization problem, where the posterior is maximized (or equivalently, the energy function is minimized):

$$\hat{x}_{k}=\underset{x_{k}}{\mathrm{argmin}} \left[ \frac{1}{2}\left( x_{k}-\mu_{\boldsymbol{x}_{\boldsymbol{k}}} \right)^{T}C_{x_{k}}^{-1}\left( x_{k}-\mu_{\boldsymbol{x}_{\boldsymbol{k}}} \right)+\left( z_{k}-x_{k} \right)^{T}\left\langle\boldsymbol{\varphi} \right\rangle\left( z_{k}-x_{k} \right) \right] (4)$$

By computing the gradient of the objective function with respect to $x_{k}$ and setting it to zero, we arrive at the following closed-form expression:

$$C_{x_{k}}^{-1}\left( x_{k}-\mu_{\boldsymbol{x}_{\boldsymbol{k}}} \right)-2\left\langle\boldsymbol{\varphi} \right\rangle\left( z_{k}-x_{k} \right)=0\Longrightarrow\left( C_{x_{k}}^{-1}+2\left\langle\boldsymbol{\varphi} \right\rangle\right)\hat{x}_{k}^{interact}=C_{x_{k}}^{-1}\mu_{\boldsymbol{x}_{\boldsymbol{k}}}+2\left\langle\varphi\right\rangle z_{k} (5)$$

Hence, the interaction-aware prediction $\hat{x}_{k}^{interact}$ is expressed as a weighted combination of the prior mean $\mu_{\boldsymbol{x}_{\boldsymbol{k}}}$ and the observed data $z_{k}$, where the weights are governed by the prior precision $C_{x_{k}}^{-1}$ and the expected gene-gene interaction structure $\left\langle\boldsymbol{\varphi} \right\rangle$:

$$\hat{x}_{k}^{interact}=\left( C_{x_{k}}^{-1}+2\left\langle\boldsymbol{\varphi} \right\rangle\right)^{-1}\left( C_{x_{k}}^{-1}\mu_{\boldsymbol{x}_{\boldsymbol{k}}}+2\left\langle\varphi\right\rangle z_{k} \right) (6)$$

The inclusion of gene interactions results in predictions that are more coherent with biological knowledge, reflecting the dependencies among genes.

**Comparing the Predictions**

With $\mu_{\boldsymbol{x}_{\boldsymbol{k}}}=0$, the key difference between the two models lies in the inclusion of the gene-gene interaction matrix $\left\langle\boldsymbol{\varphi} \right\rangle$ in the interaction-aware model:

- Baseline Model: The posterior mean $\hat{x}_{k}^{baseline}=0$, implying no prior assumption of gene expression level, and the model ignores the observed data and relies solely on the prior for predictions without any consideration of interactions between genes.
- Interaction-Aware Model: The posterior mean $\hat{x}_{k}^{interact}$ is computed considering the gene-gene interaction structure, where the prior is still zero, but the model incorporates the relationships between genes. This typically results in better predictions, as gene interactions can significantly alter the predicted expression dynamics, especially in the context of CDGs.

**Comparison of Posterior Behavior**

We analyze both models through their respective posterior energy functions (negative log posterior densities):

*Baseline energy function:*

$$\zeta_{baseline}\left( x_{k} \right)=\frac{1}{2}{(x_{k}-\mu_{\boldsymbol{x}_{\boldsymbol{k}}})}^{T}C_{x_{k}}^{-1}(x_{k}-\mu_{\boldsymbol{x}_{\boldsymbol{k}}}) (7)$$

*Interaction-aware energy function:*

$$\zeta_{interact}\left( x_{k} \right)=\frac{1}{2}\left( x_{k}-\mu_{\boldsymbol{x}_{\boldsymbol{k}}} \right)^{T}C_{x_{k}}^{-1}\left( x_{k}-\mu_{\boldsymbol{x}_{\boldsymbol{k}}} \right)+\left( z_{k}-x_{k} \right)^{T}\left\langle\boldsymbol{\varphi} \right\rangle\left( z_{k}-x_{k} \right) (8)$$

The baseline model does not account for gene-gene dependencies, while the interaction-aware model incorporates these relationships, improving the prediction by penalizing discrepancies based on the expected interactions between genes.

**Simulation-Driven Assessment**

To quantify the benefit of incorporating $\left\langle\varphi\right\rangle$, we simulate data from a known gene network where the true expression levels $x_{k}^{true}$ are perturbed by Gaussian noise $\epsilon_{k}\mathcal{\sim N}\left( 0,\Sigma\right)$ with $\Sigma=\varphi_{true}^{-1}$. We then fit both models and evaluate them using:

*Posterior Mean Squared Error (MSE):*

$$MSE=\frac{1}{N}\sum_{i=1}^{N} \left( \hat{x}_{k,i}-x_{k,i}^{true} \right)^{2} (9)$$

*Negative Log Posterior (NLP):*

$$NLP=-\log q(x_{k}|z_{k}) (10)$$

### Simulation of Gene Interaction and Expression Dynamics

To model gene interactions and simulate gene expression data, we defined a set of parameters and used a simulation approach based on multivariate normal distributions. The key parameters are:

- **N = 50** represents the number of genes simulated, focusing on 50 genes for which interactions are modeled.
- **K = 3** refers to the number of time points considered.
- **sigma_noise = 0.5** represents the level of noise, a factor that determines the amount of random variation or error added to the observed gene expression data, reflecting the imperfections inherent in experimental measurements.

The simulation centers around generating a **true interaction matrix** that models pairwise gene interactions. This symmetric, positive-definite matrix has diagonal elements set to 1 indicating that each gene is perfectly correlated with itself (perfect self-correlation) and off-diagonal elements randomly sampled from a uniform distribution between -0.3 and 0.3 to represent interaction strength and direction. Symmetry is ensured by mirroring these values, and positive-definiteness is maintained by adding the matrix to its transpose and adjusting the diagonal based on $N$. The **covariance matrix** $\Sigma$ is computed as the inverse of the interaction matrix, capturing the structure of gene co-variation. **True gene expression data** is then sampled from a multivariate normal distribution with mean zero and covariance $\Sigma$, modeling the underlying expression dynamics. To mimic real-world experimental noise, **observed gene expression data** is generated by adding random noise through another multivariate normal distribution, scaled by the noise factor.

In summary, this simulation generates realistic gene expression data for 50 genes, with interactions modeled through a structured matrix and noise introduced to replicate experimental conditions. The resulting dataset supports downstream tasks like gene discovery, network inference, and predictive modeling.

*Simulation Outcome 1: Evaluating the Predictive Accuracy of Gene Expression Models*

The scatter plots in the S1 Fig. visualize the predicted versus true gene expression levels for both the baseline and interaction-aware models. In the baseline model (S1 Fig. A), the predictions show noticeable deviations from the identity line, indicating that the model does not adequately capture the underlying dynamics of gene expression. The spread of data points reflects a lack of correlation between the true and predicted values, underscoring the limitations of assuming independence across genes.


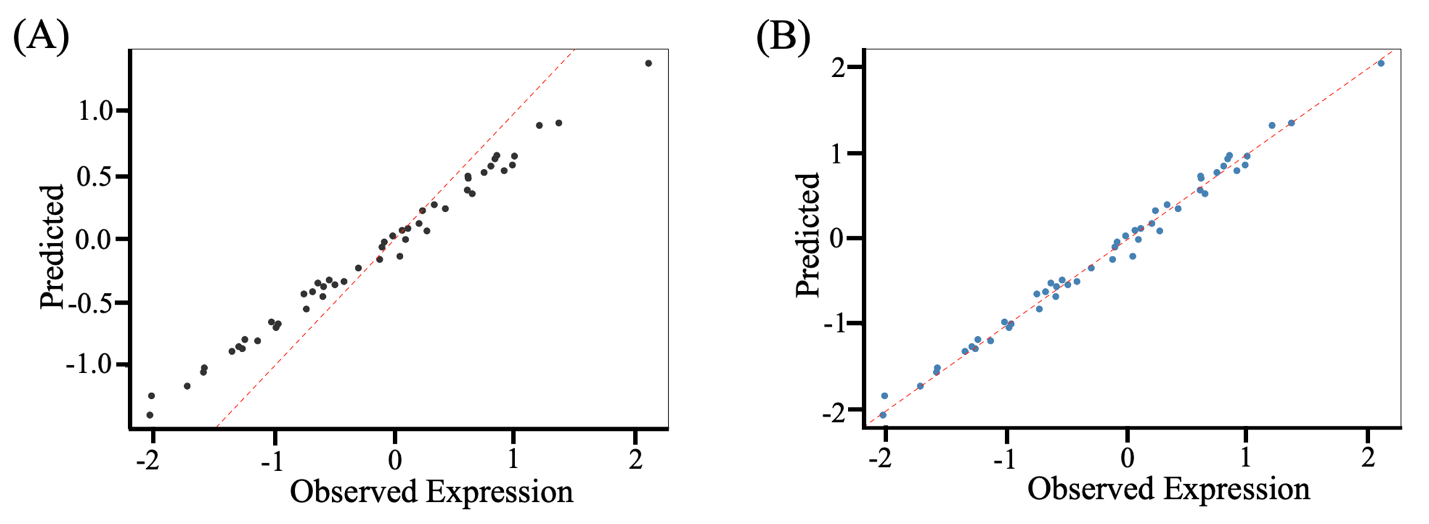


**S1 Fig. Comparison of predicted versus true gene expression levels.** **(A)** Baseline model assuming gene independence; **(B)** Interaction-aware model incorporating gene-gene interactions.

In contrast, the interaction-aware model (S1 Fig. B) exhibits predictions that closely align with the true values. The points are tightly clustered around the diagonal line, reflecting a high degree of accuracy and a strong correspondence between predicted and actual expression levels. This demonstrates the benefit of modeling gene-gene interactions, as the precision matrix encodes structured dependencies that are biologically meaningful and improve the model's expressiveness.

*Simulation Outcome 2: Quantitative Assessment via Mean Squared Error*

We quantified prediction accuracy using the Mean Squared Error (MSE), as shown in the left panel of S2 Fig A. The baseline model yielded a substantially higher MSE, approximately 0.1, while the interaction-aware model achieved an order-of-magnitude reduction in error. This stark contrast illustrates that neglecting gene-gene interactions results in inferior estimates, as the baseline model is unable to leverage co-expression patterns or regulatory relationships among genes. Conversely, the interaction-aware model exploits these dependencies to generate more precise predictions.


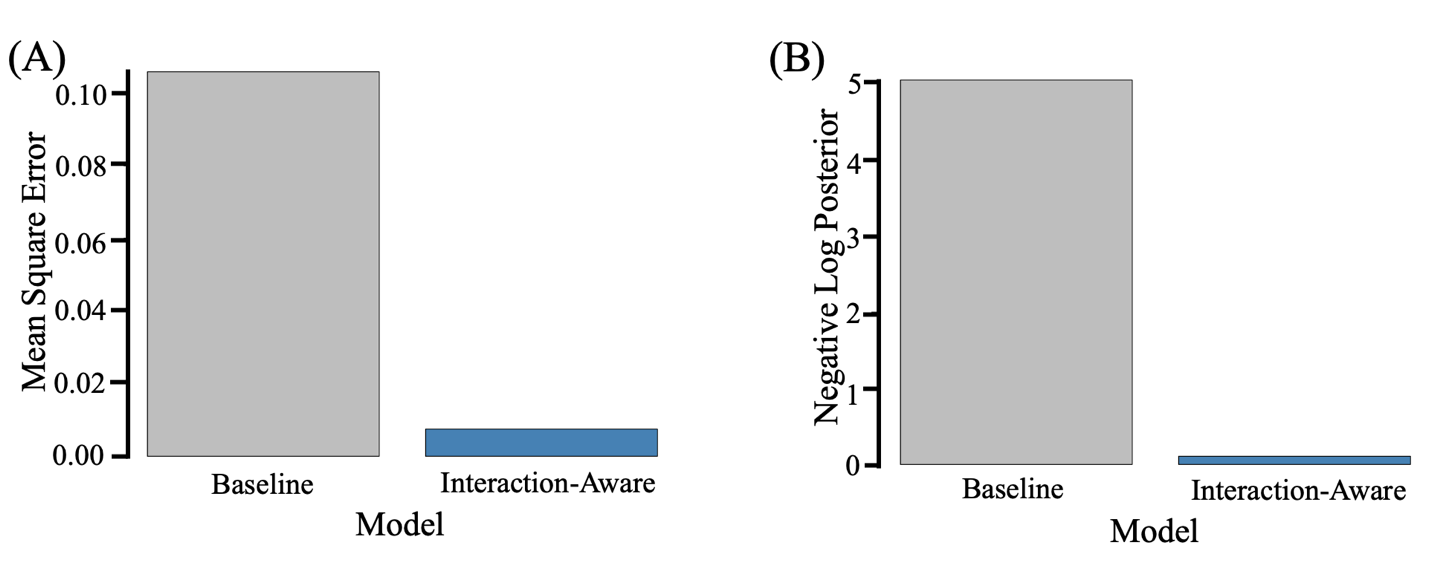


**S2 Fig. Evaluation of prediction accuracy using two metrics**. Mean Squared Error (MSE) and Negative Log Posterior (NLP). (A) MSE, which quantifies the average squared difference between predicted and true gene expression levels; (B) NLP, capturing how well the model explains the observed data under the posterior distribution. Lower values in both metrics indicate improved model performance.

*Simulation Outcome 3: Posterior Calibration and Uncertainty Quantification*

We further assessed model performance using the negative log-posterior (NLP), shown in the S2 Fig. B. The NLP evaluates both the fidelity of the posterior mean and the plausibility of the inferred distribution given the observed data. The baseline model exhibited a much higher NLP value (approximately 5), suggesting poor posterior calibration and greater uncertainty in its predictions. In contrast, the interaction-aware model produced an NLP close to zero, indicating not only an accurate mean prediction but also a well-calibrated posterior distribution.

**Conclusion**

This simulation demonstrates that incorporating gene-gene interactions enhances prediction accuracy (lower MSE) and better explains observed data (lower NLP), underscoring the importance of $\left\langle\varphi\right\rangle$ in modeling cancer driver genes (CDGs). By embedding regulatory structure into the posterior, the interaction-aware model produces statistically and biologically more meaningful predictions. This section formalizes the mathematical framework for integrating domain knowledge into probabilistic inference, enabling more reliable CDG identification.
