# Supplementary figures and images for: PICDGI: A framework for predicting cancer driver genes through dynamic gene-gene interaction modeling of single-cell data
Source: PLoS Comput Biol. 2026 Apr 27;22(4):e1014143. doi: 10.1371/journal.pcbi.1014143 (PMC13119913; doi:10.1371/journal.pcbi.1014143)

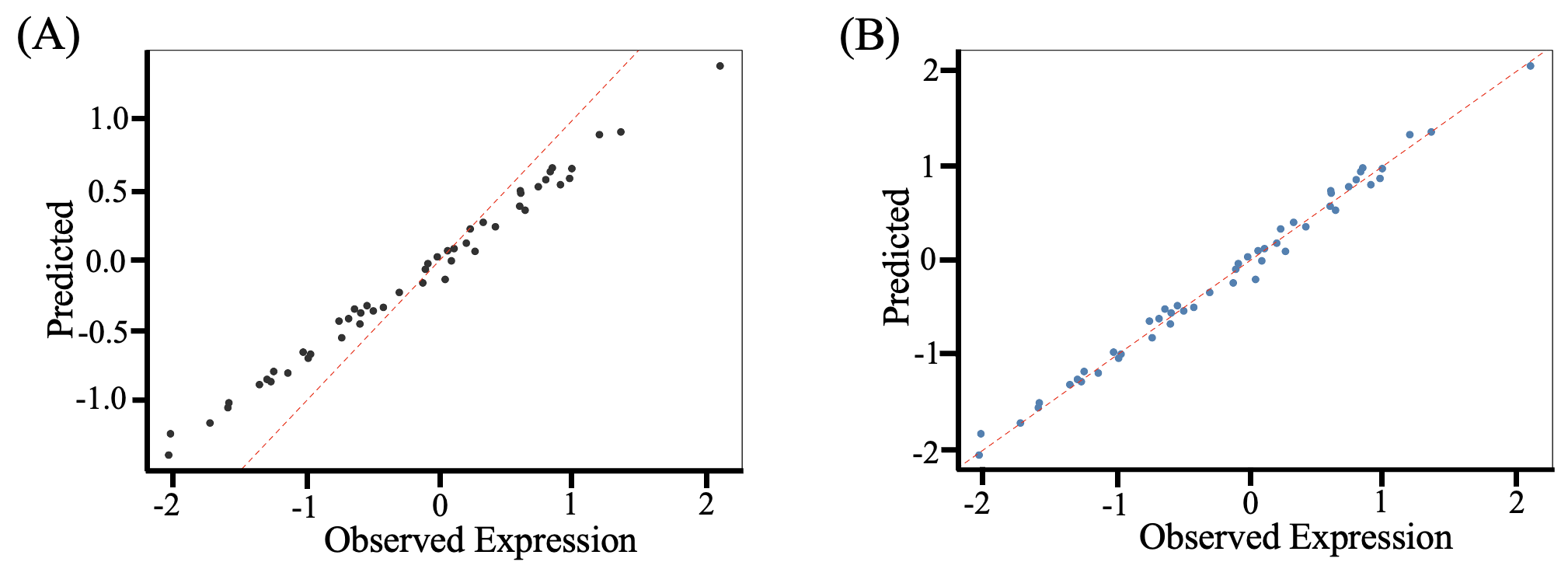

Supplement: S1 Fig — We compare predicted versus observed gene expression levels using a baseline model that assumes gene independence and an interaction-aware model that incorporates gene-gene interactions. (TIFF) [file pcbi.1014143.s005.tiff]

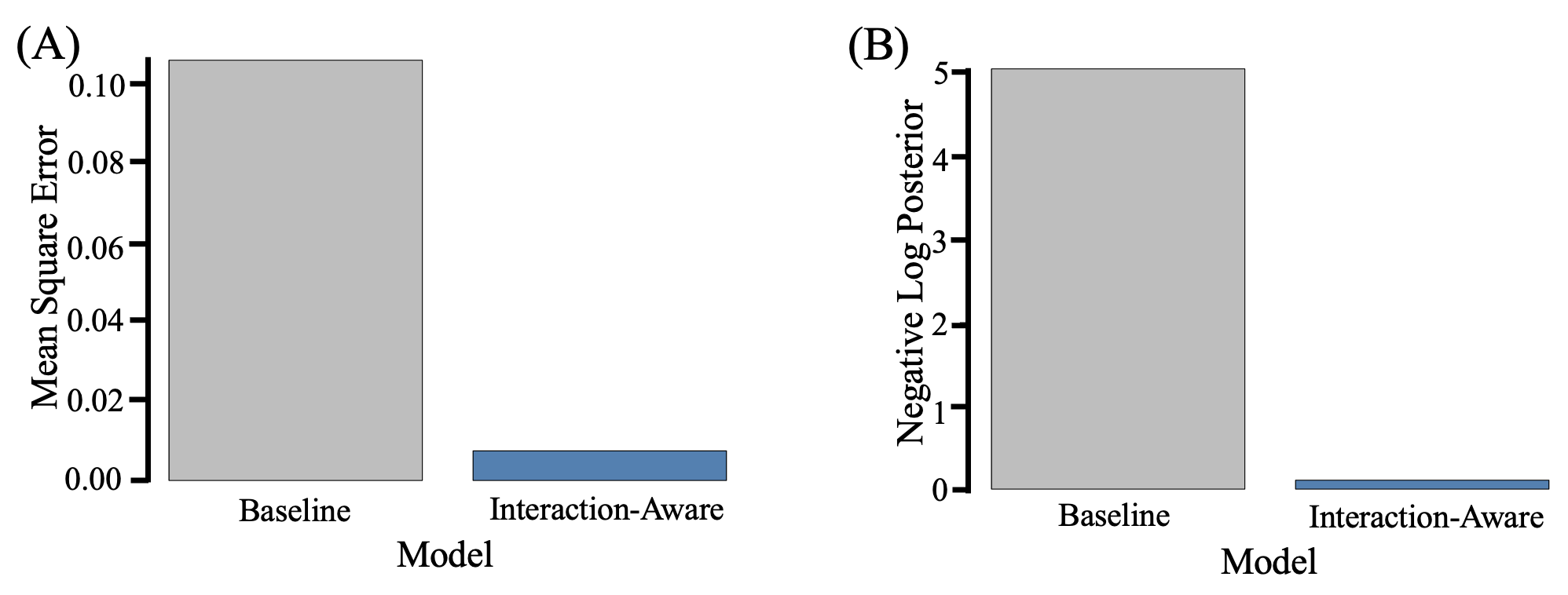

Supplement: S2 Fig — We evaluate prediction accuracy using two metrics: Mean Squared Error (MSE), which quantifies the average squared difference between predicted and observed gene expression levels, and Negative Log Posterior (NLP), which reflects how well the model explains the observed data under the posterior distribution. Lower values in both metrics indicate improved model performance. (TIFF) [file pcbi.1014143.s006.tiff]

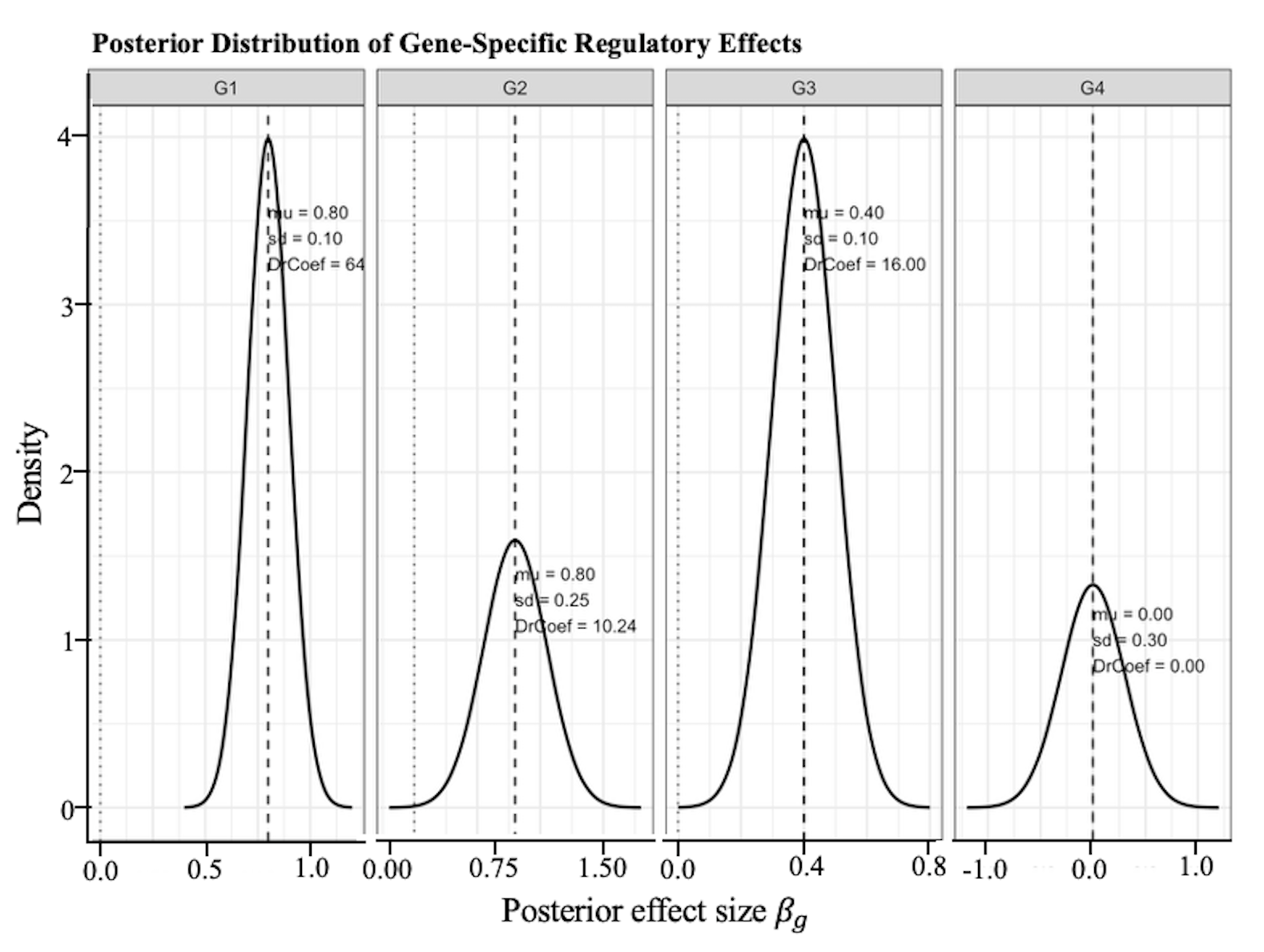

Supplement: S3 Fig — We show tSNE plots of marker gene expression across major cell lineages in early-stage Patient 1, highlighting lineage-specific markers for related immune and non-immune cells. (TIFF) [file pcbi.1014143.s017.tiff]

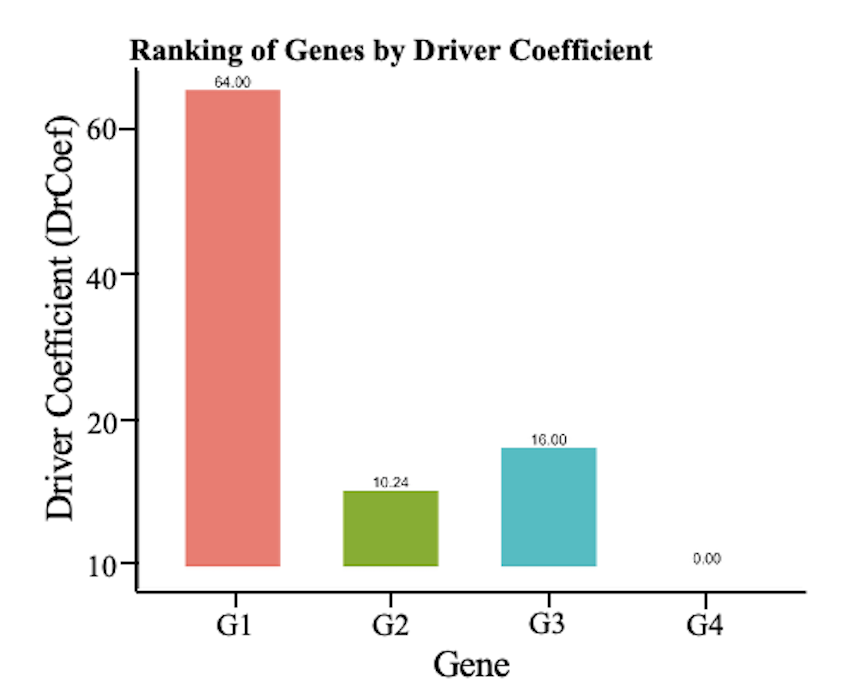

Supplement: S4 Fig — We show tSNE plots of marker gene expression across major cell lineages in early-stage Patient 2, highlighting lineage-specific markers for related immune and non-immune cells. (TIFF) [file pcbi.1014143.s018.tiff]

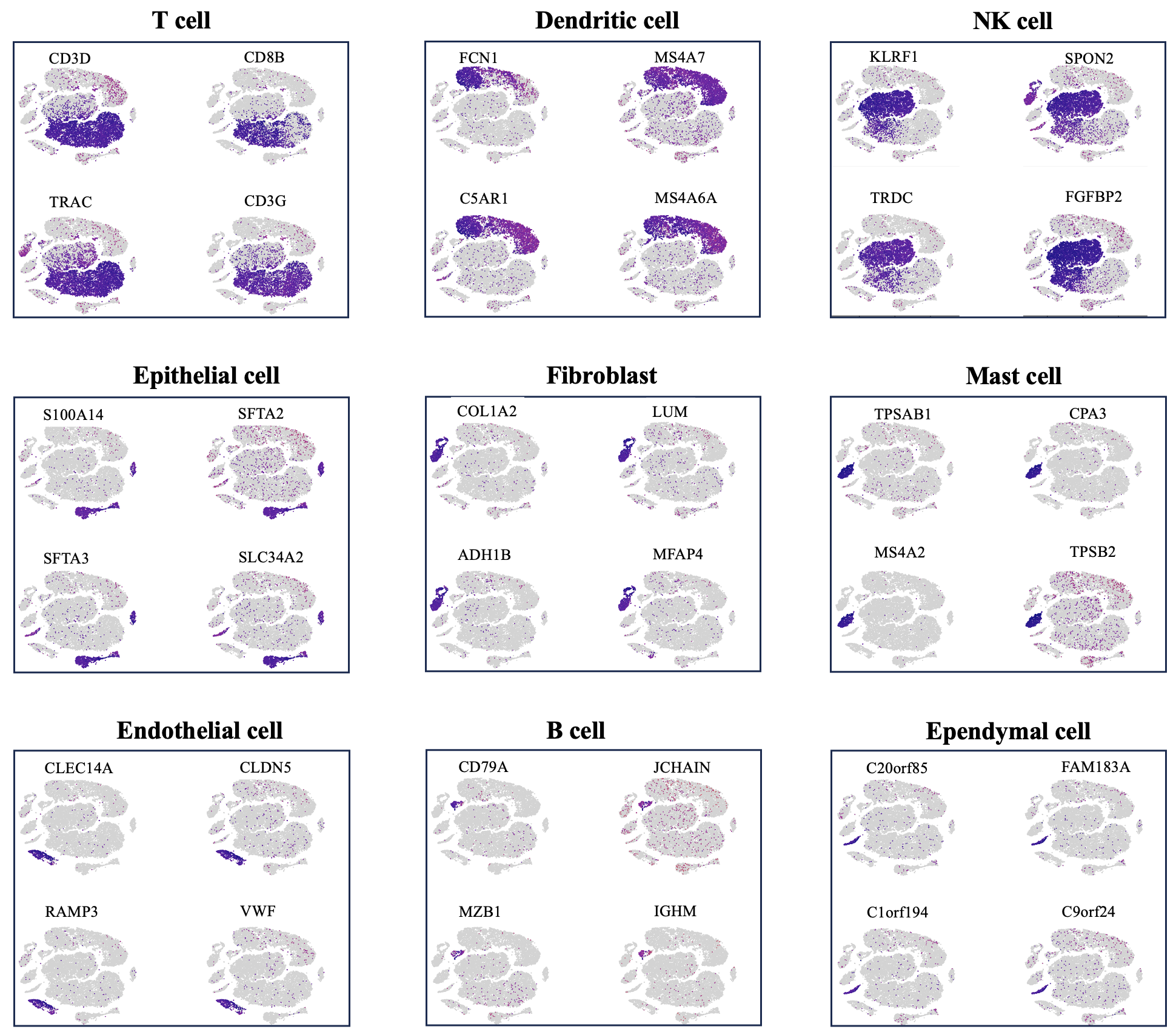

Supplement: S5 Fig — We show tSNE plots of marker gene expression across major cell lineages in early-stage Patient 2, highlighting lineage-specific markers for related immune and non-immune cells. (TIFF) [file pcbi.1014143.s019.tiff]

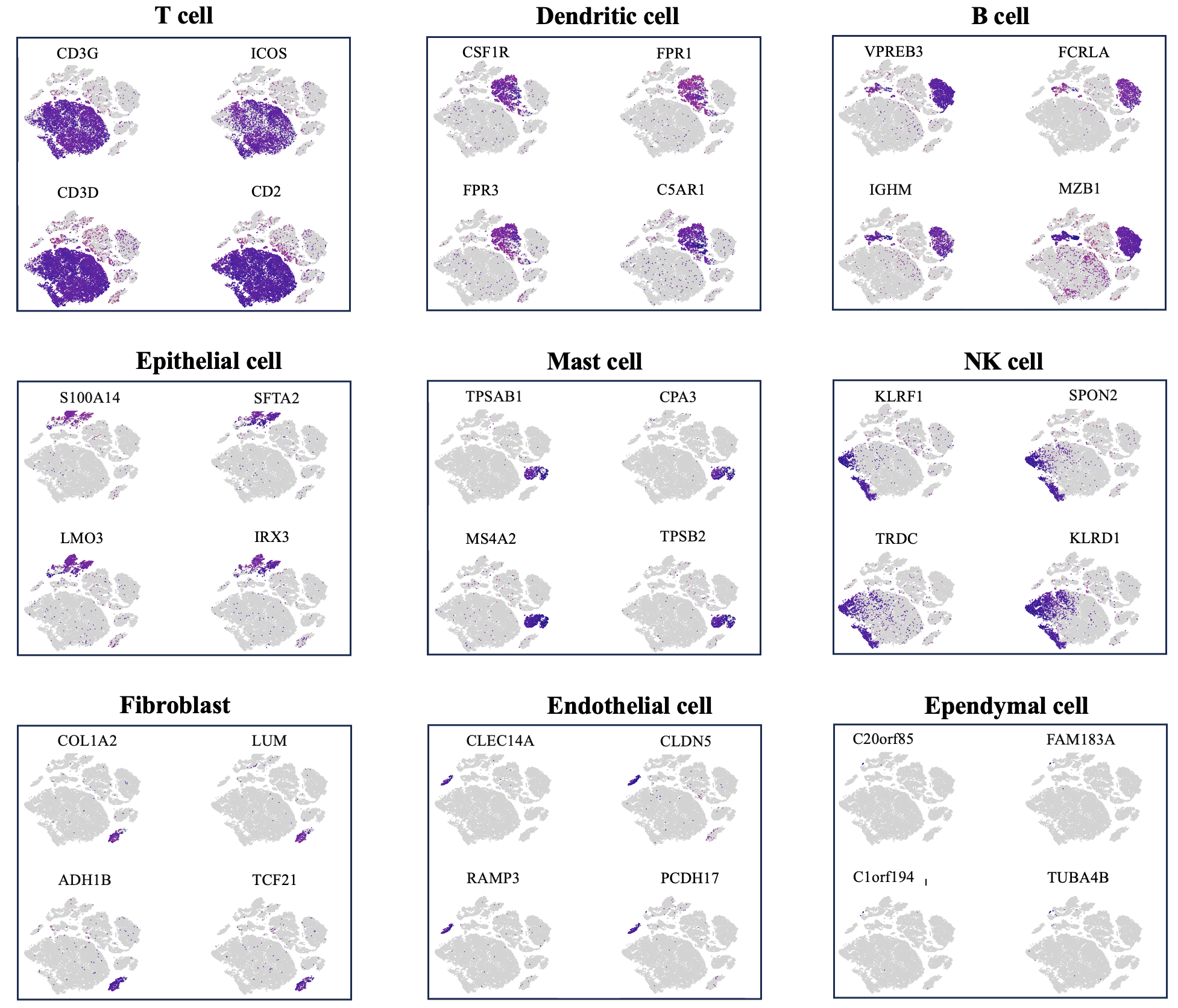

Supplement: S6 Fig — We show tSNE plots of marker gene expression across major cell lineages in early-stage Patient 2, highlighting lineage-specific markers for related immune and non-immune cells. (TIFF) [file pcbi.1014143.s020.tiff]

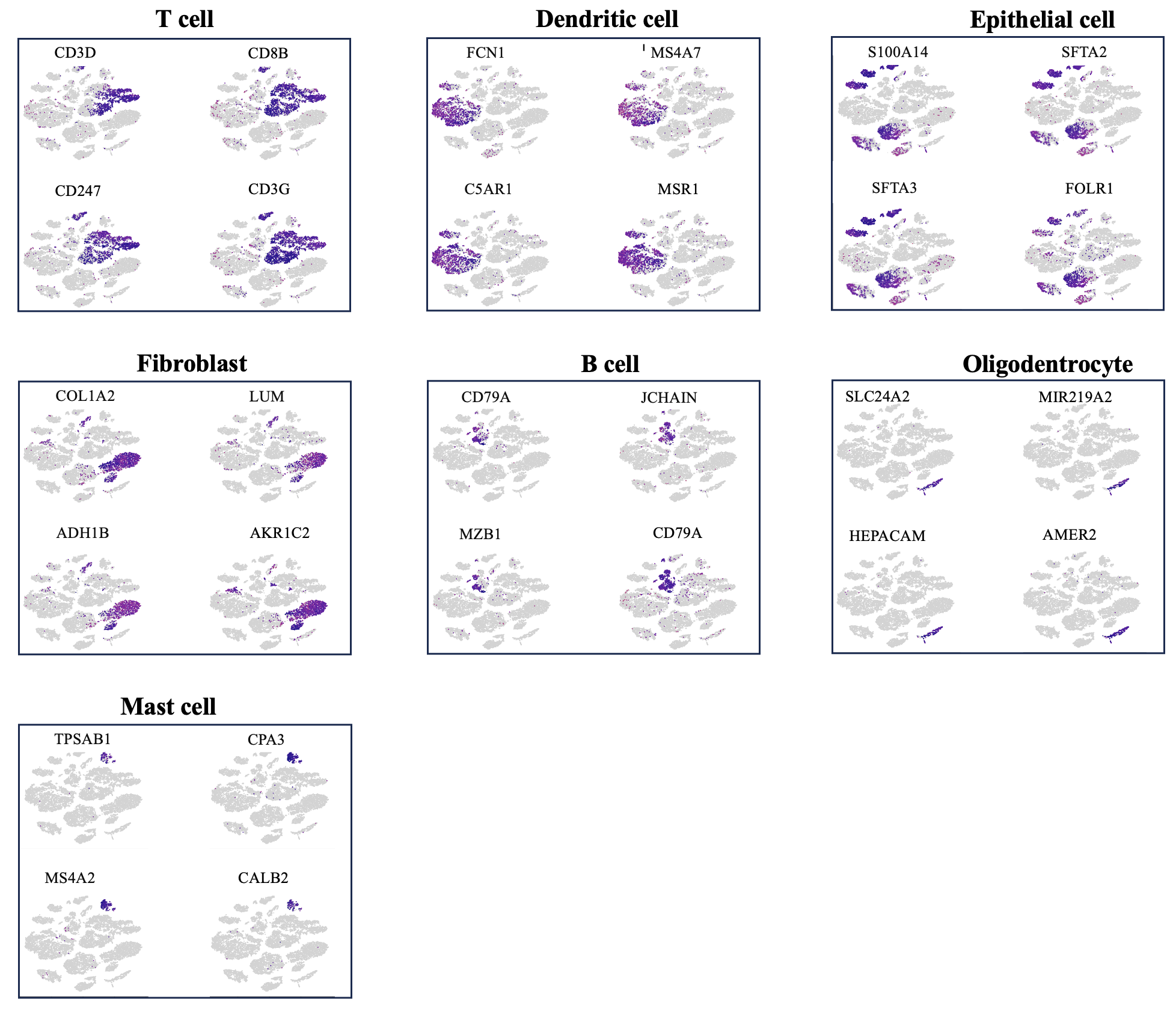

Supplement: S7 Fig — We show tSNE plots of marker gene expression across major cell lineages in early-stage Patient 3, highlighting lineage-specific markers for related immune and non-immune cells. (TIFF) [file pcbi.1014143.s021.tiff]

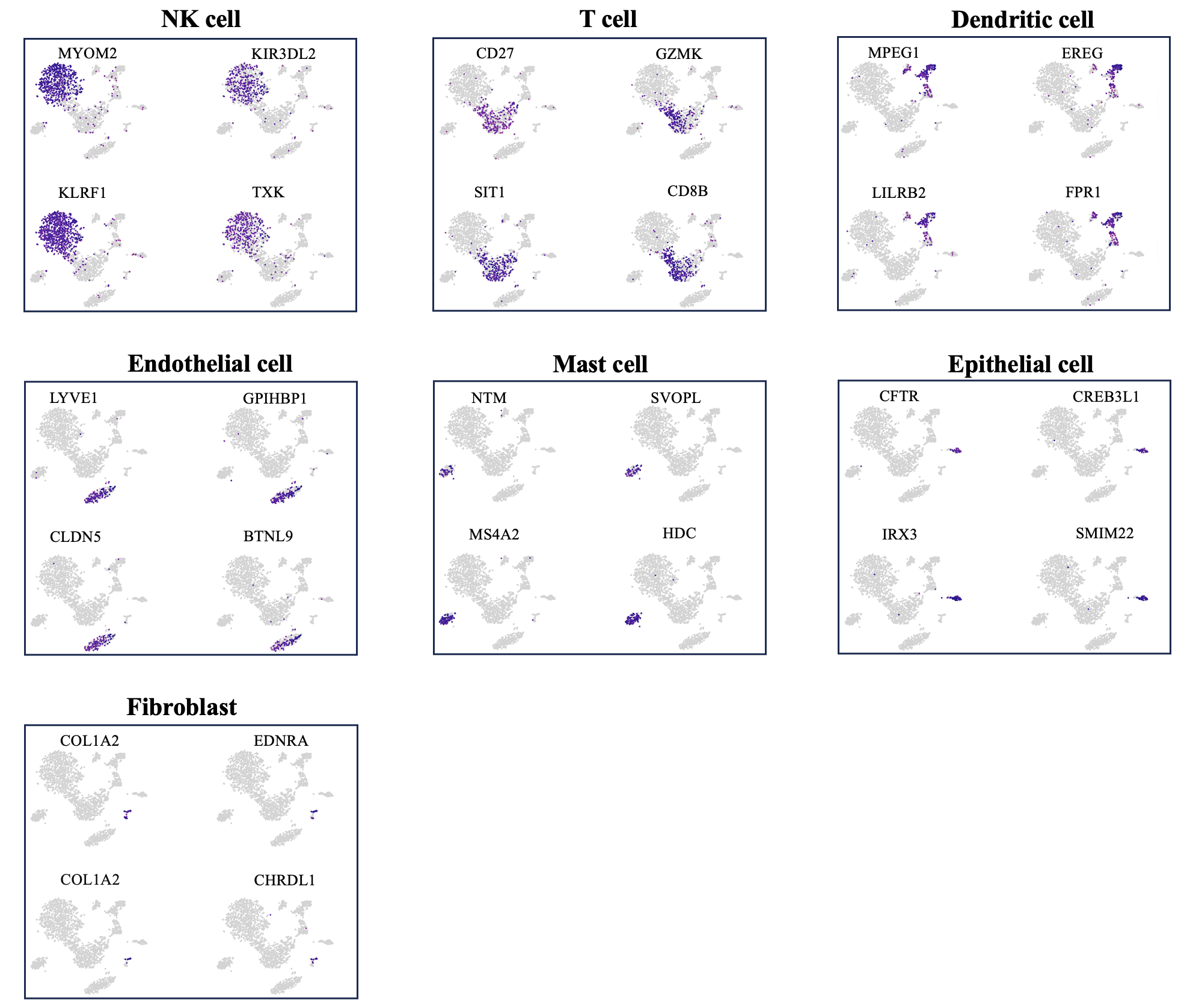

Supplement: S8 Fig — We show tSNE plots of marker gene expression across major cell lineages in early-stage Patient 3, highlighting lineage-specific markers for related immune and non-immune cells. (TIFF) [file pcbi.1014143.s022.tiff]

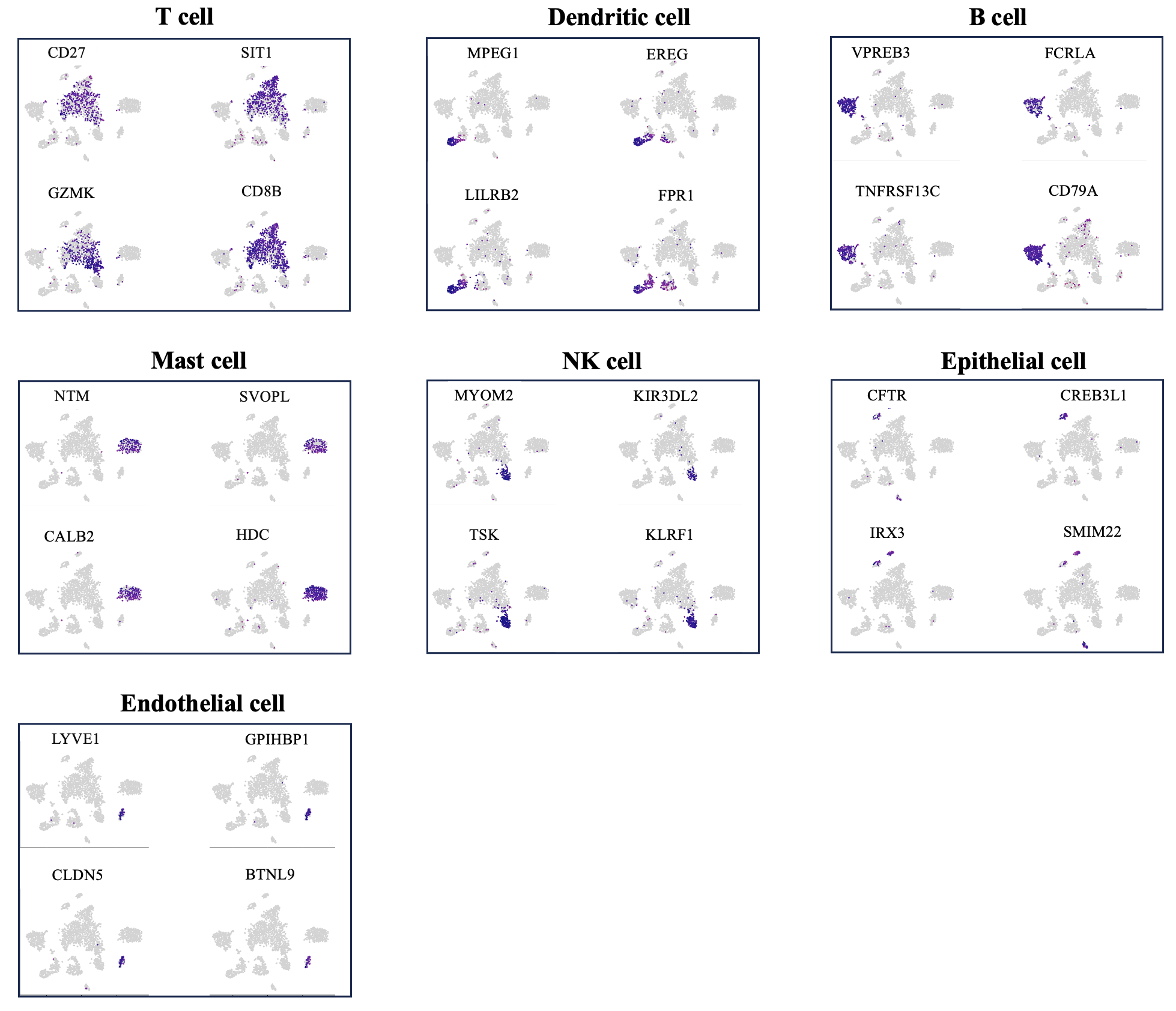

Supplement: S9 Fig — We show tSNE plots of marker gene expression across major cell lineages in early-stage Patient 3, highlighting lineage-specific markers for related immune and non-immune cells. (TIFF) [file pcbi.1014143.s023.tiff]

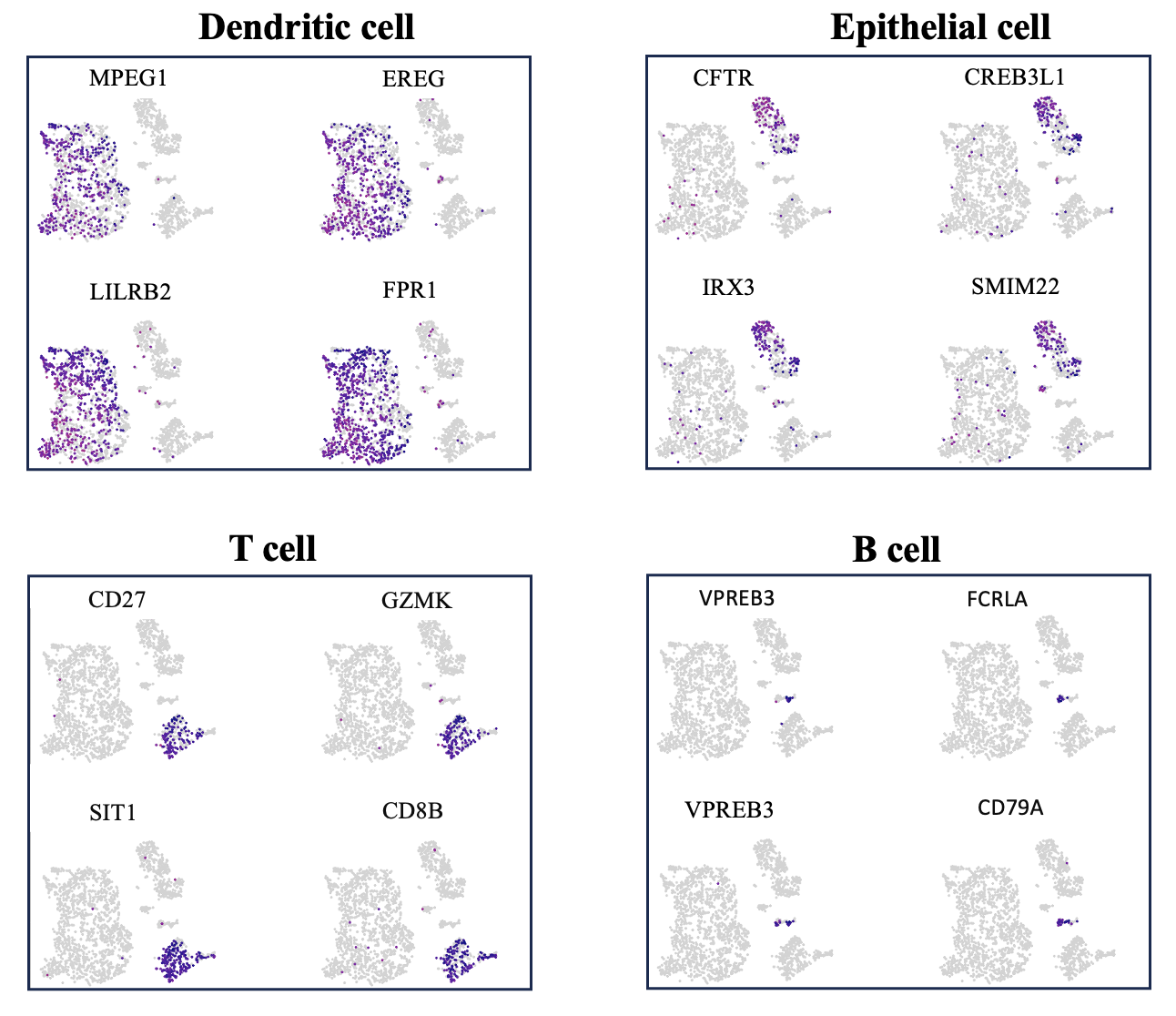

Supplement: S10 Fig — We illustrate epithelial cell genes with the highest driver coefficients for Patient 2, including a barplot highlighting known oncogenes and tumor suppressor genes, a heatmap showing gene driver inference across immune cell types from Patient 2’s single-cell data, and boxplots comparing transcription factor activity and expression between normal and cancer epithelial cells, revealing significant discordance in some cases supported by statistical tests. (TIFF) [file pcbi.1014143.s024.tiff]

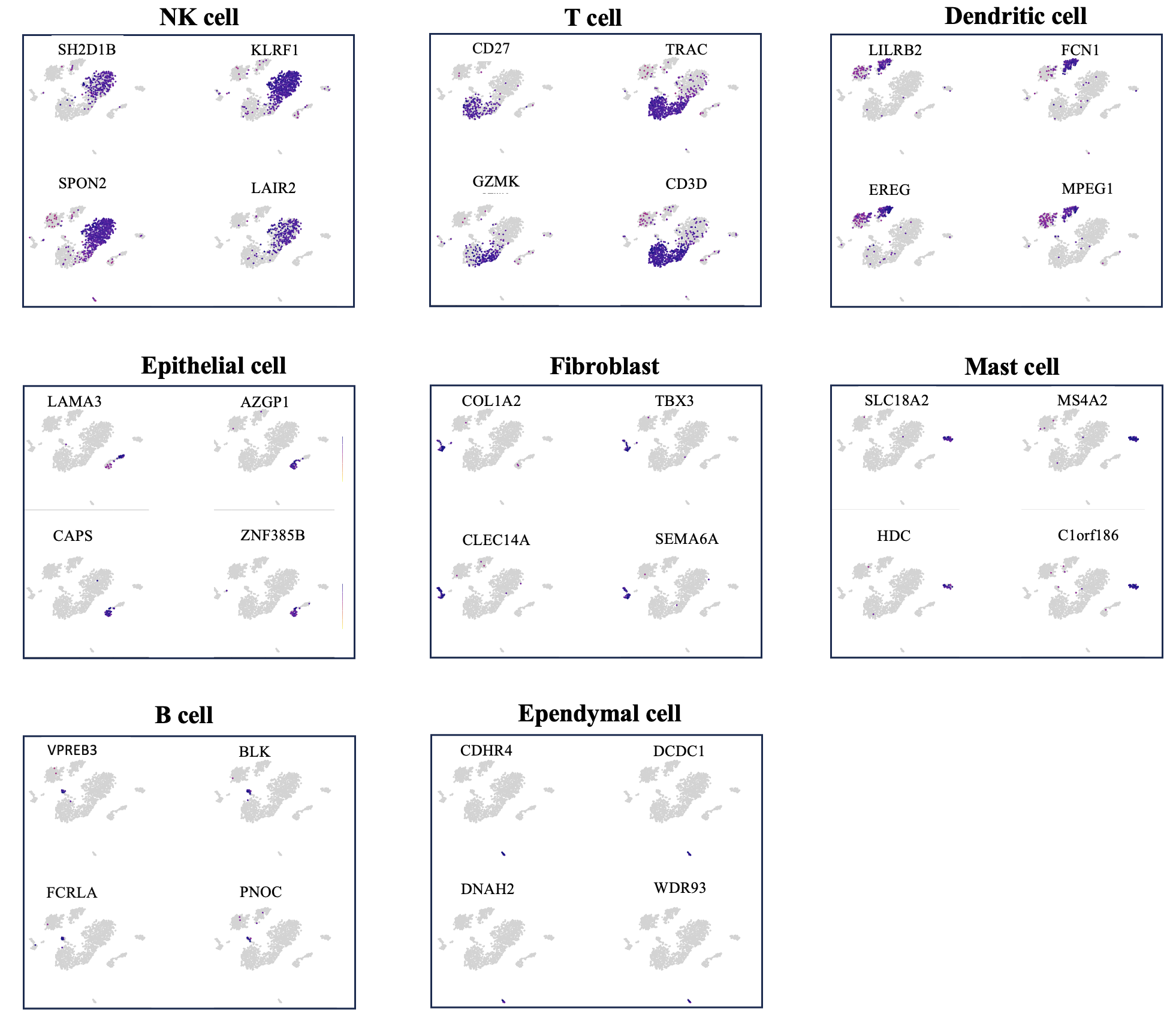

Supplement: S11 Fig — We illustrate epithelial cell genes with the highest driver coefficients for Patient 3, including a barplot highlighting known oncogenes and tumor suppressor genes, a heatmap showing gene driver inference across immune cell types from Patient 3’s single-cell data, and boxplots comparing transcription factor activity and expression between normal and cancer epithelial cells, revealing significant discordance in some cases supported by statistical tests. (TIFF) [file pcbi.1014143.s025.tiff]

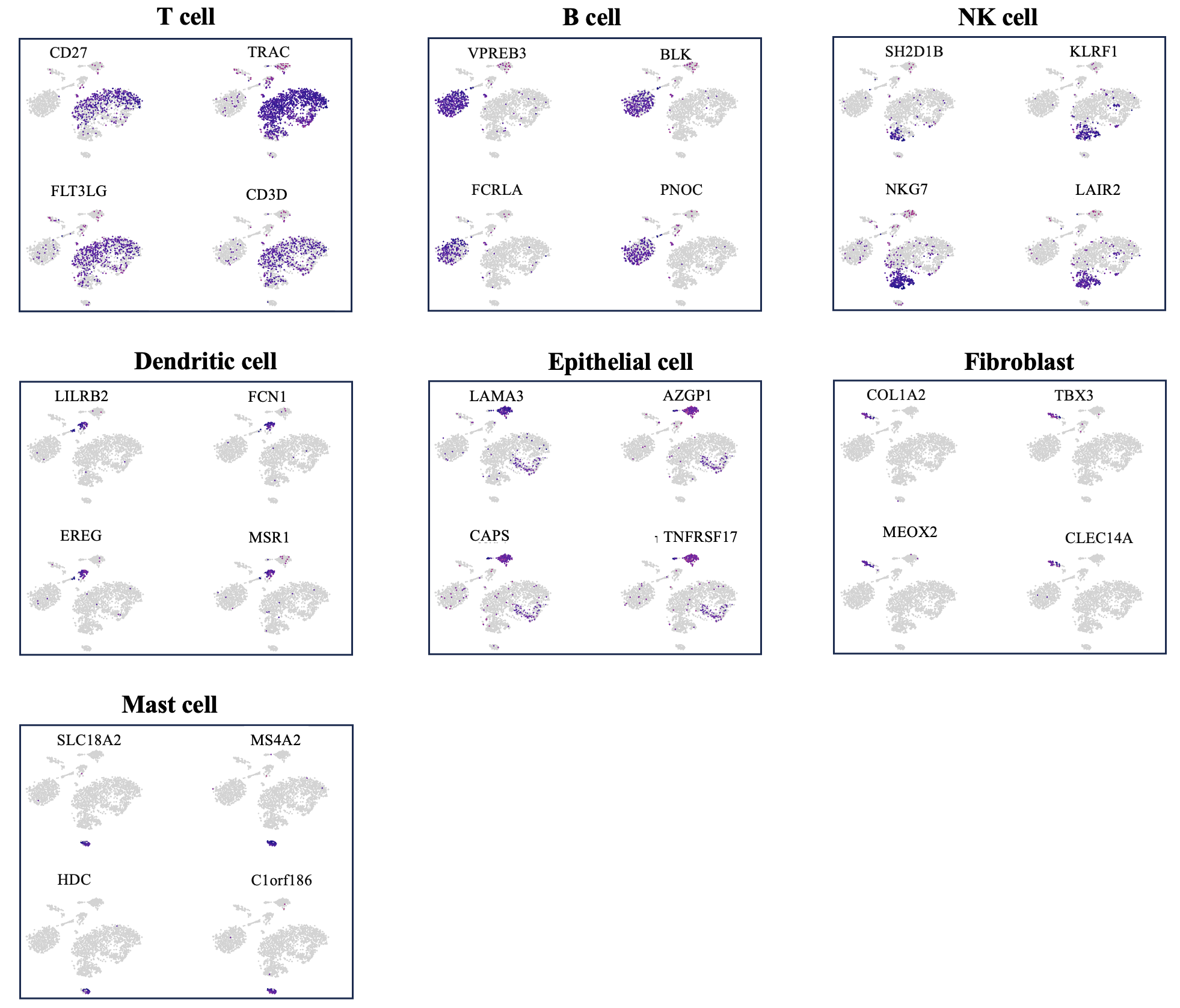

Supplement: S12 Fig — We compare the PICDGI framework with Moran’s I test for predicting driver genes in immune cells of Patient 2, finding that driver genes identified by Moran’s I have lower average expression levels than those from PICDGI, with genes ranked by immune-suppressive role across various cell types including mast cells, natural killer cells, T cells, B cells, and dendritic cells, alongside a single-cell atlas mapping cell progression and pseudo-time values. (TIFF) [file pcbi.1014143.s026.tiff]

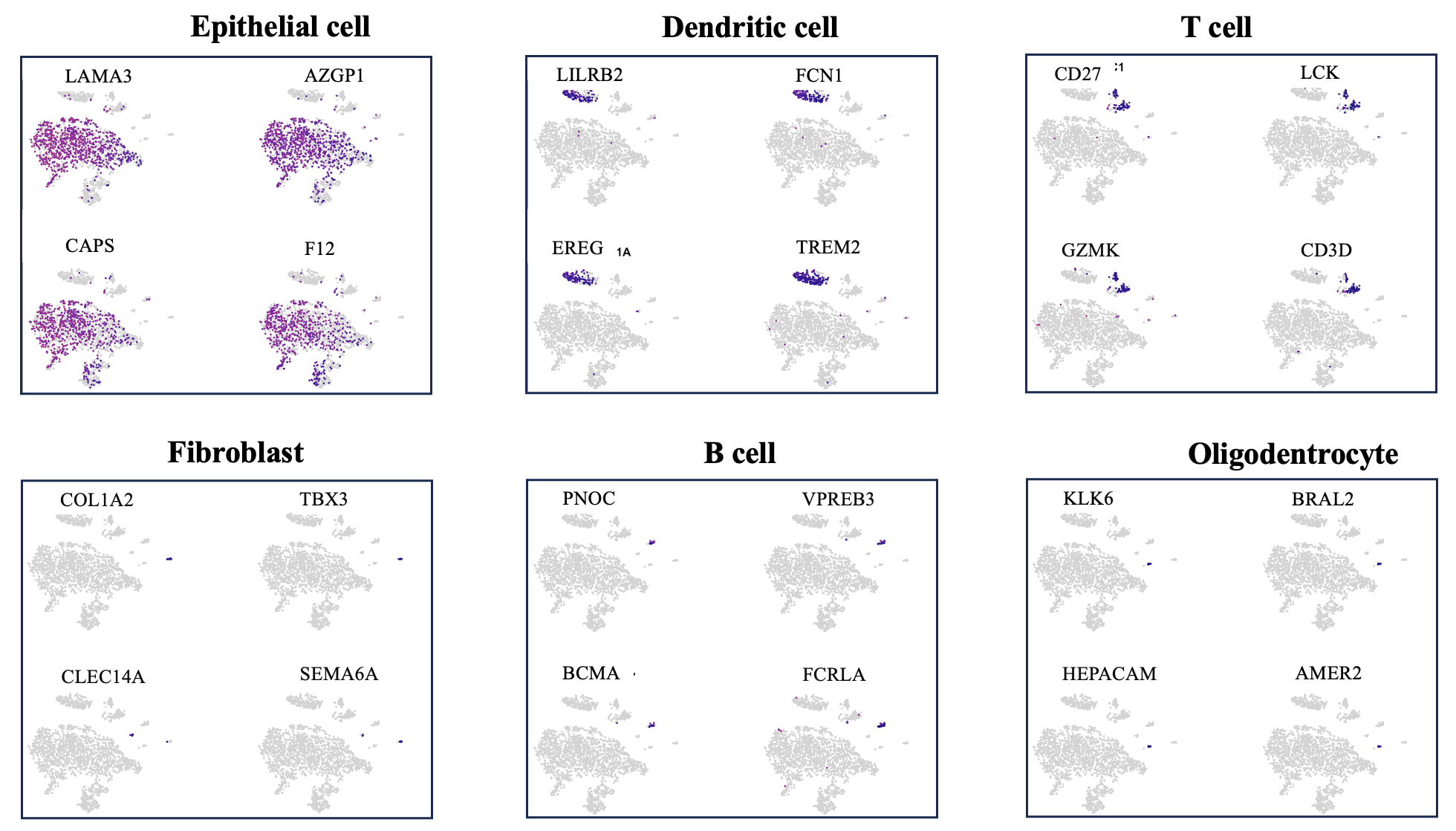

Supplement: S13 Fig — We compare the PICDGI framework with Moran’s I test for predicting driver genes in immune cells of Patient 3, finding that driver genes identified by Moran’s I have lower average expression levels than those from PICDGI, with genes ranked by immune-suppressive role across various cell types including mast cells, natural killer cells, T cells, B cells, and dendritic cells, alongside a single-cell atlas mapping cell progression and pseudo-time values. (TIFF) [file pcbi.1014143.s027.tiff]
