# Supplementary material for: PICDGI: A framework for predicting cancer driver genes through dynamic gene-gene interaction modeling of single-cell data
Source: PLoS Comput Biol. 2026 Apr 27;22(4):e1014143. doi: 10.1371/journal.pcbi.1014143 (PMC13119913; doi:10.1371/journal.pcbi.1014143)
Supplement: S5 Text — (DOCX) [file pcbi.1014143.s007.docx]

**S5 Text. Conceptual Illustration of the Driver Coefficient (DrCoef)**

The Driver Coefficient (DrCoef) is the central metric used by PICDGI to quantify each gene’s influence on dynamic gene-gene interactions during cancer progression. Because DrCoef underlies the ranking of candidate driver genes, it is important to provide intuition for how it is computed and why it distinguishes strong, reliable regulators from weak or uncertain ones. This supplement presents a simplified derivation of DrCoef from the posterior distribution of a gene’s regulatory effect, together with a toy example and two schematic figures.

### **Posterior Distribution of Gene-Specific Regulatory Effects**

### For each gene $\boldsymbol{g}$, PICDGI infers a latent regulatory effect parameter $\boldsymbol{\beta}_{\boldsymbol{g}}$ that represents the gene’s contribution to the temporal evolution of gene expression dynamics. Bayesian inference yields a posterior distribution over this parameter, incorporating both observed data and model uncertainty:

$$\beta_{g}|\text{data}\sim p\left( \beta_{g}|\text{gene expression trajectories} \right) (1)$$

For illustrative purposes, we approximate the posterior as a normal distribution,

$$\beta_{g}\mathcal{\sim N}\left( \mu_{g},\sigma_{g}^{2} \right), (2)$$

Where

- $\mu_{g}$is the posterior mean (estimated effect magnitude), and
- $\sigma_{g}$is the posterior standard deviation (uncertainty in the estimate).

These two quantities together determine how strongly the model believes gene $g$ influences the cancer progression process.

### **Definition of DrCoef**

### PICDGI defines the Driver Coefficient as:

$$\text{DrCoef}_{g}=\left( \frac{\mu_{g}}{\sigma_{g}} \right)^{2}. (3)$$

This expression is equivalent to the squared signal-to-noise ratio. It reflects the principle that a gene should be ranked highly only when it exhibits both:

- A large, estimated effect ($\left| \mu_{g} \right|$ is large), and
- High certainty in that estimate ($\sigma_{g}$ is small).

A gene with a large mean but high uncertainty, or a gene with low mean even if variance is small, will therefore not achieve a high DrCoef. This ensures that DrCoef rewards both **regulatory strength** and **statistical reliability**.

### **Toy Example Demonstrating DrCoef Behavior**

### To visualize the interplay between posterior mean and variance, we consider four example genes with hypothetical posterior parameters (Table S1):

**S1 Table.** Posterior Mean, Posterior Variability, and Effect Interpretation for Representative Genes

| **Gene** | **Posterior Mean** $\boldsymbol{\mu}_{\boldsymbol{g}}$ | **Posterior SD** $\boldsymbol{\sigma}_{\boldsymbol{g}}$ | **Interpretation** |
| --- | --- | --- | --- |
| G1 | 0.80 | 0.10 | Strong well-estimated effect |
| G2 | 0.80 | 0.25 | Strong but less certain effect |
| G3 | 0.40 | 0.10 | Moderate, well-estimated effect |
| G4 | 0.00 | 0.30 | No effect, high uncertainty |

Applying the DrCoef formula,

$$\text{DrCoef}_{g}=\left( \frac{\mu_{g}}{\sigma_{g}} \right)^{2}, (4)$$

We obtained the driver coefficients and interpretive categories shown in Table S2.

**S2 Table.** Ranking of Gene-Level Driver Coefficients and Their Biological Interpretation

| **Gene** | **DrCoef** | **Interpretation** |
| --- | --- | --- |
| G1 | 64.00 | Highest driver: large effect, high certainty |
| G2 | 10.24 | Moderate driver: smaller effect but highly certain |
| G3 | 16 | Lower rank due to high uncertainty |
| G4 | 0.00 | Negligible driver activity |

This toy example illustrates several important points. First, although G1 and G2 have the same posterior mean, the larger variance of G2 substantially reduces its DrCoef. Second, G3, despite having a smaller effect size than G2, receives a higher DrCoef than G2 because its effect is estimated more precisely. Finally, G4 exemplifies how genes with negligible effects, even when moderately uncertain, correctly receive low DrCoef values. This confirms that DrCoef balances effect magnitude and confidence in a principled way.

**Visualization of Posterior Densities and Gene Rankings**

To further clarify the computation and interpretation of DrCoef, Supplementary S1 Fig. displays the posterior density curves of the four example genes. Each curve shows the distribution of possible effect sizes, with narrow, peaked densities corresponding to low variance (high certainty) and wider curves corresponding to greater uncertainty. The posterior mean is indicated by a dashed line, and zero effect is shown by a dotted reference line. Comparing these plots makes it visually apparent how differences in uncertainty influence DrCoef.

# **
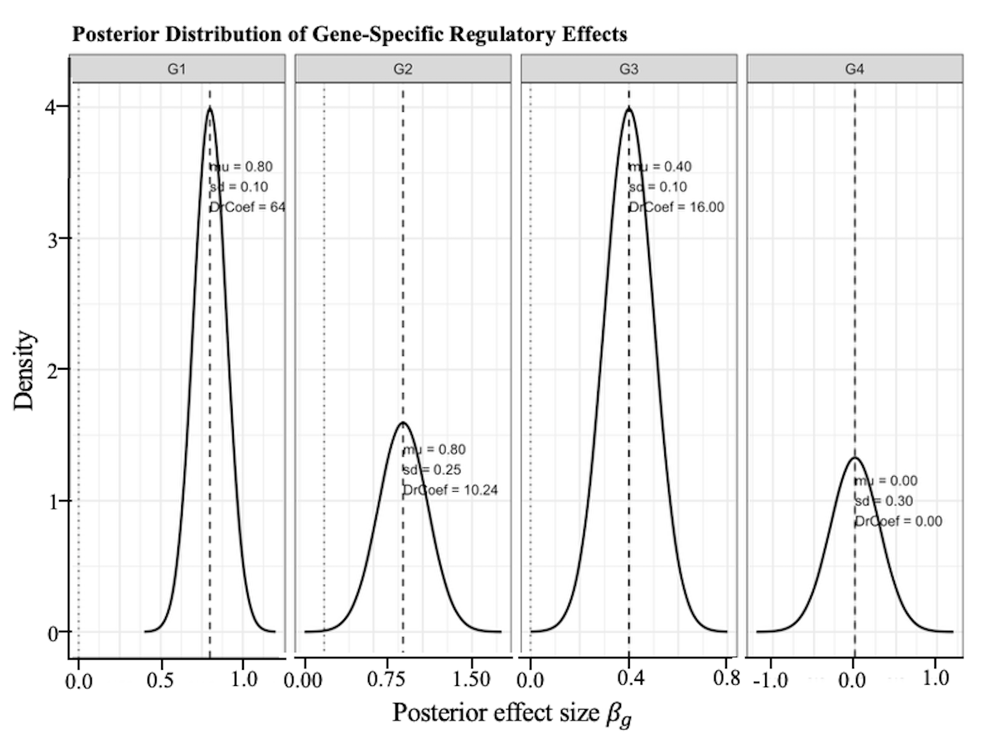
**

# **S1 Fig. Posterior Distributions of Gene-Specific Regulatory Effects (Toy Example).** Each panel displays the posterior density of the regulatory effect parameter $\beta_{g}$for one of the four toy genes, modeled as a normal distribution with mean $\mu_{g}$and standard deviation $\sigma_{g}$. The dashed vertical line indicates the posterior mean, and the dotted vertical line marks zero effect. Narrower curves indicate greater certainty. The displayed values of $\mu_{g}$, $\sigma_{g}$, and the resulting DrCoef illustrate how certainty and effect magnitude jointly determine the ranking of genes.

Supplementary S2 Fig. presents a barplot of the computed DrCoef values for the four genes. This ranking visually reinforces the logic of the metric: G1, with the strongest and most certain effect, ranks highest; G3, with moderate but precise effect, ranks second; G2, whose effect is strong but uncertain, ranks below G3; and G4 receives a DrCoef of zero due to lack of effect. Together, these figures provide an intuitive framework for understanding how PICDGI uses posterior distributions to highlight putative cancer drivers.

# **
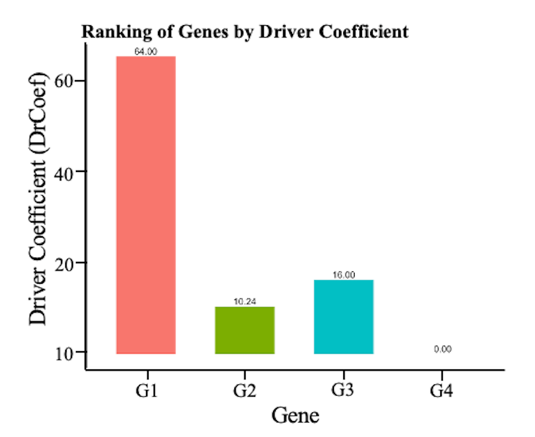
**

# **S2 Fig. Ranking of Genes by Driver Coefficient (DrCoef).** Barplot showing the Driver Coefficient for the four toy genes computed as $\text{DrCoef}_{g}=(\mu_{g}/\sigma_{g})^{2}$. G1 has the highest DrCoef due to both a large posterior mean and low variance, while G3 ranks above G2 because it has lower uncertainty despite a smaller effect size. Gene G4 exhibits no effect and therefore receives a DrCoef of zero. This visualization demonstrates how DrCoef integrates effect size and uncertainty to prioritize potential driver genes.
