# Supplementary material for: PICDGI: A framework for predicting cancer driver genes through dynamic gene-gene interaction modeling of single-cell data
Source: PLoS Comput Biol. 2026 Apr 27;22(4):e1014143. doi: 10.1371/journal.pcbi.1014143 (PMC13119913; doi:10.1371/journal.pcbi.1014143)
Supplement: S1 Table — (DOCX) [file pcbi.1014143.s008.docx]

**S1 Table.** Posterior Mean, Posterior Variability, and Effect Interpretation for Representative Genes

| **Gene** | **Posterior Mean** $\boldsymbol{\mu}_{\boldsymbol{g}}$ | **Posterior SD** $\boldsymbol{\sigma}_{\boldsymbol{g}}$ | **Interpretation** |
| --- | --- | --- | --- |
| G1 | 0.80 | 0.10 | Strong well-estimated effect |
| G2 | 0.80 | 0.25 | Strong but less certain effect |
| G3 | 0.40 | 0.10 | Moderate, well-estimated effect |
| G4 | 0.00 | 0.30 | No effect, high uncertainty |
