# Supplementary material for: PICDGI: A framework for predicting cancer driver genes through dynamic gene-gene interaction modeling of single-cell data
Source: PLoS Comput Biol. 2026 Apr 27;22(4):e1014143. doi: 10.1371/journal.pcbi.1014143 (PMC13119913; doi:10.1371/journal.pcbi.1014143)
Supplement: S2 Table — (DOCX) [file pcbi.1014143.s009.docx]

**S2 Table.** Ranking of Gene-Level Driver Coefficients and Their Biological Interpretation

| **Gene** | **DrCoef** | **Interpretation** |
| --- | --- | --- |
| G1 | 64.00 | Highest driver: large effect, high certainty |
| G2 | 10.24 | Lower rank due to high uncertainty |
| G3 | 16 | Moderate driver: smaller effect but highly certain |
| G4 | 0.00 | Negligible driver activity |
