# Supplementary material for: PICDGI: A framework for predicting cancer driver genes through dynamic gene-gene interaction modeling of single-cell data
Source: PLoS Comput Biol. 2026 Apr 27;22(4):e1014143. doi: 10.1371/journal.pcbi.1014143 (PMC13119913; doi:10.1371/journal.pcbi.1014143)
Supplement: S6 Text — (DOCX) [file pcbi.1014143.s012.docx]

**S6 Text. Comparing PICDGI Features Against Existing Cancer Driver-Discovery and Dynamic Network-Inference Frameworks**

# *PICDGI* operates directly on single-cell RNA-seq (scRNA-seq) data and uses time-aware (trajectory-based) modeling to rank candidate driver genes. The inputs consist of single-cell transcriptomes annotated with pseudotime, discrete staging time points, or short-step progression trajectories. The outputs include driver gene rankings and driver-centered influence networks. The framework is inherently longitudinal and is specifically designed to function without mutation level data, or matched DNA sequencing or additional omics data.

The S1 Table summarizes the key distinctions between ***PICDGI*** and representative method families used for cancer driver identification. It highlights input data requirements, time-awareness, applicability to single-cell RNA sequencing (scRNA-seq), dependency on matched DNA data, and the ability to return explicit driver-modulator pairs.

**S1 Table.** Comparative Summary of PICDGI and Existing Cancer Driver-Discovery and Network-Inference Method Families


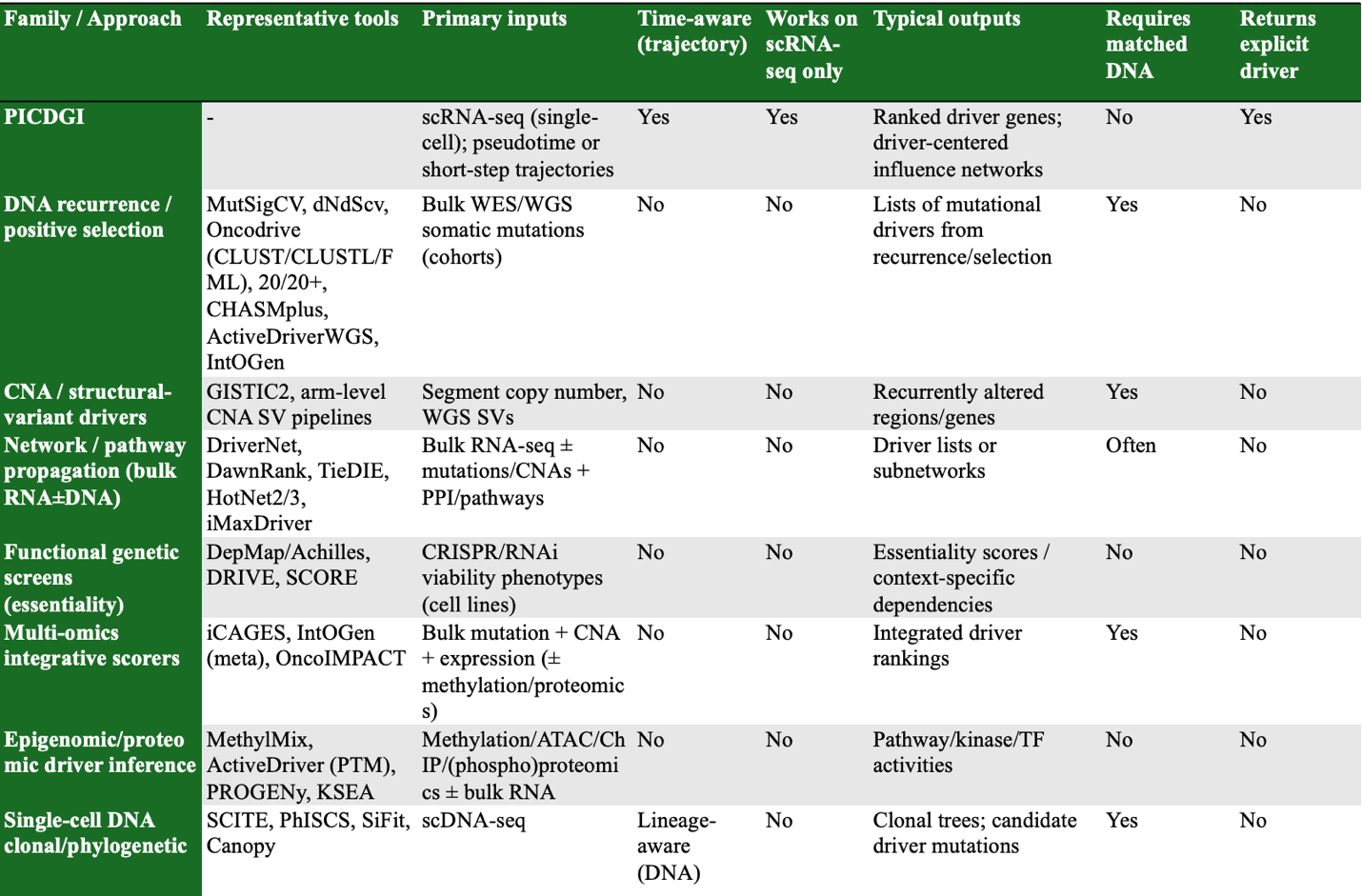


***DNA recurrence/positive-selection frameworks.*** Methods such as MutSigCV, dNdScv, OncodriveCLUST/CLUSTL, OncodriveFML, 20/20+, CHASMplus, ActiveDriverWGS, and IntOGen analyze WES/WGS somatic variants at the cohort level to call genes under positive selection (e.g., recurrent or clustered SNVs/indels, elevated dN/dS). They output lists of mutational drivers inferred from recurrence/selection signals. These are not time-aware and do not operate on scRNA-seq data alone; they require DNA mutation data[1].

***Copy-number alteration (CNA) / structural-variant (SV) drivers.*** Tools like GISTIC2 (and related CNA/SV pipelines) work on segmented copy-number profiles or WGS structural variants to identify recurrently altered genomic regions/genes as putative drivers. Outputs are driver regions/genes based on CNA/SV recurrence. These frameworks require DNA copy-number/SV input and are not applicable to scRNA-seq-only settings[2].

***Network and pathway propagation (bulk RNA±DNA).*** Approaches such as DriverNet, DawnRank, TieDIE, HotNet2/3, and iMaxDriver integrate bulk RNA-seq (often with mutation/CNA) and PPI/pathway networks to prioritize drivers whose effects propagate through interaction graphs. They typically output driver lists or subnetworks. While sometimes adaptable to pseudo-bulk, they are not designed for single-cell resolution and do not return explicit driver-modulator pairs[3].

***Functional genetic screens (essentiality).*** Large-scale CRISPR/RNAi projects (e.g., DepMap/Achilles, DRIVE, SCORE) use loss-of-function viability data in cell lines to define essential/fitness genes that can be interpreted as drivers in specific contexts. Inputs are perturbation phenotypes; outputs are essentiality scores. These systems differ fundamentally from primary-tissue scRNA-seq and do not provide transcriptome-based driver–modulator relationships[4].

***Multi-omics integrative scorers.*** Methods like iCAGES, IntOGen (meta-pipelines), OncoIMPACT, and related tools combine mutation + CNA + expression (and sometimes methylation/proteomics) in bulk to yield integrated driver rankings under the assumption that concordant aberrations across omics mark drivers. They are not scRNA-seq-only, are not inherently time-aware, and do not emit driver-modulator pairs[5].

***Epigenomic/proteomic driver inference.*** Frameworks including MethylMix (DNA methylation), ActiveDriver (post-translational modification), and pathway-activity estimators (e.g., PROGENy, kinase-activity/KSEA) use methylation/ATAC/ChIP/(phospho)proteomics with bulk expression to infer driver pathways/kinases/TFs. These provide pathway-level modulators or regulators but not gene-specific driver-modulator pairs from scRNA-seq[6].

***Single-cell DNA clonal/phylogenetic tools.*** Methods such as SCITE, PhISCS, SiFit, and Canopy analyze single-cell DNA-seq to reconstruct clonal trees and infer mutations that seed or expand clones (driver mutations). They are explicitly time/lineage-aware on the DNA axis, but they do not consume scRNA-seq nor produce transcriptomic driver–modulator outputs[7, 8].

***scRNA-seq regulatory/GRN & trajectory tools (related but different objective***). Tools like SCENIC/pySCENIC, GRNBoost2, PIDC, Monocle, Slingshot, and tradeSeq operate on scRNA-seq to infer regulatory modules (regulons/GRNs) or smooth differential patterns along pseudotime. They can be partially comparable on modality but pursue a different goal (regulatory module discovery), and they do not define causal driver genes or explicit non-driver modulators as pairs[9].

Synthesis and novelty of *CancerTrace*. Across these families, most established frameworks depend on DNA or multi-omic inputs (mutation recurrence, CNA/SV, perturbation screens, epigenomics/proteomics) and bulk-level formulations. CancerTrace fills a clear gap by enabling driver discovery directly from scRNA-seq, incorporating dynamic (time-aware) modeling, and returning both drivers and their contextual

**References**

1. Rojas-Rodriguez F, Schmidt MK, Canisius S. Assessing the validity of driver gene identification tools for targeted genome sequencing data. Bioinformatics Advances. 2024;4(1):vbae073.

2. Mermel CH, Schumacher SE, Hill B, Meyerson ML, Beroukhim R, Getz G. GISTIC2. 0 facilitates sensitive and confident localization of the targets of focal somatic copy-number alteration in human cancers. Genome biology. 2011;12(4):R41.

3. Hou JP, Ma J. DawnRank: discovering personalized driver genes in cancer. Genome medicine. 2014;6(7):56.

4. Cheung HW, Cowley GS, Weir BA, Boehm JS, Rusin S, Scott JA, et al. Systematic investigation of genetic vulnerabilities across cancer cell lines reveals lineage-specific dependencies in ovarian cancer. Proceedings of the National Academy of Sciences. 2011;108(30):12372–7.

5. Dong C, Guo Y, Yang H, He Z, Liu X, Wang K. iCAGES: integrated CAncer GEnome Score for comprehensively prioritizing driver genes in personal cancer genomes. Genome medicine. 2016;8(1):135.

6. Zheng Y, Jun J, Brennan K, Gevaert O. EpiMix is an integrative tool for epigenomic subtyping using DNA methylation. Cell Reports Methods. 2023;3(7).

7. Jahn K, Kuipers J, Beerenwinkel N. Tree inference for single-cell data. Genome biology. 2016;17(1):86.

8. Malikic S, Mehrabadi FR, Ciccolella S, Rahman MK, Ricketts C, Haghshenas E, et al. PhISCS: a combinatorial approach for subperfect tumor phylogeny reconstruction via integrative use of single-cell and bulk sequencing data. Genome research. 2019;29(11):1860–77.

9. Aibar S, González-Blas CB, Moerman T, Huynh-Thu VA, Imrichova H, Hulselmans G, et al. SCENIC: single-cell regulatory network inference and clustering. Nature methods. 2017;14(11):1083–6.
