# Supplementary material for: PICDGI: A framework for predicting cancer driver genes through dynamic gene-gene interaction modeling of single-cell data
Source: PLoS Comput Biol. 2026 Apr 27;22(4):e1014143. doi: 10.1371/journal.pcbi.1014143 (PMC13119913; doi:10.1371/journal.pcbi.1014143)

**S1 Table.** Comparative Summary of PICDGI and Existing Cancer Driver-Discovery and Network-Inference Method Families


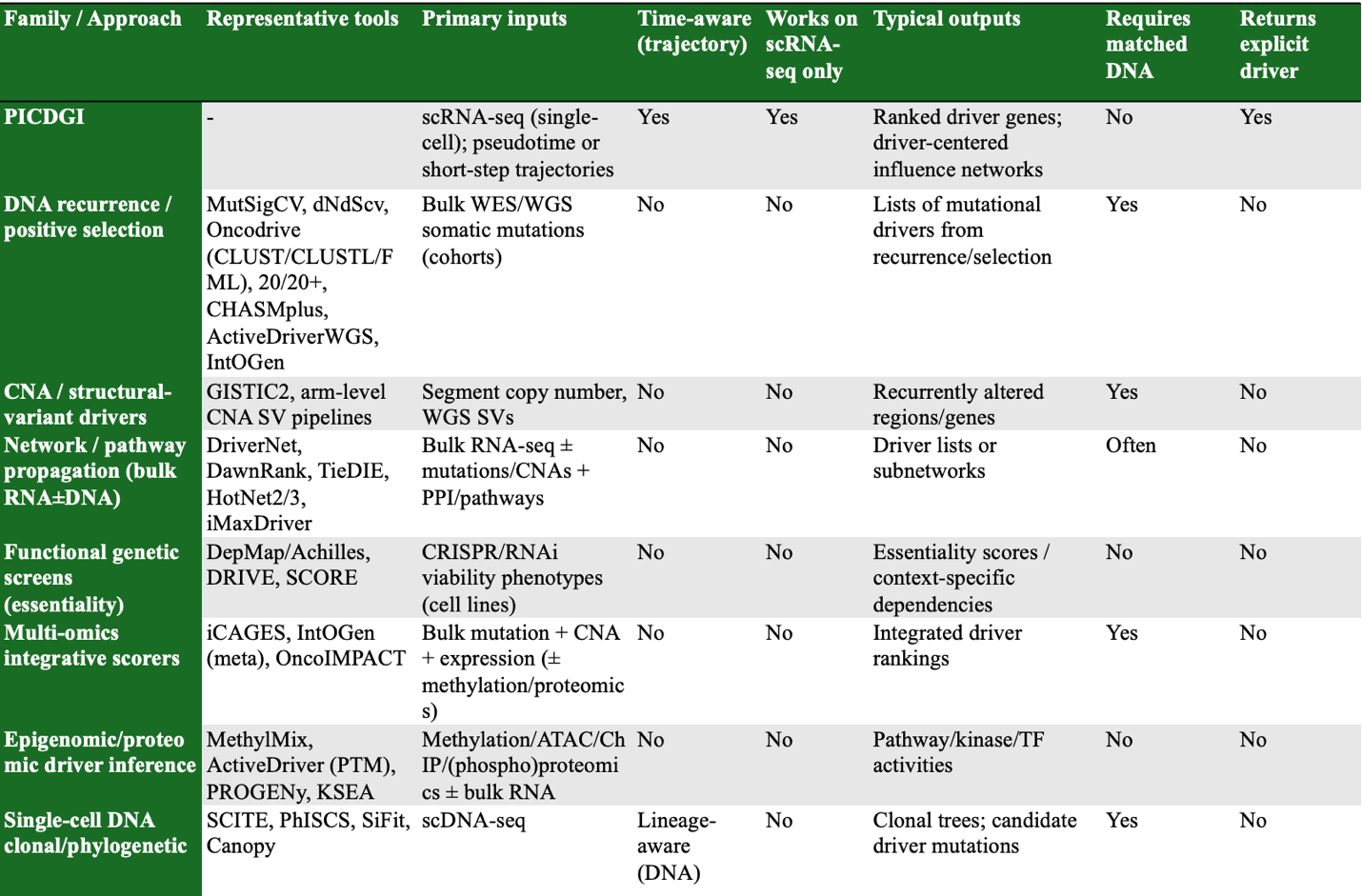

Supplement: S1 Table — (DOCX) [file pcbi.1014143.s013.docx]
