# Supplementary material for: PICDGI: A framework for predicting cancer driver genes through dynamic gene-gene interaction modeling of single-cell data
Source: PLoS Comput Biol. 2026 Apr 27;22(4):e1014143. doi: 10.1371/journal.pcbi.1014143 (PMC13119913; doi:10.1371/journal.pcbi.1014143)
Supplement: S7 Text — (DOCX) [file pcbi.1014143.s014.docx]

**S7 Text. Single-Cell RNA-Seq Data Acquisition, Preprocessing and Analysis Across Cancer Progression Stages for Individual Patients**

Single-cell RNA-seq (scRNA-seq) data used in this study were collected at three distinct time points from normal lung tissue, tumor lung tissue, and metastatic brain tissue, reflecting cancer progression stages [1]. Detailed experimental protocols for tissue dissociation and single-cell isolation are available in the original publication [1]. Briefly, single-cell suspensions were prepared from each tissue type and processed using the 10x Genomics Chromium platform for library construction, followed by sequencing on an Illumina NovaSeq system. Raw sequencing data were pre-processed using Cell Ranger (10x Genomics) for read alignment, feature quantification, and quality control. Cells with fewer than 200 detected genes or with mitochondrial gene expression exceeding 20% were excluded. Gene expression matrices were log-normalized and scaled prior to downstream analysis, ensuring robust and comparable single-cell transcriptomic profiles across samples.

**
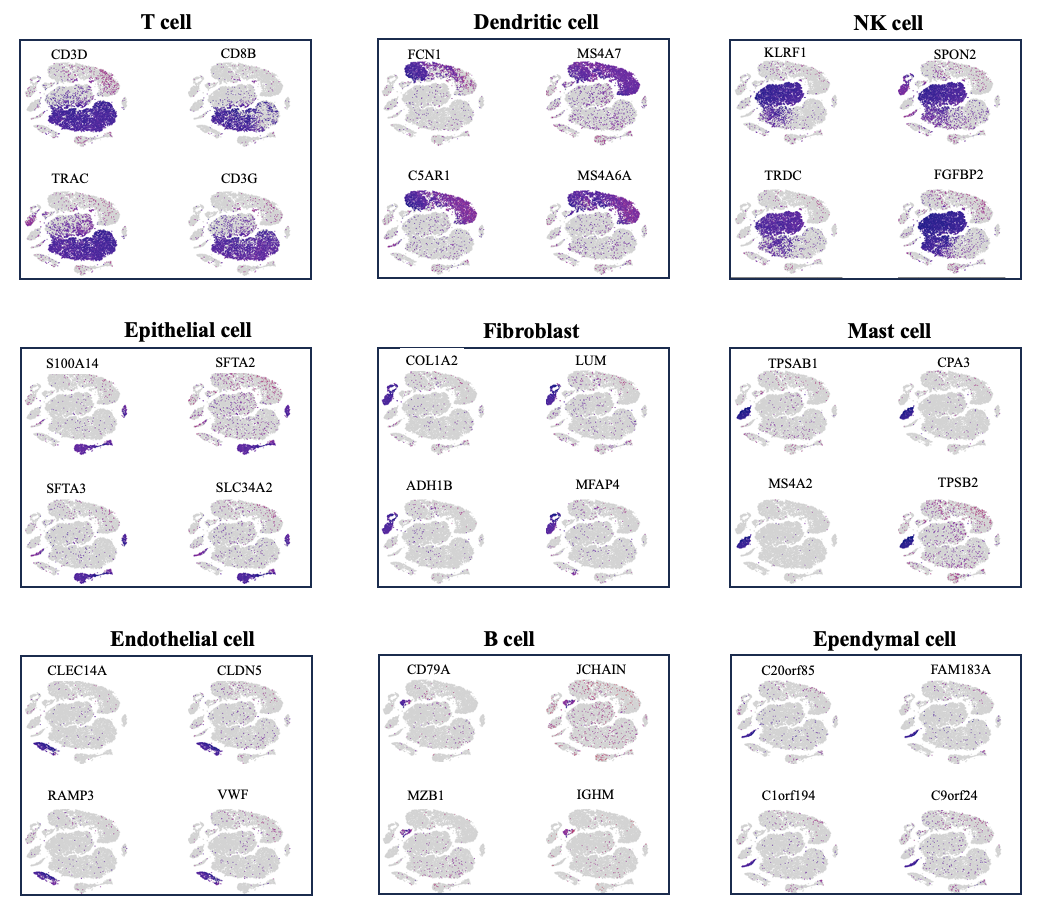
**

**S1 Fig. Single-Cell RNA-Seq Data Visualization for Patient1 at Early Stage.** tSNE plots for expression of the marker genes for the major cell lineages. Shown as markers for **T cells**: genes encoding the delta chain of the CD3 complex (CD3D), the beta chain of the CD8 protein (CD8B), T cell receptor alpha constant (TRAC), and the gamma chain of the CD3 antigen complex (CD3G). For **dendritic cells**, gene encoding ficolin-1 (FCN1), membrane-spanning 4-domains subfamily A member 7 (MS4A7), the complement component 5a receptor 1 (C5AR1), and the Membrane-Spanning 4-Domains Subfamily A Member 6A (MS4A6A). For **natural killer** cells, gene encoding Killer Cell Lectin-Like Receptor Subfamily F Member 1 (KLRF1), Spondin-2 (SPON2), T cell receptor delta constant region protein (TRDC), and Fibroblast Growth Factor-Binding Protein 2 (FGFBP2). For **epithelial cell**, gene encoding protein belonging to the S100 family of calcium-binding proteins (S100A14), Surfactant-Associated Protein A2 (SFTA2), Surfactant-Associated Protein A3 (SFTA3), and the sodium-dependent phosphate transporter protein (SLC34A2). For **Fibroblast**, gene encoding the alpha-2 chain of type I collagen (COL1A2), lumican (LUM), the beta subunit of alcohol dehydrogenase 1B (ADH1B), and the microfibril-associated glycoprotein 4 (MFAP4). For **Mast cells**, gene encoding the tryptase alpha/beta 1 (TPSAB1), carboxypeptidase A3 (CPA3), the membrane-spanning 4-domains subfamily A member 2 (MS4A2), and the tryptase beta-2 enzyme (TPSB2). For **endothelial cells**, gene encoding a protein known as C-type lectin domain family 14 member A (CLEC14A), and a protein called claudin-5 (CLDN5), the Receptor Activity Modifying Protein 3 (RAMP3), von Willebrand factor (VWF). For **B cells**, gene encoding the Ig-alpha chain (CD79A), the joining chain (JCHAIN), the Marginal zone B and B1 cell-specific protein (MZB1), and the mu constant region of the immunoglobulin heavy chain (IGHM). For **ependymal cells**, gene encoding the Chromosome 20 Open Reading Frame 85 (C20orf85), Family with sequence similarity 183 member A (FAM183A), Chromosome 1 Open Reading Frame 194 (C1orf194), and Chromosome 9 Open Reading Frame 24 (C9orf24).


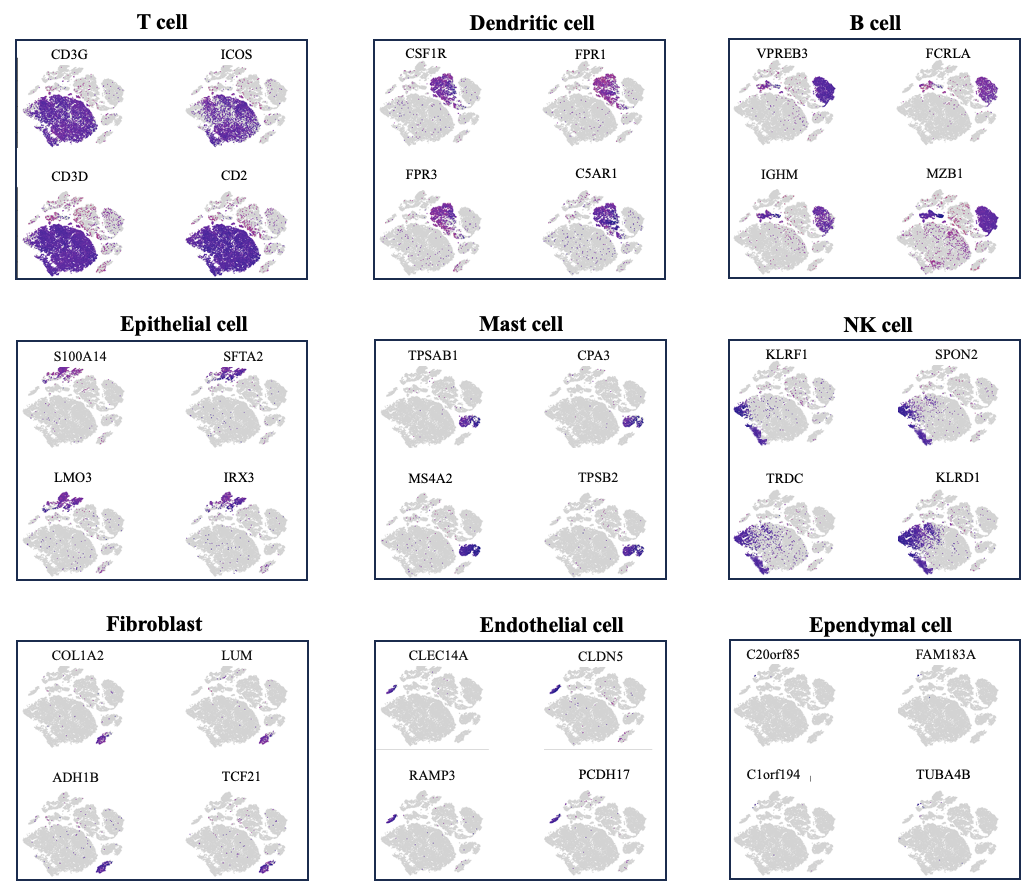


**S2 Fig. Single-Cell RNA-Seq Data Visualization for Patient1 for Patient1 at Mid Stage.** tSNE plots for expression of the marker genes for the major cell lineages. Shown as markers for **T cells**: genes encoding the gamma chain of the CD3 antigen complex (CD3G), Inducible T-cell Costimulator (ICOS), the delta chain of the CD3 complex (CD3D), and cell adhesion molecule (CD2). For **dendritic cells**, gene encoding Colony Stimulating Factor 1 Receptor (CSF1R), Formyl Peptide Receptor 1 (FPR1), Formyl Peptide Receptor 3 (FPR3), and complement component 5a receptor 1 (C5AR1). For **B cells**, gene encoding V-pre-B cell receptor 3 (VPREB3), Fc receptor-like A (FCRLA), the mu constant region of the immunoglobulin heavy chain (IGHM), and the Marginal zone B and B1 cell-specific protein (MZB1). For **epithelial cell**, gene encoding protein belonging to the S100 family of calcium-binding proteins (S100A14), Surfactant-Associated Protein A2 (SFTA2), LIM domain only protein 3 (LMO3), and Iroquois homeobox gene family (IRX3). For **Mast cells**, gene encoding the tryptase alpha/beta 1 (TPSAB1), carboxypeptidase A3 (CPA3), the membrane-spanning 4-domains subfamily A member 2 (MS4A2), the tryptase beta-2 enzyme (TPSB2). For **natural killer** cells, gene encoding Killer Cell Lectin-Like Receptor Subfamily F Member 1 (KLRF1), Spondin-2 (SPON2), T cell receptor delta constant region protein (TRDC), and a protein known as CD94 (KLRD1). For **Fibroblast**, gene encoding the alpha-2 chain of type I collagen (COL1A2), lumican (LUM), the beta subunit of alcohol dehydrogenase 1B (ADH1B), and the transcription factor 21 (TCF21). For **endothelial cells**, gene encoding a protein known as C-type lectin domain family 14 member A (CLEC14A), a protein called claudin-5 (CLDN5), the Receptor Activity Modifying Protein 3 (RAMP3), and protocadherin 17 (PCDH17). For **ependymal cells**, gene encoding Chromosome 20 Open Reading Frame 85 (C20orf85), Family with sequence similarity 183 member A (FAM183A), Chromosome 1 Open Reading Frame 194 (C1orf194), and tubulin alpha-4B chain (TUBA4B).


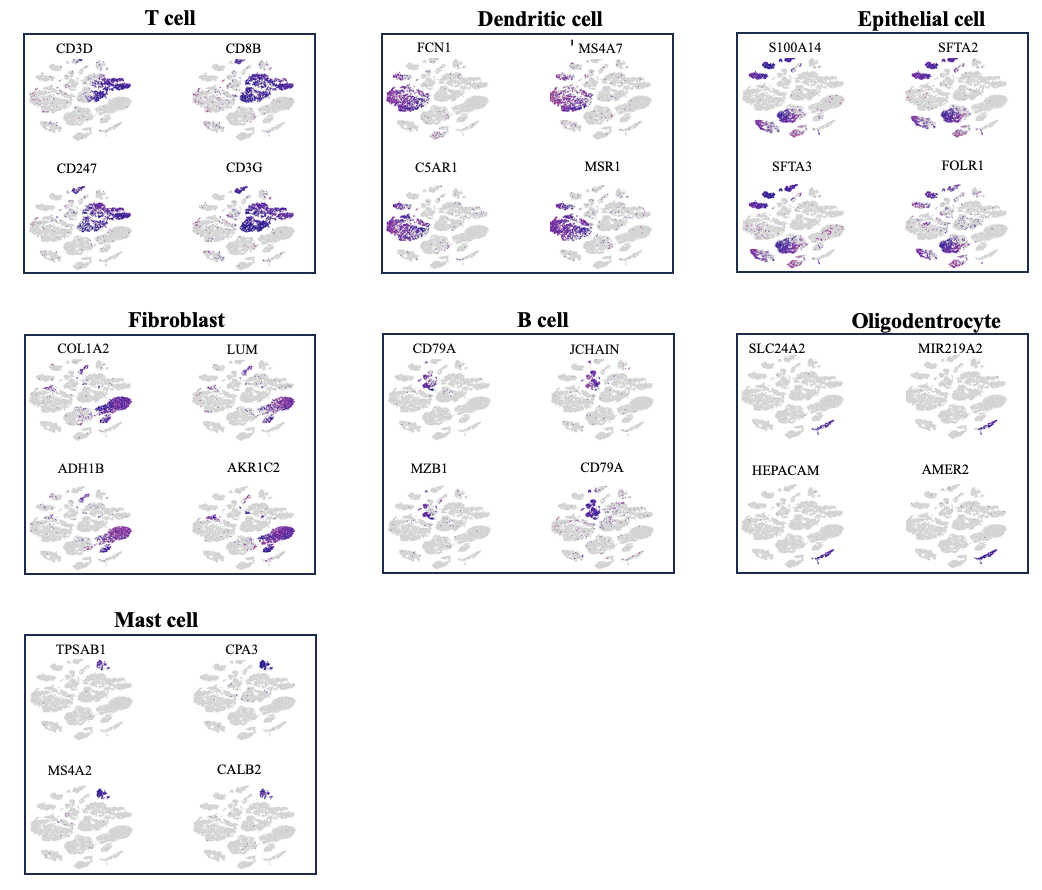


**S3 Fig. Single-Cell RNA-Seq Data Visualization for Patient1 at Late Stage.** tSNE plots for expression of the marker genes for the major cell lineages. Shown as markers for **T cells**: genes encoding the delta chain of the CD3 complex (CD3D), the beta chain of the CD8 protein (CD8B), a protein known as CD3 zeta chain (CD247), and the gamma chain of the CD3 antigen complex (CD3G). For **dendritic cells**, gene encoding ficolin-1 (FCN1), membrane-spanning 4-domains subfamily A member 7 (MS4A7), the complement component 5a receptor 1 (C5AR1), and the Macrophage Scavenger Receptor 1 (MSR1). For **epithelial cell**, gene encoding protein belonging to the S100 family of calcium-binding proteins (S100A14), Surfactant-Associated Protein A2 (SFTA2), Surfactant-Associated Protein A3 (SFTA3), and the Folate Receptor Alpha (FOLR1). For **Fibroblast**, gene encoding the alpha-2 chain of type I collagen (COL1A2), lumican (LUM), the beta subunit of alcohol dehydrogenase 1B (ADH1B), and the Aldo-Keto Reductase Family 1 Member C2 (AKR1C2). For **B cells**, gene encoding the Ig-alpha chain (CD79A), the joining chain (JCHAIN), the Marginal zone B and B1 cell-specific protein (MZB1), and a protein known as Ig-alpha (CD79A). For **Oligodentrocyte**, gene encoding solute carrier family 24 member 2 (SLC24A2), a microRNA called miR-219-2 (MIR219A2), the Hepatocyte Cell Adhesion Molecule (HEPACAM), and the Adenomatous polyposis coli membrane recruitment protein 2 (AMER2). For **Mast cells**, gene encoding the tryptase alpha/beta 1 (TPSAB1), carboxypeptidase A3 (CPA3), the membrane-spanning 4-domains subfamily A member 2 (MS4A2), and the Calretinin (CALB2).


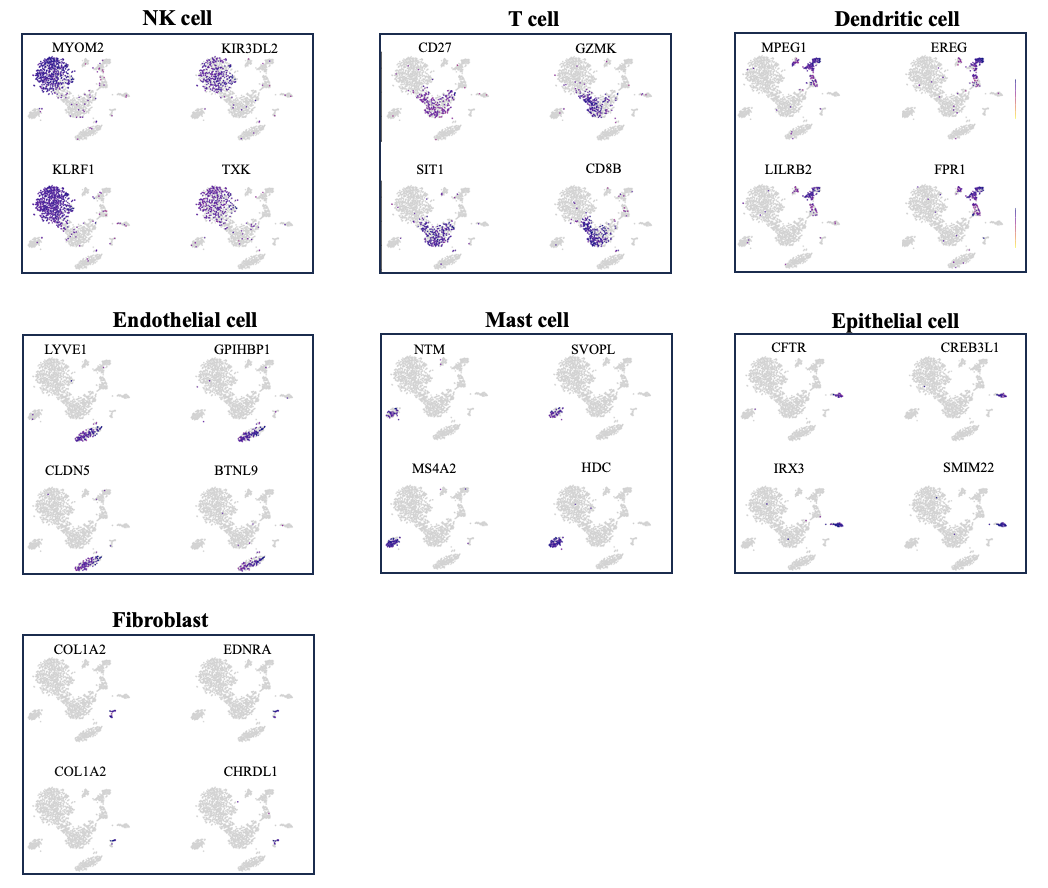


**S4 Fig. Single-Cell RNA-Seq Data Visualization for Patient2 at Early Stage.** tSNE plots for expression of the marker genes for the major cell lineages. Shown as markers for **natural killer** cells, gene encoding Myomesin-2 (MYOM2), Killer cell immunoglobulin-like receptor 3DL2 (KIR3DL2), Killer cell lectin-like receptor subfamily F member 1 (KLRF1), and T-cell-specific kinase (TSK). For **T cells**, gene encoding a protein known as (CD27), the Granzyme K (GZMK), the Suppressor of T-cell receptor Signaling 1 (SIT1), and CD8 beta chain (CD8B). For **dendritic cells**, gene encoding the macrophage-expressed gene 1 protein (MPEG1), Epiregulin (EREG), the Leukocyte Immunoglobulin Like Receptor B2 (LILRB2), and the Formyl Peptide Receptor 1 (FPR1). For **endothelial cells**, gene encoding Lymphatic Vessel Endothelial Hyaluronan Receptor 1 (LYVE1), The glycosylphosphatidylinositol-anchored high-density lipoprotein-binding protein 1 (GPIHBP1), the claudin-5 protein (CLDN5), and the butyrophilin-like (BTNL9). For **Mast cells**, gene encoding the neurotrimin (NTM), supervillin-like (SVOPL), membrane-spanning 4-domains subfamily A member 2 (MS4A2), and the Histidine Decarboxylase (HDC). For **epithelial cell**, gene encoding the Cystic Fibrosis Transmembrane Conductance Regulator (CFTR), the cAMP responsive element binding protein 3 like 1 (CREB3L1), the Iroquois homeobox protein 3 (IRX3), and the Small Integral Membrane Protein 22 (SMIM22). For **Fibroblast**, gene encoding the Collagen Type I Alpha 2 Chain (COL1A2), the Endothelin Receptor Type A (EDNRA), the alpha-2 chain of type I collagen (COL1A2), and Chordin Like 1 (CHRDL1).


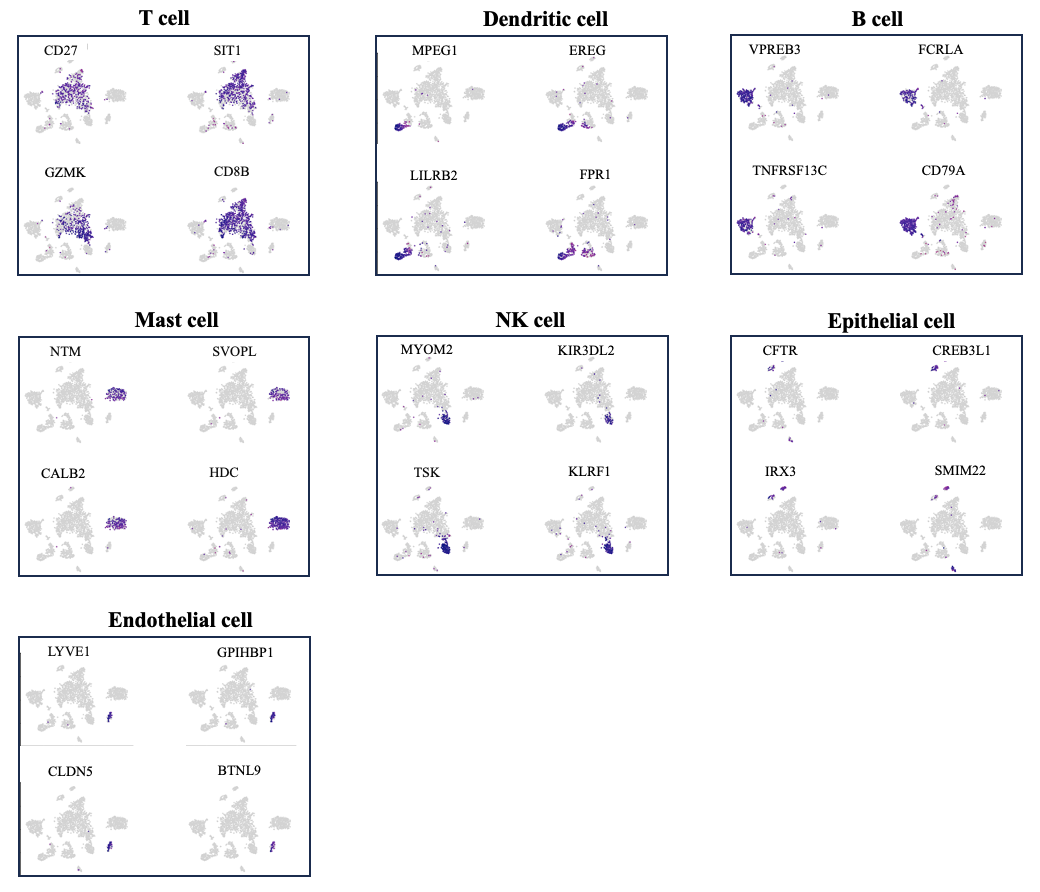


**S5 Fig. Single-Cell RNA-Seq Data Visualization for Patient2 at Mid Stage.** tSNE plots for expression of the marker genes for the major cell lineages. Shown as markers for **T cells**, gene encoding a protein known as (CD27), the Granzyme K (GZMK), the Suppressor of T-cell receptor Signaling 1 (SIT1), and CD8 beta chain (CD8B). For **dendritic cells**, gene encoding the macrophage-expressed gene 1 protein (MPEG1), Epiregulin (EREG), the Leukocyte Immunoglobulin Like Receptor B2 (LILRB2), and the Formyl Peptide Receptor 1 (FPR1). For **B cells**, gene encoding V-pre-B lymphocyte gene 3 (VPREB3), the Fc receptor-like A (FCRLA), the Tumor Necrosis Factor Receptor Superfamily Member 13C (TNFRSF13C), and the B-cell antigen receptor complex-associated protein alpha chain (CD79A). For **Mast cells**, gene encoding the neurotrimin (NTM), supervillin-like (SVOPL), the Calbindin 2 (CALB2), and the Histidine Decarboxylase (HDC). For **natural killer** cells, gene encoding Myomesin-2 (MYOM2), Killer cell immunoglobulin-like receptor 3DL2 (KIR3DL2), T-cell-specific kinase (TSK), and Killer cell lectin-like receptor subfamily F member 1 (KLRF1). For **epithelial cell**, gene encoding the Cystic Fibrosis Transmembrane Conductance Regulator (CFTR), the cAMP responsive element binding protein 3 like 1 (CREB3L1), the Iroquois homeobox protein 3 (IRX3), and the Small Integral Membrane Protein 22 (SMIM22). For **endothelial cells**, gene encoding Lymphatic Vessel Endothelial Hyaluronan Receptor 1 (LYVE1), The glycosylphosphatidylinositol-anchored high-density lipoprotein-binding protein 1 (GPIHBP1), the claudin-5 protein (CLDN5), and the butyrophilin-like (BTNL9).


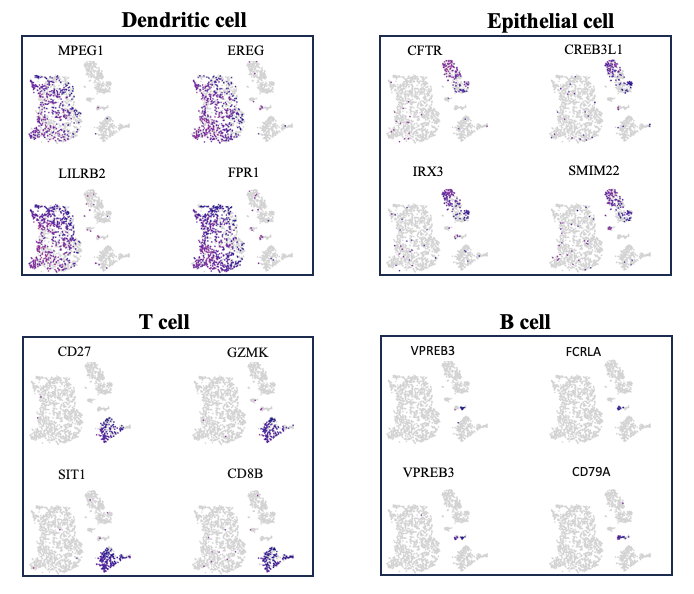


**S6 Fig. Single-Cell RNA-Seq Data Visualization for Patient2 at Late Stage.** tSNE plots for expression of the marker genes for the major cell lineages. Shown as markers for **dendritic cells**, gene encoding the macrophage-expressed gene 1 protein (MPEG1), Epiregulin (EREG), the Leukocyte Immunoglobulin Like Receptor B2 (LILRB2), and the Formyl Peptide Receptor 1 (FPR1). For **epithelial cells**, gene encoding the Cystic Fibrosis Transmembrane Conductance Regulator (CFTR), the cAMP responsive element binding protein 3 like 1 (CREB3L1), the Iroquois homeobox protein 3 (IRX3), and the Small Integral Membrane Protein 22 (SMIM22). For **T cells**, gene encoding a protein known as (CD27), the Granzyme K (GZMK), the Suppressor of T-cell receptor Signaling 1 (SIT1), and CD8 beta chain (CD8B). For **B cells**, gene encoding V-pre-B lymphocyte gene 3 (VPREB3), the Fc receptor-like A (FCRLA), V B-lymphoid tyrosine kinase (BLK) and the B-cell antigen receptor complex-associated protein alpha chain (CD79A).


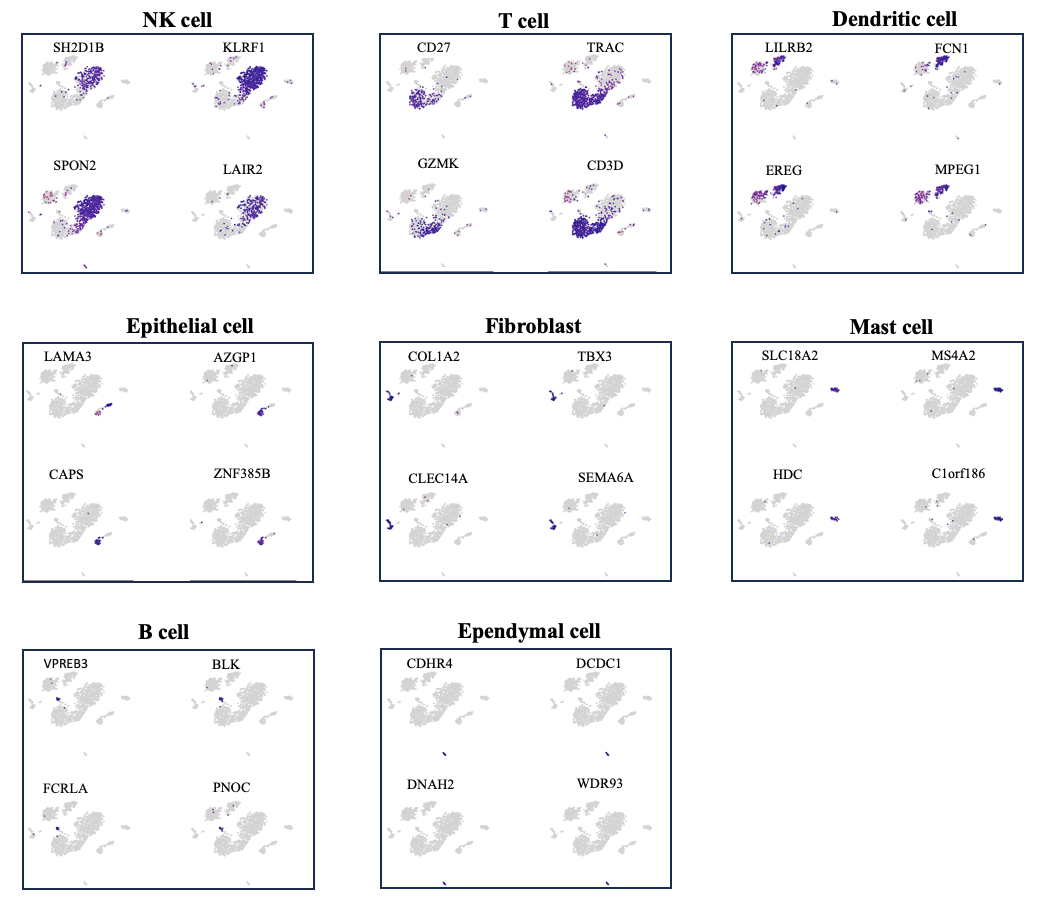


**S7 Fig. Single-Cell RNA-Seq Data Visualization for Patient3 at Early Stage.** tSNE plots for expression of the marker genes for the major cell lineages. Shown as markers for **natural killer** cells including, gene encoding the SH2 domain-containing protein 1B (SH2D1B), the Killer Cell Lectin Like Receptor F1 (KLRF1), the Spondin 2 (SPON2), and the Leukocyte-Associated Immunoglobulin-like Receptor 2 (LAIR2). For **T cells**, gene encoding a protein known as (CD27), the T-cell receptor alpha constant chain (TRAC), the Granzyme K (GZMK), and the CD3 complex (CD3D). For **dendritic cells**, gene encoding Leukocyte Immunoglobulin Like Receptor B2 (LILRB2), the Ficolin-1 (FCN1), the Epiregulin protein (EREG), and the Macrophage Expressed Gene 1 (MPEG1). For **epithelial cells**, gene encoding the laminin subunit alpha-3 (LAMA3), the Alpha-2-glycoprotein 1 (AZGP1), the Calcium-dependent activator protein for secretion (CAPS), and the Zinc Finger Protein 385B (ZNF385B). For **Fibroblast**, gene encoding the alpha-2 chain of type I collagen (COL1A2), the T-box transcription factor 3 (TBX3), the C-type lectin domain family 14 member A (CLEC14A), and the Semaphorin-6A (SEMA6A). For **Mast cells**, gene encoding a protein called Vesicular Monoamine Transporter 2 (SLC18A2), the membrane-spanning 4-domains subfamily A member 2 (MS4A2), the Histidine Decarboxylase (HDC), and the Chromosome 1 Open Reading Frame 186 (C1orf186). For **B cells**, gene encoding V-pre-B lymphocyte gene 3 (VPREB3), the Pre-B lymphocyte protein 3 (VPREB3), the Fc receptor-like A (FCRLA), and the prepronociceptin protein (PNOC). For **ependymal cells**, gene encoding the Cadherin-related family member 4 protein (CDHR4), the Doublecortin Domain Containing 1 (DCDC1), the Dynein Axonemal Heavy Chain 2 (DNAH2), and the WD repeat-containing protein 93 (WDR93).


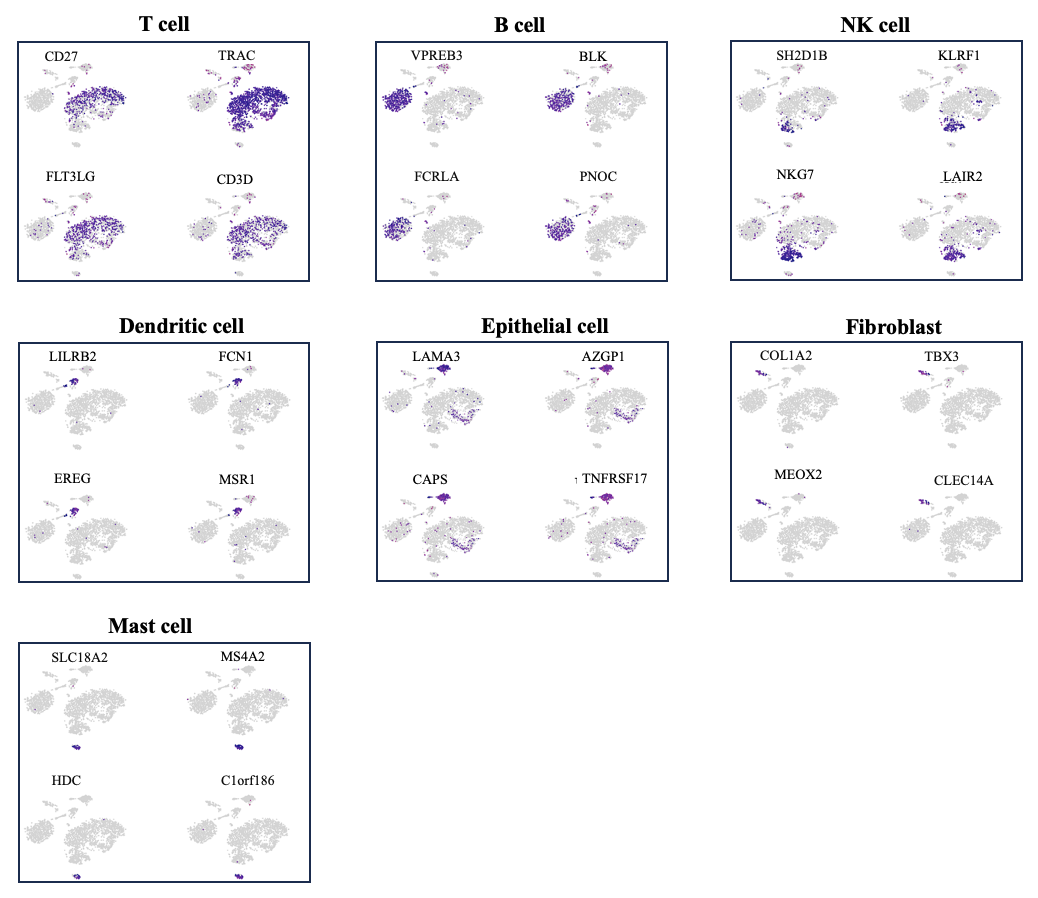


**S8 Fig. Single-Cell RNA-Seq Cell type Visualization for Patient3 at Mid Stage.** tSNE plots of immune and non-immune cells highlighting major marker genes and cell lineages. Markers for **T cells** include; gene encoding a protein known as (CD27), the T-cell receptor alpha constant chain (TRAC), Fms-related tyrosine kinase 3 ligand (FLT3LG), and the CD3 complex (CD3D). For **B cells**, gene encoding V-pre-B lymphocyte gene 3 (VPREB3), the B-lymphoid tyrosine kinase (BLK), the Fc receptor-like A (FCRLA), and the prepronociceptin protein (PNOC). For **natural killer** cells, gene encoding the SH2 domain-containing protein 1B (SH2D1B), the Killer Cell Lectin Like Receptor F1 (KLRF1), Natural Killer Cell Granule Protein 7 (NKG7), and the Leukocyte-Associated Immunoglobulin-like Receptor 2 (LAIR2). For **dendritic cells**, gene encoding Leukocyte Immunoglobulin Like Receptor B2 (LILRB2), the Ficolin-1 (FCN1), the Epiregulin protein (EREG), and the Macrophage Scavenger Receptor 1 protein (MSR1). For **epithelial cells**, gene encoding the laminin subunit alpha-3 (LAMA3), the Alpha-2-glycoprotein 1 (AZGP1), the Calcium-dependent activator protein for secretion (CAPS), and the Tumor Necrosis Factor Receptor Superfamily Member 17. For **Fibroblast**, gene encoding the alpha-2 chain of type I collagen (COL1A2), the T-box transcription factor 3 (TBX3), the mesenchyme homeobox 2 (Meox2), and the C-type lectin domain family 14 member A (CLEC14A). For **Mast cells**, gene encoding a protein called Vesicular Monoamine Transporter 2 (SLC18A2), the membrane-spanning 4-domains subfamily A member 2 (MS4A2), the Histidine Decarboxylase (HDC), and the Chromosome 1 Open Reading Frame 186 (C1orf186).


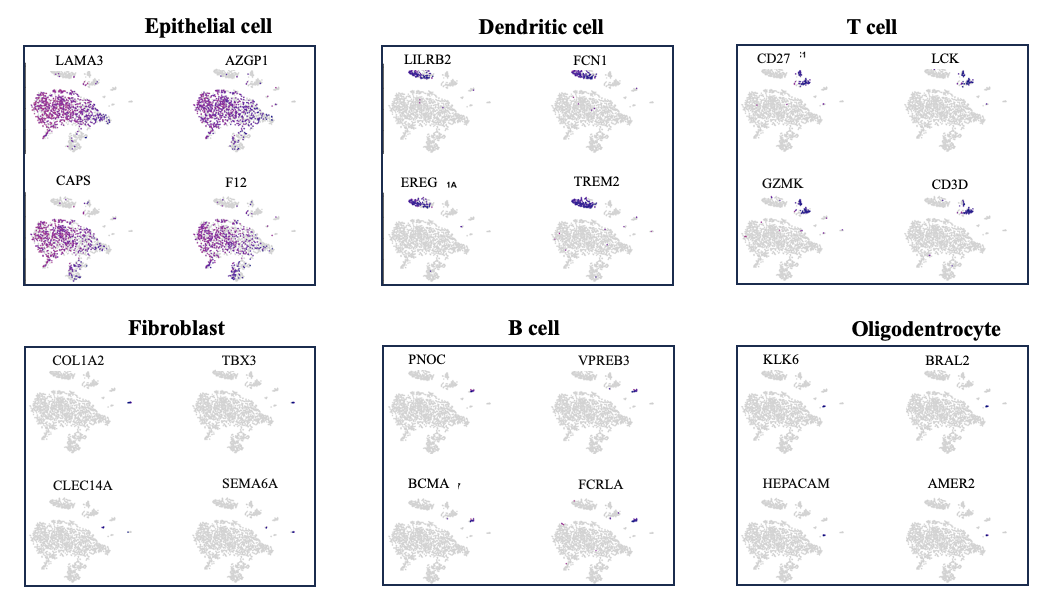


**S9 Fig. Single-Cell RNA-Seq Data Visualization for Patient3 at Late Stage.** tSNE plots for expression of the marker genes for the major cell lineages. Markers for **epithelial cells** include; gene encoding the laminin subunit alpha-3 (LAMA3), the Alpha-2-glycoprotein 1 (AZGP1), the Calcium-dependent activator protein for secretion (CAPS), and the coagulation factor XII (F12). For **dendritic cells**, gene encoding Leukocyte Immunoglobulin Like Receptor B2 (LILRB2), the Ficolin-1 (FCN1), the Epiregulin protein (EREG), and the Triggering Receptor Expressed on Myeloid cells 2 protein (TREM2). For **T cells**, gene encoding a protein known as (CD27), the lymphocyte-specific protein tyrosine kinase (LCK), the Granzyme K (GZMK), and the CD3 complex (CD3D). For **Fibroblast**, gene encoding the alpha-2 chain of type I collagen (COL1A2), the T-box transcription factor 3 (TBX3), the C-type lectin domain family 14 member A (CLEC14A), and the Semaphorin-6A (SEMA6A). For **B cells**, gene encoding the prepronociceptin protein (PNOC), the V-pre-B lymphocyte gene 3 (VPREB3), the B-cell Maturation Antigen (BCMA), and the Fc receptor-like A (FCRLA). For **Oligodentrocyte**, gene encoding kallikrein-related peptidase 6 (KLK6), brain-specific link protein (BRAL2), the Hepatocyte Cell Adhesion Molecule (HEPACAM), and the Adenomatous polyposis coli membrane recruitment protein 2 (AMER2).

**
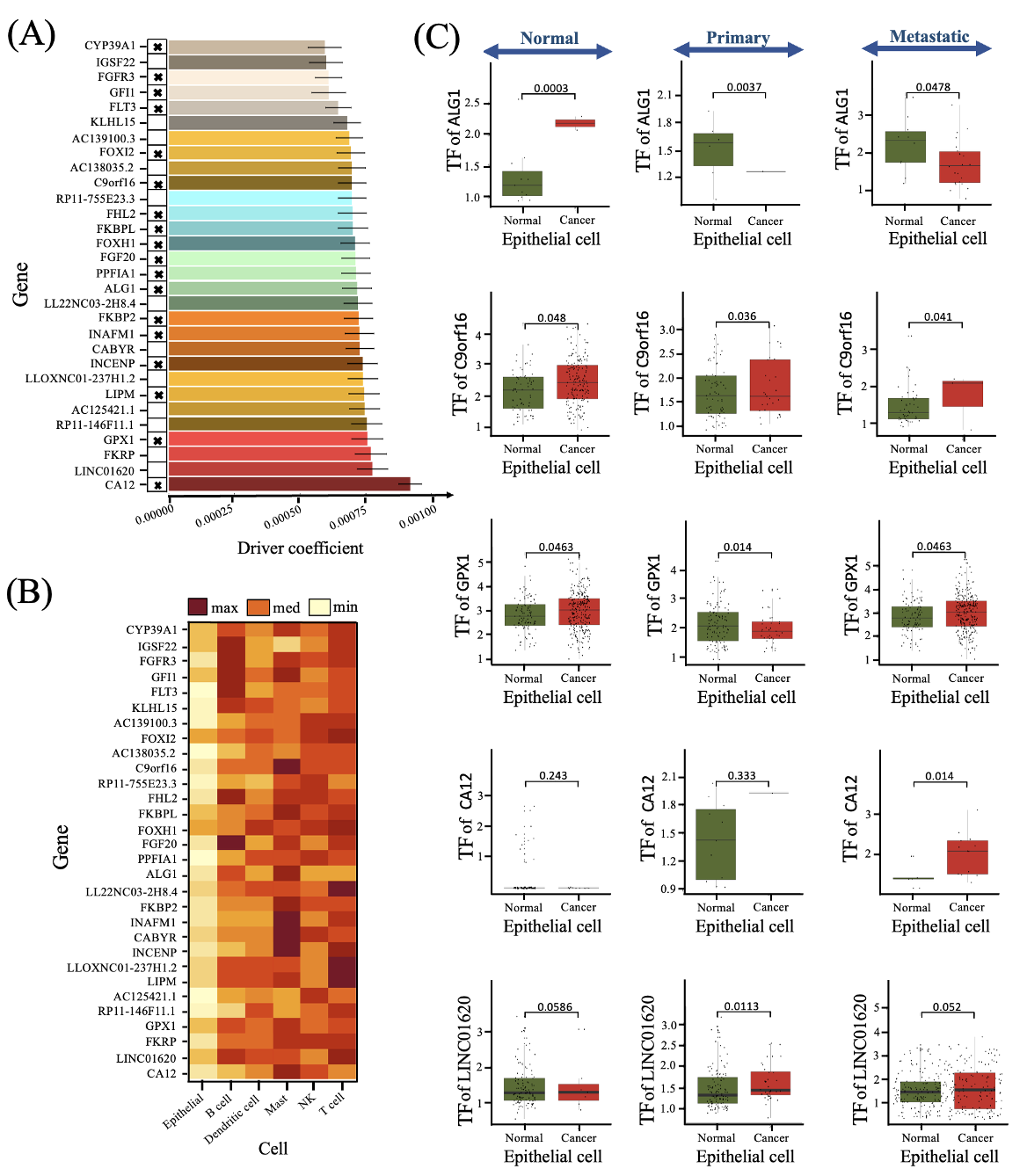
**

**S10 Fig. Cancer driver genes with the highest driver coefficients for Patient 2**. (A) Barplot of the driver coefficient of epithelial cell genes evaluated from the temporal gene expression data of patient 2. Data are presented as mean values +/- SEM (Standard Error of the Mean). The black cross mark in front of genes indicates previously identified oncogenes or tumor suppressor genes. (B) Heatmap depicting the degree of influence of genes driving LUAD in various cell types, annotated from single-cell data obtained from tissues of patient 1. (C) Boxplots displaying transcription factor (TF) activity and TF expression between normal epithelial and cancer cells for two representative TFs where there is substantial discordance between differential activity and differential expression. P-values for differential TF activity and TF expression derived from a t-test and a Wilcoxon rank-sum test, respectively. In boxplot, horizontal lines describe median, interquartile range, and whiskers extend to 1.5 × interquartile range.

**
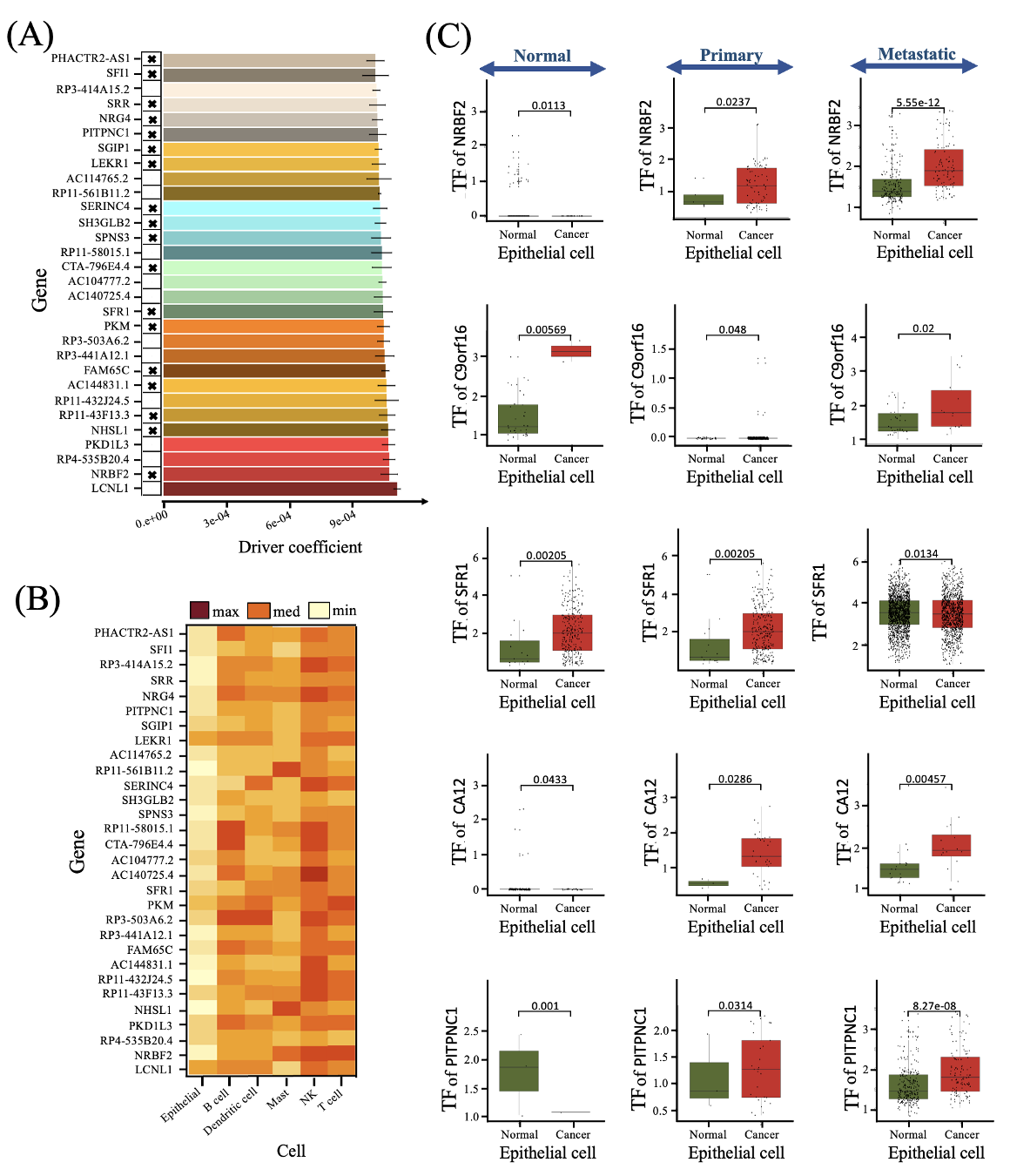
**

**S11 Fig. Cancer driver genes with the highest driver coefficients for Patient 3**. (A) Barplot of the driver coefficient of epithelial cell genes evaluated from the temporal gene expression data of patient 3. Data are presented as mean values +/- SEM (Standard Error of the Mean). The black cross mark in front of genes indicates previously identified oncogenes or tumor suppressor genes. (B) Heatmap depicting the degree of influence of genes driving LUAD in various cell types, annotated from single-cell data obtained from tissues of patient 1. (C) Boxplots displaying transcription factor (TF) activity and TF expression between normal epithelial and cancer cells for two representative TFs where there is substantial discordance between differential activity and differential expression. P-values for differential TF activity and TF expression derived from a t-test and a Wilcoxon rank-sum test, respectively. In boxplot, horizontal lines describe median, interquartile range, and whiskers extend to 1.5 × interquartile range.

*
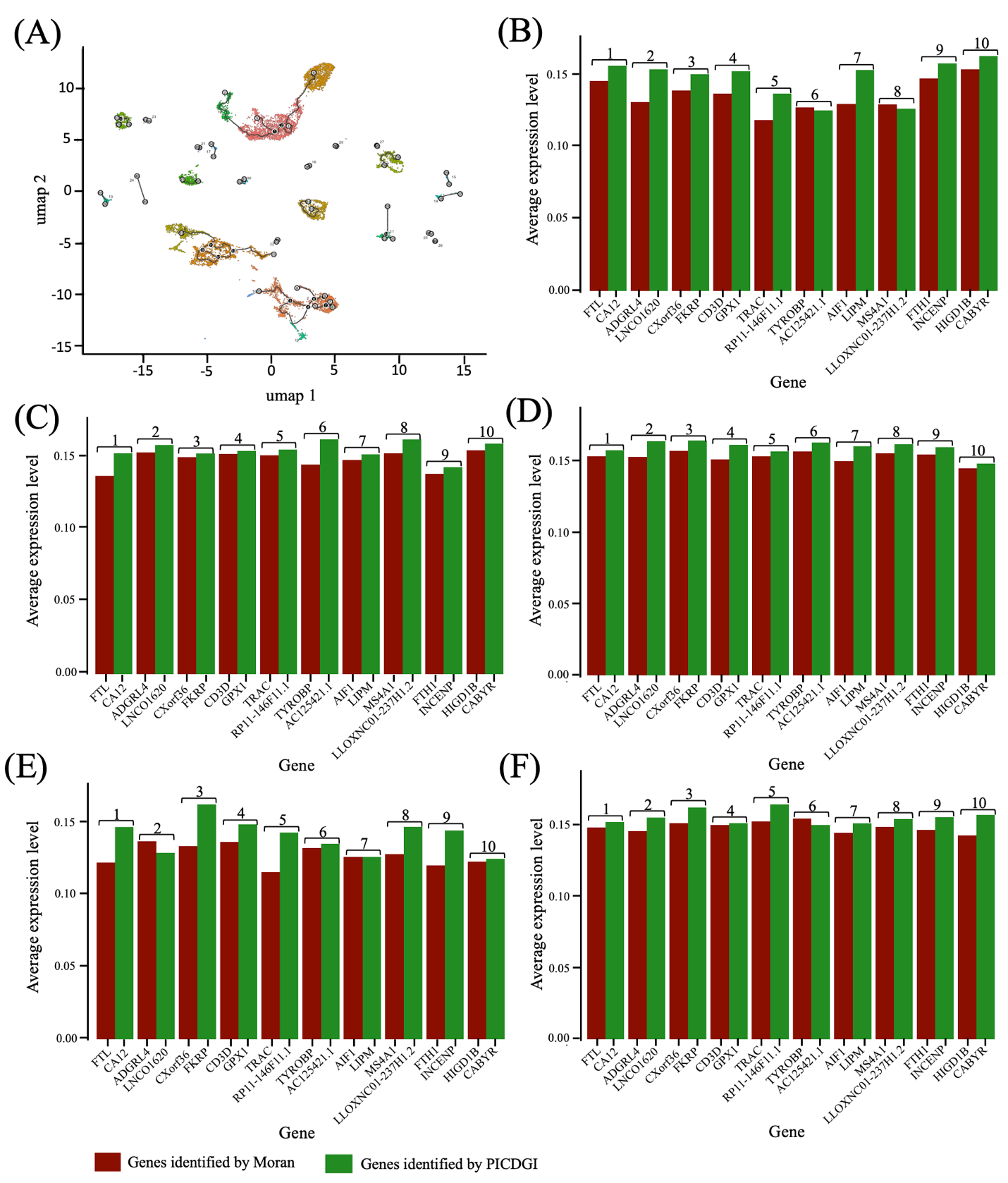
*

**S12 Fig. Comparison of PICDGI and Moran’s I Test for Driver Gene Prediction in Immune Cells for Patient 2.** (A) Single-cell 2D umap projection representing trajectory and pseudo-time values of cellular progression. (B-F) Comparisons of the PICDG framework with the existing Moran’s I test algorithm for predicting driver genes' inference in various immune cells. The driver genes identified through Moran’s I test display a lower average expression level compared to the expression level of driver genes presented by the PICDG computational framework. The genes are ranked from the highest to the lowest immune-suppressive role (1 to 10) for (B) Mast cells; (C) Natural Killers; (D) T cells; (E) B cells; (F) Dendritic cells.


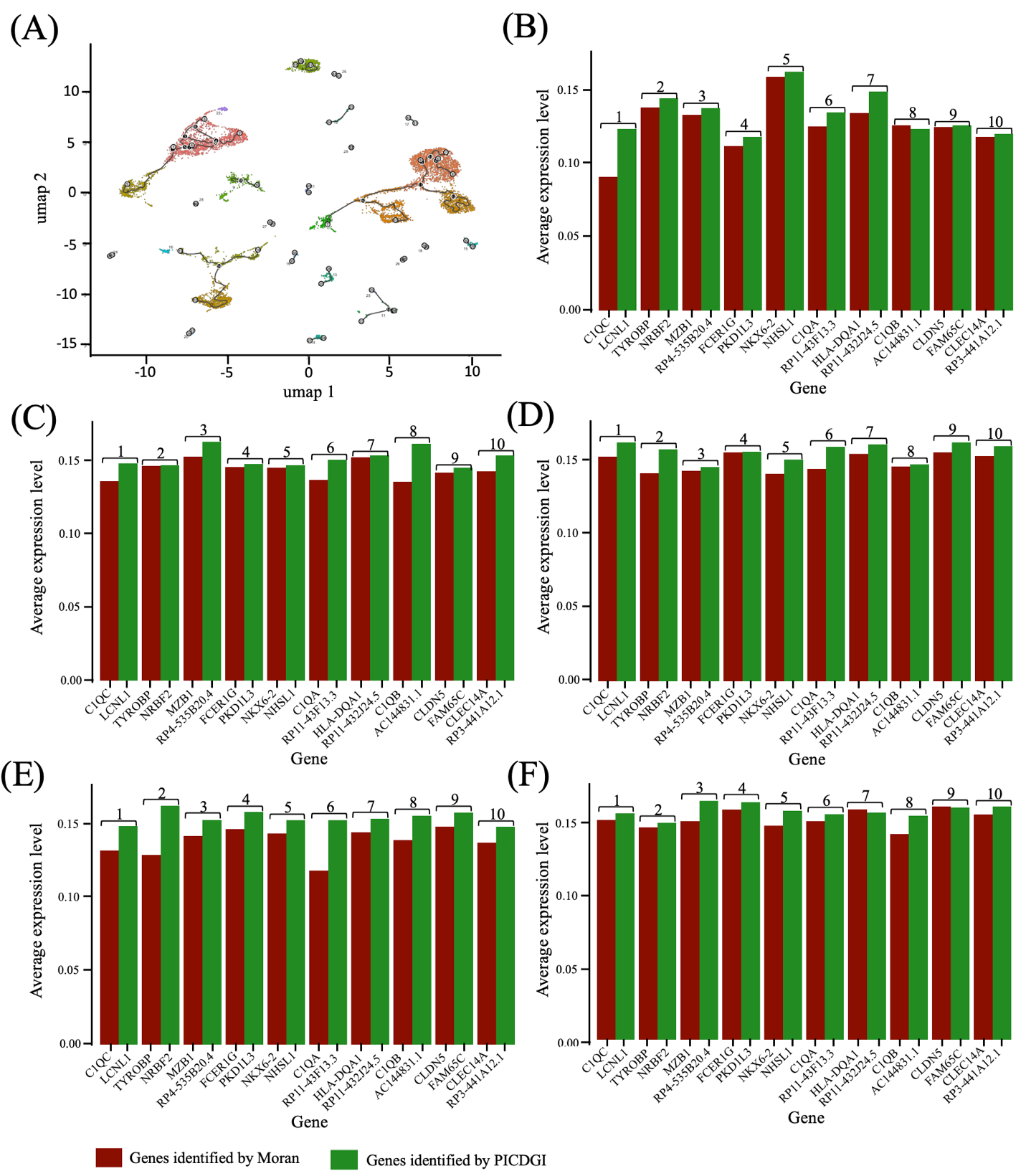
**S13 Fig. Comparison of PICDGI and Moran’s I Test for Driver Gene Prediction in Immune Cells for Patient 3.** (A) Single-cell 2D umap projection representing trajectory and pseudo-time values of cellular progression. (B-F) Comparing the PICDG framework with the existing Moran’s I test algorithm for predicting driver genes' inference in immune cells. The driver genes identified through Moran’s I test display a lower average expression level compared to the expression level of driver genes presented by the PICDG computational framework. The genes are ranked from the highest to the lowest immune-suppressive role (1 to 10) for; (B) Mast cell; (C) Natural Killer; (D) T cell; (E) B cell; (F) Dendritic cell.

**References**

1. Kim N, Kim HK, Lee K, Hong Y, Cho JH, Choi JW, et al. Single-cell RNA sequencing demonstrates the molecular and cellular reprogramming of metastatic lung adenocarcinoma. Nature communications. 2020;11(1):2285.
