# Supplementary material for: PICDGI: A framework for predicting cancer driver genes through dynamic gene-gene interaction modeling of single-cell data
Source: PLoS Comput Biol. 2026 Apr 27;22(4):e1014143. doi: 10.1371/journal.pcbi.1014143 (PMC13119913; doi:10.1371/journal.pcbi.1014143)
Supplement: S8 Text — (DOCX) [file pcbi.1014143.s028.docx]

**S8 Text. Ambient RNA Correction and Validation of Cancer Progenitor-Cell Identification**

To ensure that epithelial lineage markers were accurately represented in our single-cell datasets, we performed an additional analysis to account for ambient RNA contamination, a common artifact in droplet-based scRNA-seq data. Because enzymatic dissociation of LUAD tissues can cause ruptured cancer cells to release epithelial transcripts into the surrounding solution, we reprocessed all nine datasets using SoupX, a method that estimates and removes background RNA contributed by lysed cells. After ambient RNA correction, we reconstructed UMAP embeddings for Early, Mid, and Late stages across all three patients (Fig. 4A). These corrected embeddings revealed that EpCAM expression became highly specific to epithelial clusters, while immune and stromal cell populations displayed only background-level signal.

We then reassessed cell-type abundances across cancer progression (Fig. 4B). The corrected abundance profiles show that epithelial cells consistently expand from Early to Late stages for each patient, whereas other cell populations display heterogeneous or declining trends. This pattern aligns with established biological expectations for LUAD progression. Although we calculated EpCAM-based cancer cell fraction (CCF) for all annotated cell types during our analyses, only the epithelial-cluster CCF values were used in downstream modeling, including progenitor-cell identification and PICDGI driver-gene inference. Restricting CCF usage to epithelial cells prevents ambient RNA contamination from influencing any subsequent analytical step.

Altogether, this additional analysis confirms that epithelial cells robustly satisfy both lineage identity and stage-dependent expansion criteria and therefore constitute the most likely cancer progenitor population in the LUAD datasets examined. The ambient RNA-corrected results strengthen the reliability of progenitor-cell identification and ensure that downstream inference including dynamic modeling of interactions and driver gene discovery reflects true biological signal rather than technical artifacts.
