# Supplementary material for: PICDGI: A framework for predicting cancer driver genes through dynamic gene-gene interaction modeling of single-cell data
Source: PLoS Comput Biol. 2026 Apr 27;22(4):e1014143. doi: 10.1371/journal.pcbi.1014143 (PMC13119913; doi:10.1371/journal.pcbi.1014143)
Supplement: S9 Text — (DOCX) [file pcbi.1014143.s029.docx]

**S9 Text. Top 30 Cancer Driver Genes Identified by PICDGI Across All Three Patients**

**Patient 1**

1. **IGHGP**

The immunoglobulin heavy constant gamma P (IGHG1) gene encodes the gamma-1 chain of immunoglobulin G (IgG), which is a type of antibody involved in the immune response. The IGHG1 gene is located on chromosome 14 in humans and is part of the immunoglobulin gene cluster. It codes for the constant region of the gamma-1 chain, which determines the effector functions of IgG antibodies, including antibody-dependent cellular cytotoxicity (ADCC), complement activation, and opsonization. Variations or mutations in the IGHG1 gene can impact the structure and function of IgG antibodies, potentially affecting immune responses and susceptibility to infections or autoimmune diseases. Additionally, dysregulation of IgG production or function has been implicated in various immunological disorders and malignancies. Overall, the IGHG1 gene and its encoded product, the IgG gamma-1 chain, are essential components of the immune system, contributing to host defense and immune regulation[1].

1. **E2F5**

E2F5 Gene – E2F Transcription Factor 5. The protein encoded by this gene is a member of the E2F family of transcription factors. E2F5, belonging to the E2F family transcription factors, plays critical roles in multiple physiological and pathological processes, especially in carcinogenesis. Additionally, aberrant upregulation of E2F5 has been confirmed in various cancers involving tissues such as the breast, liver, colon, esophagus, prostate, ovary, and the stomach. E2F5 also serves as an independent indicator of advanced stages and poor prognosis in not only breast cancer and ovarian cancer but also in esophageal squamous cell carcinoma. Given that E2F5 is the target for several tumor-suppressive miRNAs, its roles in promoting the proliferation and invasion of cancer cells as well as mediating resistance to therapy have been well studied by not only modulating the cell cycle but also the Hippo, and Wnt pathways which play crucial roles in maintaining tissue homeostasis and organ size by orchestrating cell proliferation, differentiation and apoptosis. Although the pro-tumor functions of E2F5 are well known, its transcriptional targets, which could mediate the oncogenic roles of E2F5, are yet to be elucidated. Notably, a recent study reported that TFPI2, MMP-2, and MMP-9, which were transcriptionally regulated by E2F5, could mediate the oncogenic functions of E2F5 in prostate cancer. Nevertheless, more downstream targets of E2F5 are unknown in cancers[2].

1. **RP3-466P17.2**

RP3-466P17.2 has been identified as promising biomarker for lung cancer diagnosis and progression prediction**.** based on lncRNA microarray and polymerase chain reaction (PCR) validation ([57](https://www.ncbi.nlm.nih.gov/pmc/articles/PMC10151018/#B57)), suggesting that lncRNAs derived from TEPs can be used in the diagnosis and prediction of cancer progression[3].

1. **DCTD**

The DCTD gene, also known as deoxycytidine kinase, is responsible for encoding the enzyme deoxycytidine kinase. This enzyme plays a crucial role in nucleotide metabolism by catalyzing the phosphorylation of deoxycytidine (dC) and other deoxynucleosides to their corresponding nucleotide forms. These phosphorylated nucleotides are essential building blocks for DNA synthesis and repair. Mutations or dysregulation of the DCTD gene can impact nucleotide metabolism and contribute to various diseases, including cancer. Alterations in deoxycytidine kinase activity can affect the efficacy of nucleoside analog chemotherapy drugs, which rely on cellular kinases for activation. Therefore, understanding the function and regulation of the DCTD gene is important for cancer treatment and drug development. Hu et al. revealed *DCTD* as an efficient prognostic factor for malignant glioma. As DCTD inhibitor gemcitabine has been proposed as an adjuvant therapy for malignant glioma, their finding also suggests a therapeutic value of gemcitabine for the patients with high expression level of *DCTD*. Glioma is the most common primary intracranial tumor, accounting for 46% of all intracranial tumors, and 2% of all adult cancers[4].

1. **IGHV1OR16-3**

Immunoglobulin Heavy Variable 1/OR16-3 (Pseudogene). The gene IGHV1OR16-3 belongs to the immunoglobulin heavy chain variable (IGHV) gene family, specifically to the subgroup IGHV1. It plays a crucial role in the adaptive immune response by encoding part of the variable region of immunoglobulins, which are antibodies produced by B cells. No complete information regarding functionality of gene IGHV1OR16-3.

1. **TRIM63**

The expression of TRIM63 was significantly increased in breast cancer tissues and closely related to pathological differentiation and TNM stage of breast cancer. Overexpression of TRIM63 could significantly promote proliferation and migration of breast cancer cells, while TRIM63 knockdown significantly inhibited the proliferation and migration of breast cancer cells. In addition, TRIM63 could activate Wnt/β-catenin signaling pathway in breast cancer cells. Further study found that TRIM63 could regulate β-catenin degradation by promoting GSK3β phosphorylation. Li et al. revealed that TRIM63, as an oncogene, involved in breast cancer progression by activating the Wnt/β-catenin signaling pathway, suggesting that the potential applicability of TRIM63 as a target for breast cancer treatment[5].

1. **ANKRD20A4**

The gene ANKRD20A4, also known as Ankyrin Repeat Domain 20 Family Member A4, is a protein-coding gene located on chromosome 4 in humans. It belongs to the ankyrin repeat domain-containing protein family, which is characterized by the presence of multiple ankyrin repeat motifs involved in protein-protein interactions. While the specific function of ANKRD20A4 is not extensively studied, genes within the ankyrin repeat domain-containing protein family often play roles in various cellular processes. Its expression produces a protein of 823 amino acids with molecular mass of 94,149 Da. The protein family belong to membrane junction molecules that play an important role in linking intact membrane proteins to Spectrin-based cytoskeletal networks.

1. **RP11-799B12.2**

The gene RP11-799B12.2 is a type of long non-coding RNA (lncRNA) that is located on chromosome 11 in humans. It is a member of the RP11 gene family, which includes a large number of non-coding RNA genes that are often identified through genomic sequencing projects. As a non-coding RNA, RP11-799B12.2 does not encode a protein but instead plays regulatory roles in gene expression and other cellular processes. While the specific function of RP11-799B12.2 is not yet fully understood, lncRNAs like RP11-799B12.2 have been implicated in various biological processes, including chromatin remodeling, transcriptional regulation, and post-transcriptional gene regulation.

1. **FLNB**

It has been recently demonstrated that *FLNB* plays an important role in cancer. Several previous studies demonstrated that *FLNB* expression was highly correlated with tumor proliferation, metastasis and invasiveness. For example, a *FLNB* gene deficiency in mouse embryonic fibroblasts increased the expression and proteolytic activity of matrix metalloproteinase-9 (MMP-9), as well as cell invasion mediated by the RAS/ERK pathway. Another *in vitro* study demonstrated that *FLNB* is highly expressed in several cancer cells, such as A549 (adenocarcinomic human alveolar basal epithelial cells) and HT1080 (fibrosarcoma cell line) cells, which exhibit high invasiveness. In particular, an alternative splicing (AS) switch in *FLNB* promotes the epithelial-to-mesenchymal transition (EMT) in human breast cancer[6].

1. **SVEP1**

The SVEP1 gene encodes the Sushi, von Willebrand factor type A, EGF, and pentraxin domain-containing protein 1, also known as SVEP1. This protein is involved in various cellular processes, including cell adhesion, migration, and tissue development. SVEP1 contains multiple functional domains, including sushi domains, von Willebrand factor type A domains, epidermal growth factor (EGF)-like domains, and pentraxin domains. These domains are often found in proteins involved in cell-cell and cell-matrix interactions. SVEP1 mutation and its mRNA expression are related to tumor mutation burden and cancer immunity in lung squamous cell carcinoma. Luo et al.’s findings reveal the underlying mechanisms, indicating that SVEP1 may be a prognostic marker of lung squamous cell carcinoma[7]. `

1. **ALK**

ALK’s main functional role in lung adenocarcinoma is as an oncogenic driver tyrosine kinase whose rearrangements and overexpression drive tumor initiation, growth, survival, and progression, and influence prognosis and therapy response[8].

1. **ABCB10**

ABCB10 is located in the inner mitochondrial membrane forming homodimers, with the ATP binding domain facing the mitochondrial matrix. ABCB10 expression is highly induced during erythroid differentiation and its overexpression increases hemoglobin synthesis in erythroid cells. Recent studies have found that the abnormal expression of Circ-ABCB10, also known as hsa_circ_000871, may be involved in the occurrence and development of many different tumors, such as esophageal squamous carcinoma cells, glioma, non–small cell lung cancer, oral squamous cell carcinoma, lung cancer, epithelial ovarian cancer, breast cancer, thyroid cancer, and hepatocellular carcinoma (Cortés-López and Miura, 2016; Wang et al., 2017). Thus, it is significant to summarize the function of Circ-ABCB10 in the occurrence and progression of human cancers[9].

1. **JPH1**

Junctophilin-1 is a protein that in humans is encoded by the JPH1 gene. Junctional complexes between the plasma membrane and endoplasmic/sarcoplasmic reticulum are a common feature of all excitable cell types and mediate cross talk between cell surface and intracellular ion channels. The protein encoded by this gene is a component of junctional complexes and is composed of a C-terminal hydrophobic segment spanning the endoplasmic/sarcoplasmic reticulum membrane and a remaining cytoplasmic domain that shows specific affinity for the plasma membrane. This gene is a member of the junctophilin gene family. Junctophilin1 is responsible for the formation and stabilization of sarcoplasmic reticulum - plasma membrane (SR-PM) junctions in striated muscle and actively participate in the recruitment of the two essential players in intracellular calcium release, skeletal, and cardiac muscles and ryanodine receptor. The junctophilins1 plays an important role in the formation and organization of SR-PM junctions in skeletal and cardiac muscle and on the functional consequences of the absence or malfunction of these proteins in striated muscle in light of recently published data and recent advancements in protein structure prediction. JPH1 was also hypothesized to be a disease-modifier gene in individuals with CMT2K[9, 10].

1. **TPO**

The TPO gene encodes the enzyme thyroid peroxidase, also known as thyroperoxidase. This enzyme plays a crucial role in the synthesis of thyroid hormones by catalyzing the iodination of thyroglobulin and the coupling of iodotyrosine residues to form thyroxine (T4) and triiodothyronine (T3). **TPO may be useful in confirming or ruling out benign diseases from differentiated thyroid carcinoma,** with the exception of low-risk carcinoma such as MIFC. It could be used as a prognostic factor for differentiated thyroid cancer and patient follow-up, together with other markers. Functional enrichment analysis showed that TPO function was significantly associated with signaling pathways related to amino acid metabolism, gene expression regulation and tumorigenesis. TPO expression was also significantly associated with immune infiltration. Our study showed that reduced TPO expression was significantly associated with lymph node metastasis and recurrence in patients with PTC, and we validated this result in our central cohort. These data suggest that TPO may serve as a prognostic indicator for PTC[11].

1. **CHEK1**

The CHEK1 gene, also known as checkpoint kinase 1, encodes a protein that plays a key role in cell cycle regulation and DNA damage response. CHEK1 is majorly involved in the coordination of DNA repair and therefore is an area of great interest in cancer development and treatment. The CHEK1 gene was previously thought to function as a tumor suppressor because of the regulatory role it plays in DNA damage. However, no evidence of homozygous loss of function mutants for CHEK1 in human cancers was reported. Rather, this gene has been revealed to be overexpressed in several solid tumors. The correlation of CHEK1 expression with tumor grade and disease recurrence was also reported, suggesting its role in tumor development. Studies have shown that complete loss of CHEK1 suppresses chemically induced carcinogenesis, and its low expression may result in tumor progression[12].

1. **PABPC1**

The PABPC1 gene, also known as Polyadenylate-binding protein 1, encodes a protein that plays a crucial role in post-transcriptional regulation of gene expression. PABPC1 is involved in mRNA metabolism, specifically in the control of mRNA stability, translation, and localization. PABPC1 acts as an oncogene in hepatocellular carcinoma, accelerating cell proliferation and promoting anchorage-independent growth by promoting cell entry into S and G2/M phases. The specific mechanism is that PABPC1 interacts with AGO2 in the cytoplasm of hepatocellular carcinoma cells, and this interaction increases the recruitment of mRNA to RISC and represses multiple oncogenes. For miRNA-targeted genes, PABPC1 increases the efficiency of miRNA repression, and this efficiency is higher in cancer cells than in normal cells. For miRNA non-target genes, PABPC1 interacted with eIF4G to inhibit the decay of mRNA, making translation higher in cancer cells than in normal cells and thus increasing cellular activity[13].

1. **RP11-266N13.2**

The gene RP11-266N13.2 is a non-coding RNA (ncRNA) gene. Non-coding RNAs are RNA molecules that are not translated into proteins but play various regulatory roles in the cell. The specific function of RP11-266N13.2 is not well characterized, as it belongs to a class of genes that are still being studied to understand their biological significance. No complete information regarding functionality of gene RP11-266N13.2.

1. **AC136352.5**

The gene AC136352.5 is a long non-coding RNA (lncRNA) gene. Long non-coding RNAs are a class of RNA molecules that do not encode proteins but play important regulatory roles in various cellular processes. The specific function of AC136352.5 is not well characterized, as it belongs to a class of genes that are still being studied to understand their biological significance. No complete information regarding functionality of gene AC136352.5.

1. **RP11-290D2.3**

The gene RP11-290D2.3 is a long non-coding RNA (lncRNA) gene. Long non-coding RNAs are a class of RNA molecules that do not encode proteins but play important regulatory roles in various cellular processes. No complete information regarding functionality of gene RP11−290D2.3.

1. **HnRNP**

The term "HnRNP" typically refers to a family of proteins known as heterogeneous nuclear ribonucleoproteins. These proteins play crucial roles in RNA metabolism, including pre-mRNA processing, mRNA transport, and mRNA stability. HnRNP contribute to the diversity of tumor and immune-associated aberrant proteomes by controlling alternative splicing and translation. They can also promote cancer-associated gene expression by regulating transcription factors, binding to DNA directly, or promoting chromatin remodeling. HnRNP are emerging as newly recognized mRNA readers. Here, we review the roles of hnRNP as regulators of the cancer-immune landscape. Dissecting the molecular functions of hnRNP will provide a better understanding of cancer-immune biology and will impact the development of new approaches to control and treat cancer. It has been implicated in multiple cancers, suggesting its role in tumourigenesis, but the potential oncogenic role and mechanism of hnRNP-F in bladder cancer (BC) remain incompletely understood[14, 15].

1. **MSH3**

The gene MSH3, also known as MutS Homolog 3, is involved in DNA mismatch repair (MMR), which is a crucial mechanism for maintaining genomic stability. MSH3 is a member of the MutS family of proteins, which play essential roles in recognizing and repairing DNA mismatches and small insertion-deletion loops that occur during DNA replication and recombination. The MSH3 gene is located at chromosome 5q11-13 and encodes a protein of 1137 amino acid residues [[9](https://www.ncbi.nlm.nih.gov/pmc/articles/PMC4637639/#b9)]. Some potentially functional single-nucleotide polymorphisms (SNPs) of MSH3 may have influence the DNA repair capacity and thereby predispose individuals to a variety of cancers[16].

1. **GRK6**

The gene GRK6, also known as G protein-coupled receptor kinase 6, encodes a member of the G protein-coupled receptor kinase family of proteins. These kinases play a crucial role in regulating the activity of G protein-coupled receptors (GPCRs), which are cell surface receptors involved in transmitting signals from outside the cell to the inside. Studies investigating the function of GRK6 in patients with hepatocellular carcinoma using immunohistochemistry have demonstrated a positive correlation between GRK6 and Ki-67 expression, pathological disease stage, metastasis, and survival rate. These authors hypothesized that GRK6 could be used as a biomarker for the early diagnosis of hepatocellular carcinoma [^134^](https://www.ncbi.nlm.nih.gov/pmc/articles/PMC5821040/#B134). Furthermore, recoverin, which is functionally associated with GRK6 (as well as GRK2 and GRK5), was aberrantly expressed in SSTW-2 gastric cancer cells[17].

1. **HSPA13**

The gene HSPA13, also known as heat shock protein family A member 13, encodes a member of the heat shock protein 70 (HSP70) family. Heat shock proteins are a group of molecular chaperones that play essential roles in protein folding, transport, and degradation, particularly under conditions of cellular stress.

Previous studies have reported upregulation of HSPA13 in HCC, but the underlying mechanism and phenotype have not been thoroughly investigated. Cen ant al.’s studies filled this gap and found that HSPA13 promotes the proliferation, migration, and invasion of HCC cells in vitro, and facilitates tumor growth in vivo. HSPA13 has been recognized as a proto-oncogene in various cancers, including colon cancer, breast cancer, and cutaneous melanoma. For example, in colon cancer, knockdown of HSPA13 accelerates apoptosis and necrosis, facilitating ubiquitination of RIP1 in response to TNFα induction. In addition to this, HSPA13 promotes invasion and metastasis of breast cancer cells by regulating angiogenesis and nutrient supply. Moreover, HSPA13 is a risk gene in cutaneous melanoma and is associated with poor patient prognosis. Collectively, these studies, along with our findings, support the notion that HSPA13 expression positively regulates tumorigenesis[18].

1. **APC**

The gene APC, or adenomatous polyposis coli, is a tumor suppressor gene located on chromosome 5q21-22. It plays a crucial role in regulating cell proliferation, differentiation, and adhesion, primarily through its involvement in the Wnt signaling pathway. Biallelic mutation of the APC gene occurs in 45%–80% of colorectal cancers and is observed in the earliest detectable lesions. The APC locus was originally identified based on its link to familial adenomatous polyposis coli (FAP), an inherited syndrome of cancer predisposition. Inherited mutations in the APC gene cause affected individuals to develop hundreds to thousands of adenomatous polyps, resulting in the onset of CRC typically before the age of 40. Individuals with FAP inherit a loss-of-function mutation in a single allele of APC, followed by an additional acquired mutation in the second allele of APC in the adenomas and adenocarcinomas that develop. Thus, the acquisition of biallelic APC mutations represents an early and rate-limiting step in all FAP-associated and most sporadic colorectal tumors[19].

1. **PDCD4-AS1**

The gene PDCD4-AS1, also known as programmed cell death 4 antisense RNA 1, is a long non-coding RNA (lncRNA) located on chromosome 10q24.1. lncRNAs are RNA molecules that are longer than 200 nucleotides and do not code for proteins but instead play regulatory roles in gene expression. Depletion of PDCD4-AS1 results in promotion of cell proliferation and migration in breast cancer cells. It is believed that PDCD4-AS1 stabilizes PDCD4 mRNA by forming an RNA duplex to attenuate the interaction between PDCD4 mRNA and RNA decay promoting factor HuR. Moreover, LncRNA GAS5 interacts with miR-21 via miRNA binding elements in breast cancer. Mediated by this sponge mechanism, GAS5 is involved in the upregulation of a number of mRNAs that encode tumor suppressor proteins such as PDCD4. A similar mechanism that lncRNA SLC16A1-AS1 may serve as an internal sponge of miR-182 to augment PDCD4 has been reported recently. SLC16A1-AS1 is downregulated in TNBC and overexpression of SLC16A1-AS1 could suppress TNBC cell proliferation[20].

1. **FBXO6**

The gene FBXO6, also known as F-box protein 6, is located on chromosome 2q31.2 and encodes a member of the F-box protein family. F-box proteins are components of the Skp1-Cullin-F-box (SCF) ubiquitin ligase complex, which plays a crucial role in protein degradation via the ubiquitin-proteasome pathway. Through analysis of the TCGA dataset, we found that FBXO6 was significantly increased in ovarian cancer tissues and the high expression of FBXO6 was related to the poor overall survival (OS) of ovarian cancer patients at advanced stages. An inverse correlation between the protein levels of FBXO6 and RNASET2 was observed in clinic ovarian cancer samples. Depletion of FBXO6 promoted ovarian cancer cells proliferation, migration, and invasion, which could be partially reversed by RNASET2 silencing. Thus, our data revealed a novel FBXO6-RNASET2 axis, which might contribute to the development of ovarian cancer. We propose that inhibition of FBXO6 might represent an effective therapeutic strategy for ovarian cancer treatment[21].

1. **ATP11AUN**

ATP11AUN gene is a unknown which located on 13q34. 20 organisms have orthologs with human gene ATP11AUN. No complete information regarding functionality of gene ATP11AUN!

### **VPS37D**

### VPS37D is a gene that encodes a protein called vacuolar protein sorting-associated protein 37D, which is involved in the sorting and transport of proteins within cells. It plays a role in the endosomal sorting complex required for transport (ESCRT) pathway, which is important for protein degradation and vesicle formation. Molecular details of how endocytosis contributes to oncogenesis remain elusive[22].

1. **CCNL2**

The CCNL2 gene encodes a protein called Cyclin L2, which is a member of the cyclin family involved in regulating the cell cycle. Cyclins interact with cyclin-dependent kinases (CDKs) to control progression through different phases of the cell cycle. CCNL2 is a novel member of the cyclin gene family. Studies have shown that it acts as a tumor suppressor protein in gastric cancer and lung cancer[23].

1. **TP53INP1**

The TP53INP1 gene, also known as Tumor Protein P53-Inducible Nuclear Protein 1, encodes a protein involved in various cellular processes, including apoptosis, autophagy, and cell cycle regulation. TP53INP1 (tumor protein 53-induced nuclear protein 1) is a tumor suppressor, whose expression is downregulated in cancers from different organs. It was described as a p53 target gene involved in cell death, cell-cycle arrest and cellular migration[24].

**Patient 2**

1. **CYP39A1**

The CYP39A1 gene encodes an enzyme called cytochrome P450 39A1, which belongs to the cytochrome P450 superfamily of enzymes. These enzymes are involved in the metabolism of various endogenous and exogenous compounds, including drugs, toxins, and hormones. Specifically, CYP39A1 is responsible for catalyzing the hydroxylation of various substrates, including steroids and fatty acids. Downregulation of CYP39A1 is associated with HCC carcinogenesis, tumor differentiation, and poor overall survival, suggesting that CYP39A1 may serve as a tumor suppressor gene and novel biomarker for HCC patients[25].

1. **IGSF22**

The IGSF22 gene, also known as Immunoglobulin superfamily member 22, encodes a protein that belongs to the immunoglobulin superfamily. This protein is involved in cell-cell adhesion and communication processes. While the exact function of IGSF22 is not fully understood, it is believed to play a role in various cellular processes, including cell adhesion, migration, and signaling[26].

1. **FGFR3**

The FGFR3 gene, also known as Fibroblast Growth Factor Receptor 3, encodes a protein that belongs to the fibroblast growth factor receptor family. This receptor is a tyrosine kinase that plays a crucial role in cell proliferation, differentiation, and survival by binding to fibroblast growth factors (FGFs). Due to frequent mutations in certain cancers, *FGFR3* gene is considered as an oncogene. However, in some normal tissues, *FGFR3* can limit cell growth and promote cell differentiation. Thus, *FGFR3* action appears paradoxical[27].

1. **GFI1**

The GFI1 gene, or Growth Factor Independence 1, encodes a zinc finger protein that functions as a transcriptional repressor. It plays crucial roles in regulating cell differentiation, proliferation, and survival in various cell types, including hematopoietic cells and neurons. GFI1 is a transcriptional repressor with an important role in human malignancies, including leukemia, colorectal carcinoma, and lung cancer, but its role in prostate and breast cancer is unknown. We have found that Gfi1 epigenetic silencing is a common event in prostate and breast cancer. Gfi1 re-expression in prostate and breast cancer cell lines displaying Gfi1 epigenetic silencing decreases cell proliferation, reduced colony formation density, and tumor growth in nude mice xenografts[28].

1. **FLT3**

The FLT3 gene, or FMS-like tyrosine kinase 3, encodes a receptor tyrosine kinase that plays a crucial role in hematopoiesis, specifically in the development and function of hematopoietic stem cells and progenitor cells. FLT3 is also known as CD135. Mutations in the receptor tyrosine kinase Flt3 represent a very common genetic lesion in acute myeloid leukemia (AML). Internal tandem duplication (ITD) mutations clustered in the juxtamembrane domain are the most frequent and best characterized mutations found in Flt3. Oncogenic activation of Flt3 by ITD mutations is known to activate aberrant signaling including activation of STAT5 and repression of myeloid transcription factors Pu.1 and c/EBP-alpha. However, the mechanisms of STAT5 activation by Flt3-ITD remain unclear[29].

1. **KLHL15**

The KLHL15 gene encodes a protein called kelch-like protein 15, which belongs to the kelch-like family of proteins. Kelch-like proteins are involved in various cellular processes, including protein degradation, cytoskeleton organization, and signal transduction. KLHL15 is a substrate adaptor for cullin3-containing E3 ubiquitin ligases, and KLHL15 gene mutations were recently described as a cause of severe X-linked intellectual disability[30].

1. **AC139100.3**

The AC139100.3 gene, also known as LINC02454, is a long intergenic non-protein coding RNA (lncRNA) encoded by the AC139100.3 locus. LncRNAs are a class of RNA molecules that do not code for proteins but play important roles in regulating gene expression and various cellular processes. No complete information regarding functionality of gene AC139100.3.

1. **FOXI2**

The FOXI2 gene encodes a member of the forkhead box (FOX) family of transcription factors, specifically belonging to the FOXI subfamily. FOXI transcription factors play important roles in various developmental processes, particularly in the development and function of epithelial tissues. FOXF2 inhibits proliferation, invasion, migration, metastasis and drug resistance of breast cancer cells. The FOXF2 gene is often silenced in luminal-type and HER2-positive breast cancers. Further studies showed that FOXF2 could help block the G1-S transition of the cell cycle by inhibiting the CDK2-RB-E2F cascade, thus inhibiting the development of luminal-type and HER2-positive breast cancer[31].

1. **AC138035.2**

The gene AC138035.2 is a non-coding RNA gene, also known as a long non-coding RNA (lncRNA). Long non-coding RNAs are a class of RNA molecules that are transcribed from the genome but do not encode proteins. Instead, they play diverse regulatory roles in the cell, often by interacting with other molecules such as DNA, RNA, and proteins. Several lines of evidence suggest that lncRNAs interfere with miRNA activity as endogenous sponges. Based on the constructed lncRNA–miRNA–mRNA network, it has been observed that many lncRNAs contained one or more miRNA binding sites. LncRNAs (AC138035.2, ISPD-AS1, LINC01030, PARD6G-AS1, and AC023481.1) interacted with SMAD5, IL7R, MAP2K6, and PFN2, through competitively binding with miR-493-5p or miR-148a-5p[32].

1. **C9orf16**

The gene C9orf16, also known as Chromosome 9 Open Reading Frame 16, is located on chromosome 9 in humans. Its specific function is not fully understood, and it is classified as an open reading frame (ORF) gene, indicating that it has the potential to encode a protein. However, its protein product and exact biological role have not been extensively characterized. **B**y bioinformatic analysis of single cell RNA-sequencing data on normal pancreas tissues, primary and metastatic PDAC tumors, it has been identified a promising PDAC biomarker, C9orf16. The expression of C9orf16, rarely detectable in normal epithelial cells, was upregulated in primary PDAC cancer cells and was further elevated in metastatic PDAC cancer cells. Gain or loss of function of C9orf16 demonstrated its critical functions in regulating the cell proliferation, invasion and chemotherapy resistance of cancer cells. Pathway analysis and functional studies identified MYC signaling pathways as the most activated pathways in regulating C9orf16 expression and in mediating the development and progression of PDAC[33].

1. **RP11-755E23.3**

The gene RP11-755E23.3, also known as Chromosome 11 Open Reading Frame 70 (C11orf70), is an open reading frame (ORF) located on chromosome 11 in humans. Like many ORF genes, its specific function and biological role are not yet fully understood. These genes are typically identified through genome sequencing efforts but lack well-defined functional annotations. No complete information regarding functionality of gene RP11-755E23.3.

1. **FHL2**

The FHL2 gene, also known as four and a half LIM domains protein 2, is a human gene located on chromosome 2. It encodes a protein that contains four and a half LIM domains, which are involved in protein-protein interactions and play roles in various cellular processes such as cell signaling, gene expression regulation, and cytoskeleton organization. LIM domain protein 2, also known as LIM protein FHL2, is a member of the LIM-only family. Due to its LIM domain protein characteristics, FHL2 is capable of interacting with various proteins and plays a crucial role in regulating gene expression, cell growth, and signal transduction in muscle and cardiac tissue. In recent years, mounting evidence has indicated that the FHLs protein family is closely associated with the development and occurrence of human tumors. On the one hand, FHL2 acts as a tumor suppressor by down-regulating in tumor tissue and effectively inhibiting tumor development by limiting cell proliferation. On the other hand, FHL2 serves as an oncoprotein by up-regulating in tumor tissue and binding to multiple transcription factors to suppress cell apoptosis, stimulate cell proliferation and migration, and promote tumor progression. Therefore, FHL2 is considered a double-edged sword in tumors with independent and complex functions[34].

1. **FKBPL**

The FKBPL gene, also known as FK506-binding protein-like, is a human gene located on chromosome 6. It encodes a protein that shares structural similarities with FK506-binding proteins (FKBPs) but does not possess peptidyl-prolyl cis-trans isomerase activity like typical FKBPs. FKBPL was identified by using an shRNA genetic screen library as a regulator of breast cancer tumour initiation, and high tumour *Fkbpl* and low *Nanog* are associated with improved survival outcomes in breast cancer patients [35].

1. **FOXH1**

The FOXH1 gene, also known as Forkhead box protein H1 or FAST-1, is a human gene located on chromosome 8. It encodes a transcription factor that belongs to the forkhead box (FOX) family of proteins. FOXH1 overexpression promotes the proliferation, migration, and invasion of colorectal cancer cells in vivo by down-regulating E-cadherin level. In acute myeloid leukemia, FOXH1 is a critical mediator of the functions of mutant p53 that binds to and regulates stem cell-associated genes and transcriptional programs. Studies have shown that mutant p53 appears to promote leukemia by enforcing the expression of FOXH1, which is involved in promoting stemness and cell plasticity during hematopoiesis[36].

1. **FGF20**

The FGF20 gene encodes the fibroblast growth factor 20 protein, which is a member of the fibroblast growth factor (FGF) family. FGF20 is involved in various developmental processes and plays a role in the regulation of cell proliferation, differentiation, and migration. Elevated levels of FGF20 RNA were also observed in adenomas from mice carrying the Apc^Min^allele. Both XFGF20 and Xdkk-1 are expressed early in Xenopus embryogenesis under the control of the Wnt signaling pathway. Furthermore, FGF20 and DKK1 appear to be direct targets for β-catenin/TCF transcriptional regulation via LEF/TCF-binding sites. Finally, by using small inhibitory RNAs specific for FGF20, it has been shown that continued expression of FGF20 is necessary for maintenance of the anchorage-independent growth state in RK3E cells transformed by β-catenin, implying that FGF-20 may be a critical element in oncogenesis induced by the Wnt signaling pathway[37].

1. **PPFIA1**

The PPFIA1 gene, also known as Liprin-alpha-1, encodes a member of the Liprin family of proteins. These proteins are involved in the regulation of synaptic development and function, particularly at neuronal synapses. Liprin-alpha-1 plays a role in the assembly and organization of protein complexes at the synapse, contributing to synaptic structure and signaling. PPFIA1 is an important regulator of cell migration and invasion, regulating focal adhesion signaling and disassembly. PPFIA1 is frequently amplified in breast cancer, and recent functional studies indicate that PPFIA1 is an important promoter of migration and invasion in breast cancer[38].

1. **ALG1**

The ALG1 gene encodes an enzyme called alpha-1,2-mannosyltransferase, which is involved in the process of protein glycosylation. Glycosylation is the attachment of sugar molecules to proteins, which is important for protein folding, stability, and function. ALG1 participates in the initial stage of protein N-glycosylation and N-glycosylation has been implicated in the process of hepatocellular carcinoma (HCC) progression[39].

1. **LL22NC03-2H8.4**

The gene LL22NC03-2H8.4 is a long non-coding RNA (lncRNA) gene, which means it does not code for proteins but instead plays regulatory roles in various cellular processes. LncRNAs are a diverse class of RNA molecules that have been implicated in gene expression regulation, chromatin remodeling, and other cellular functions. No complete information regarding functionality of gene LL22NC03-2H8.4.

1. **FKBP2**

The FKBP2 gene encodes a protein called FK506-binding protein 2, also known as FKBP13. This protein belongs to the FK506-binding protein (FKBP) family, which are peptidyl-prolyl cis-trans isomerases that catalyze the interconversion of cis and trans peptide bonds. FKBP2 is overexpressed in the hypoxic environment of glioblastoma multiforme and triggers tumor metastasis. FKBP family members are both significant prognostic biomarkers for lung cancer progression and promising clinical therapeutic targets, thus providing new targets for treating LUAD patients[40].

1. **INAFM1**

The gene INAFM1, also known as Inhibitor of Activated STAT, is a protein-coding gene involved in regulating the Janus kinase/signal transducers and activators of transcription (JAK/STAT) signaling pathway. The expression INAFM1after additional studies and validation in the extended cohort, could serve as a prognostic marker of locally advanced lymph node-positive Prostate cancer[41].

1. **CABYR**

The gene CABYR, also known as Calcium-binding tyrosine phosphorylation-regulated protein, is a protein-coding gene involved in sperm motility and fertilization. CABYR is primarily expressed in the testes, where it plays a role in sperm flagellar function and regulation. CABYR-c is highly expressed in hepatocellular carcinoma tissues and may play an oncogenic role in hepto-carcinogenesis as well as its progression[42].

1. **INCENP**

The INCENP gene, also known as inner centromere protein, plays a crucial role in cell division and chromosome segregation during mitosis and meiosis. INCENP is a key component of the chromosomal passenger complex (CPC), which regulates various aspects of cell division, including chromosome alignment, spindle assembly, and cytokinesis. The CPC plays a pivotal role in the regulation of cell division. Therefore, inherited CPC variability could influence tumor development. The present candidate gene approach investigates the relationship between single nucleotide polymorphisms (SNPs) in genes encoding key CPC components and breast cancer risk. Fifteen SNPs in four CPC genes (INCENP, AURKB, BIRC5 and CDCA8) were genotyped in 88 911 European women from 39 case-control studies of the Breast Cancer Association Consortium[43].

1. **LLOXNC01-237H1.2**

The gene LLOXNC01-237H1.2 appears to be an uncharacterized long non-coding RNA (lncRNA), based on its name. Long non-coding RNAs are a diverse class of RNA molecules that do not encode proteins but instead play regulatory roles in various cellular processes. No complete information regarding functionality of gene LLOXNC01-237H1.2.

1. **LIPM**

LIPM cDNA / gene is a gene with protein product which located on 10q23.31. The LIPM gene is conserved in chimpanzee, Rhesus monkey, dog, cow, mouse, rat, fruit fly, and mosquito. 102 organisms have orthologs with human gene LIPM. The overexpression of BPIFB1 and high initial B-cell infiltration levels are linked to early cancer recurrence, while the overexpression or amplification of ANKRD22 and LIPM, mutation of IGHA1 and MUC16, high fibroblast infiltration level, M1 polarization of macrophages, cellular status of DNA repair are all linked to early cancer metastasis[44].

1. **AC125421.1**

The gene AC125421.1 is an example of a non-coding RNA (ncRNA). It is classified as a long non-coding RNA (lncRNA) based on its name. Long non-coding RNAs are RNA molecules that are transcribed from DNA but do not code for proteins. Instead, they have diverse functions in gene regulation, chromatin organization, and other cellular processes. No complete information regarding functionality of gene AC125421.1.

1. **RP11-146F11.1**

The gene RP11-146F11.1 is a long non-coding RNA (lncRNA). Long non-coding RNAs are RNA molecules that are transcribed from DNA but do not encode proteins. Instead, they have diverse roles in regulating gene expression, chromatin organization, and other cellular processes. No complete information regarding functionality of gene RP11-146F11.1.

1. **GPX1**

The gene GPX1, also known as Glutathione Peroxidase 1, encodes an enzyme called glutathione peroxidase. Glutathione peroxidase plays a crucial role in protecting cells from oxidative damage by catalyzing the reduction of hydrogen peroxide and lipid hydroperoxides to water and alcohols, respectively, using glutathione as a cofactor. GPX1 is abnormally elevated in most types of cancer but has complex dichotomous roles as tumor suppressor and promoter in different cancers. GPX1 can participate in various signaling pathways to regulate tumor biological behaviors, including cell proliferation, apoptosis, invasion, immune response, and chemoresistance[45].

1. **FKRP**

The FKRP gene is mutated in a severe form of CMD (MDC1C), characterized by early onset, inability to achieve independent ambulation, muscle hypertrophy, marked elevation of serum creatine kinases (CK), no brain involvement and a secondary deficiency of laminin α2 ([15](javascript:;)). In addition, affected individuals had a marked decrease in immunostaining of muscle α-dystroglycan and a reduction in its molecular weight on western blot analysis. Mutations in the FKRP gene result in a broad spectrum of muscular dystrophy (MD) phenotypes, including the severe Walker-Warburg syndrome[46].

1. **LINC01620**

The gene LINC01620, also known as long intergenic non-protein coding RNA 1620, is a member of the long non-coding RNA (lncRNA) family. LncRNAs are RNA molecules that are longer than 200 nucleotides in length and do not code for proteins. No complete information regarding functionality of gene LINC01620!

1. **CA12**

CA12 is a transmembrane protein involving in cellular pH regulation of metabolically active cells/tissues by catalyzing a reversible reaction of carbon dioxide hydration and dehydration: H_2_O + CO_2_ ⇄ H + HCO3. CA12 has been identified as a potential target for therapeutic applications because of its over-expression in human malignant tumors. CA12 can be up-regulated concurrently and interacted with P-glycoprotein in chemotherapy-resistant cells. CA12 silencing decreased the ATPase activity of Pgp by altering the optimal pH at which Pgp operated and promoted chemosensitization to Pgp substrates in MDR cells. The acidic environment of tumor cells also reduce killing efficacy of T lymphocyte. In order to avoid the potentially lethal consequences of excessive acidification of the cell microenvironment, cancer cells can automatically up-regulate some molecules such as carbonic anhydrase 12 (CA12) and maintain stable pH gradient to promote the survival, proliferation, and invasion of cancer cells. This phenomenon may give rise to escape immune surveillance functions in patients with cancer. The CA12 protein has high expression in breast cancer cell line MCF-7 and MCF-7 TaxR[47].

**Patient 3**

1. **PHACTR2-AS1**

The gene PHACTR2-AS1, also known as Phosphatase and actin regulator 2 antisense RNA 1, is an antisense long non-coding RNA (lncRNA) that is transcribed from the opposite DNA strand of the PHACTR2 gene. Antisense RNAs are complementary to messenger RNAs (mRNAs) and can regulate gene expression through various mechanisms. In breast tissue, PHACTR2-AS1 is suggested to be a tumor-suppressor gene for breast cancer, and injecting this lncRNA fragment into mice could inhibit tumor growth and metastasis (24). By contrast, this lncRNA was suggested to be an oncogene for hepatocellular, gastric and tongue squamous cell carcinoma (10–12,25)[48].

1. **SFI1**

The gene SFI1, also known as Spindle Formation 1, plays a crucial role in centrosome duplication and spindle formation during cell division.Sfi1 was identified as a candidate tumor driver in numerous screens, but was subsequently found to be a false call due to inaccurate mapping of reads to repetition-rich regions of the genome (63). It is inevitable that algorithms used to interpret the vast amount of genetic data generated from SB screens will continue to improve[49].

1. **RP3-414A15.2**

The gene RP3-414A15.2 is a type of non-coding RNA, meaning it does not code for a protein but instead plays regulatory roles in the cell. No complete information regarding functionality of gene RP3-414A15.2!

1. **SRR**

The term "SRR" typically refers to a Sequence Read Archive (SRA) Run accession number. The Sequence Read Archive is a public repository that stores high-throughput sequencing data, such as those generated by technologies like next-generation sequencing (NGS). Each SRA Run accession (SRR) corresponds to a specific set of sequencing data generated from a biological sample. SRR may function as a tumor suppressor gene in some cancers, such as KIRC and UCEC, based on expression and survival analysis[50].

1. **NRG4**

The gene NRG4, also known as Neuregulin 4, plays important roles in various physiological processes, including development, tissue homeostasis, and metabolism. It has been reported previously that high levels of NRG4 expression (anti-123) in prostate cancer are associated with advanced tumor stage and described the expression of NRG4A2 mRNA in cell lines derived from metastatic prostate disease and not those derived from localized disease. It is also conceivable that NRG4 is influencing disease spread and is associated with advanced tumor stage[51].

1. **PITPNC1**

The gene PITPNC1, also known as Phosphatidylinositol Transfer Protein Cytoplasmic 1, is involved in various cellular processes, including intracellular lipid transport and signaling pathways. PITPNC1 drives metastasis by regulating the secretion of pro-invasive and pro-angiogenic genes. Multiple steps in the metastatic cascade are orchestrated by the secretion of regulatory proteins from cancer cells in the microenvironment[52].

1. **SGIP1**

The SGIP1 gene, also known as SH3-GL Domain-Containing Integrin-Linked Kinase-Interacting Protein 1, is involved in cellular processes related to membrane trafficking and receptor endocytosis. SGIP1 functions as an endocytic protein that has effects on signaling in neuronal systems including energy homeostasis. Hypomethylation and retrotransposition of SGIP1 have been reported in colorectal cancer samples[53].

1. **LEKR1**

The LEKR1 gene is a protein-coding gene that is associated with various physiological processes in the human body. LEKR1 (Leucine, Glutamate And Lysine Rich 1) is a Protein Coding gene. Diseases associated with LEKR1 include Obsessive-Compulsive Personality Disorder. Gene Ontology (GO) annotations related to this gene include structural constituent of ribosome. The protein encoded by *LEKR1* has also not been well characterized, but a missense variant in *LEKR1* was previously associated with epithelial ovarian cancer[54].

1. **AC114765.2**

The AC114765.2 gene is a non-protein-coding gene, also known as a long non-coding RNA (lncRNA), that is transcribed from the DNA but not translated into a protein. No complete information regarding functionality of gene AC114765.2.

1. **RP11-561B11.2**

The RP11-561B11.2 gene is a gene identifier typically assigned to a region of the genome that has been sequenced and annotated but does not necessarily correspond to a known gene with a specific function. No complete information regarding functionality of gene AC114765.2.

1. **SERINC4**

The Serine incorporator SERINC4 gene, also known as Serine Incorporator 4, is a protein-coding gene found in humans. SERINC proteins 1–5 (SERINC1-5) are involved in the progression of several diseases. SERINC2-4 are carrier proteins that incorporate the polar amino acid serine into membranes to facilitate the synthesis of phosphatidylserine and sphingolipids. SERINC genes are also differentially expressed in tumors. Abnormal expression of SERINC proteins occurs in human cancers of the breast, lung, colon, liver, and various glands, as well as in mouse testes. SERINC proteins also affect cleft lip and palate and nerve-related diseases, such as seizure Parkinsonism and borderline personality. Moreover, SERINC proteins have garnered significant interest as retroviral restriction factors, spurring efforts to define their function and elucidate the mechanisms through which they operate when associated with viruses. Human SERINC proteins possess antiviral potential against human immunodeficiency virus (HIV), SARS-COV-2, murine leukemia virus (MLV), equine infectious anemia virus (EIAV), and hepatitis B virus (HBV). Furthermore, the crystal structure is known, and the critical residues of SERINC5 that act against HIV have been identified. In this review, we discuss the most prevalent mechanisms by which SERINC3 and SERINC5 antagonize viruses and focus on the potential therapeutic applications of SERINC5/3 against HIV[55].

1. **SH3GLB2**

The SH3GLB2 gene, also known as SH3 domain-containing GRB2-like 2, plays a role in various cellular processes. Loss of Sh3gl2 was associated with increasing tumor grade and with muscle invasion, which is a reliable predictor of metastatic disease and cancer-derived mortality. Sh3gl2 expression was undetectable in 19 of 20 human UC cell lines but preserved in the low-grade cell line RT4. Stable silencing of Sh3gl2 in RT4 cells by RNA interference 1) enhanced proliferation and colony formation in vitro, 2) inhibited EGF-induced EGFR internalization and increased EGFR activation, 3) stimulated phosphorylation of Src family kinases and STAT3, and 4) promoted growth of RT4 xenografts in subrenal capsule tissue recombination experiments. Conversely, forced re-expression of Sh3gl2 in T24 cells and silenced RT4 clones attenuated oncogenic behaviors, including growth and migration. Together, these findings identify loss of Sh3gl2 as a frequent event in UC development that promotes disease progression[56].

1. **SPNS3**

Spinster homolog 3 (SPNS3) belongs to the Spinster (SPNS) family which participates in sphingolipid transportation through the cell membrane. However, the functions of SPNS3 in acute myeloid leukemia (AML) are unknown. This study obtained SPNS3 from a gene set that was related to AML relapse and evaluate whether high SPNS3 expression induced apoptosis resistance in an AML cell line, which is consistent with the role of SPNS3 as a marker of poor prognosis in the clinic. Moreover, internal tandem duplication of FMS-like tyrosine kinase 3 (FLT3-ITD) mutation and the AC127521.1/ MIR-139/SPNS3 competing endogenous RNA axis were found to regulate SPNS3 expression. In addition, SPNS3 may play an important role in the Sphingosine-1-phosphate signal pathway that is involved in the maintenance of the AML microenvironment. These results highlight the anti-apoptosis effect of SPNS3 in AML, and the potential mechanism mediating this effect was explored through bioinformatics[57].

1. **RP11-58015.1**

The gene RP11-58015.1 is a gene symbol that represents a long non-coding RNA (lncRNA) transcript. RP11 has no effect on mRNA expression but increases the protein expression of Zeb1 in CRC cells by increasing Zeb1 protein stability and decreasing Zeb1 ubiquitination. By screening for factors responsible for the stability of Zeb1 in cancer cells, we confirm that the downregulation of Siah1 and Fbxo45 mediates the RP11-induced stabilization of Zeb1 in CRC cells. No complete information regarding functionality of gene RP11-58015.1.

1. **CTA-796E4.4**

The gene CTA-796E4.4 is a non-coding RNA (ncRNA) transcript. The expression level of CTA-796E4.4 in bladder cancer tissue was lower than that in para cancerous tissue and the difference was statistically significant (P < 0.01). The expression of CTA-796E4.4 in bladder cancer cells was lower than that in bladder cancer epithelial cells. The expression of lncRNA CTA-796E4.4 is decreased in bladder cancer and cell lines. High expression of CTA-796E4.4 inhibits the proliferation and invasion of UM-UC-3 cells. The molecular mechanism may be that up-regulation of CTA-796E4.4 can promote the expression of FOXO1[58].

1. **AC104777.2**

The gene AC104777.2 is a non-coding RNA (ncRNA) transcript. No complete information regarding functionality of gene AC104777.2.

1. **AC140725.4**

The gene AC140725.4 is a non-coding RNA (ncRNA) transcript. No complete information regarding functionality of gene AC140725.4.

1. **SFR1**

Estrogen receptor alpha (ERα), a ligand-dependent transcription factor, mediates the expression of its target genes by interacting with corepressors and coactivators. Since the first cloning of SRC1, more than 280 nuclear receptor cofactors have been identified, which orchestrate target gene transcription. Aberrant activity of ER or its accessory proteins results in a number of diseases including breast cancer. It has been identified as a protein involved in DNA homologous recombination, as a novel binding partner of ERα. Initially isolated in a yeast two-hybrid screen, the interaction of SFR1 and ERα was confirmed in vivo by immunoprecipitation and mammalian one-hybrid assays. SFR1 co-localized with ERα in the nucleus, potentiated ER’s ligand-dependent and ligand-independent transcriptional activity, and occupied the ER binding sites of its target gene promoters. Knockdown of SFR1 diminished ER’s transcriptional activity. Manipulating SFR1 expression by knockdown and overexpression revealed a role for SFR1 in ER-dependent and -independent cancer cell proliferation. SFR1 differs from SRC1 by the lack of an intrinsic activation function. Taken together, we propose that SFR1 is a novel transcriptional modulator for ERα and a potential target in breast cancer therapy[59].

1. **PKM**

The gene PKM, also known as pyruvate kinase M, is involved in glycolysis, which is a central metabolic pathway responsible for the conversion of glucose into pyruvate. It is the most-studied isoform of pyruvate kinase and catalyzes the final step in glycolysis. It is one of the key mediators of the Warburg effect and plays a pivotal role in controlling tumor metabolism [60].

1. **RP3-503A6.2**

The gene RP3-503A6.2 is a non-coding RNA gene, meaning it does not code for proteins but instead produces functional RNA molecules. No complete information regarding functionality of gene RP3-503A6.2.

1. **RP3-441A12.1**

The gene RP3-441A12.1 is a non-coding RNA gene, which means it does not code for proteins but instead produces functional RNA molecules. No complete information regarding functionality of gene RP3-441A12.1.

1. **FAM65C**

The FAM65C gene, also known as Family with sequence similarity 65 member C, is a protein-coding gene located on human chromosome 2. Two of the genes HSD17B13 and FAM65C that are down-regulated by LOC101926913 (LOC) in *in vitro* experiments were also found to be negatively correlated with LOC in Hepatocellular Carcinoma (HCC) patient tissues (|PCC|>0.6). FAM65C was also reported to be downregulated in HCC although very little is known about this protein. It is thus worthwhile to further characterize how lncRNA LOC101926913 deregulate the expression of HSD17B13 and FAM65C to modulate patient outcome[61].

1. **AC144831.1**

The gene AC144831.1 is a non-coding RNA gene located in the human genome. Renal cell carcinoma is one of the most universal urinary system cancers in the world. The most common renal cell carcinoma subtype is renal clear cell carcinoma. It is usually associated with high rates of metastasis and mortality. Therefore, finding effective therapeutic targets and prognostic molecular markers is of great significance to improve the early diagnosis rate and prognostic accuracy of renal clear cell carcinoma. Six hub genes has been identified as closely related to the occurrence, development and prognosis of renal clear cell carcinoma and proposed three new potential prognostic markers, namely ATP4B, AC144831.1 and Tfcp2l1 through differentially expressed genes (DEGs) analysis, GO functional enrichment and KEGG pathway analysis, WGCNA analysis, and survival analysis. In addition, we established machine learning models to predict the occurrence of tumors through the gene expression data of patients. It is expected that the results of this study can provide reference value for the treatment of renal clear cell carcinoma[62].

1. **RP11-432J24.5**

The gene RP11-432J24.5 is a human gene, also known as long non-coding RNA (lncRNA) RP11-432J24.5. This gene is located on chromosome 1 and is classified as a long non-coding RNA because it does not encode a protein. Instead, it is involved in regulating various cellular processes at the transcriptional or post-transcriptional level. The specific functions and roles of RP11-432J24.5 may vary depending on the context of the cell type and biological conditions. Like many lncRNAs, RP11-432J24.5 may participate in gene regulation, chromatin remodeling, or signaling pathways involved in development, differentiation, or disease processes[63].

1. **RP11-43F13.3**

The gene RP11-432J24.5 is a protein-coding gene located in the human genome. Combined with clinicopathological data, an LIHC prognostic risk model comprising 9 m7G-related lncRNAs was finally achieved by univariate, LASSO, and multivariate Cox regression analyses successively. Five lncRNAs (SOCS2-AS1, RP5-1171I10.5, RP11-588H23.3, RP11-10A14.3, and NAV2-AS4) were identified to be a protective factor for LIHC prognosis, and other four lncRNAs (RP11-43F13.3, RP11-95O2.5, RP11-519G16.5, and RP11-874J12.4) were risk factors affecting the prognosis of LIHC. SOCS2-AS1 has recently been validated as a tumor suppressor in colorectal and endometrial cancers, and its low expression in tumors was positively related to poor prognosis of patients[64].

1. **NHSL1**

The gene NHSL1, also known as NHS-like 1, is a protein-coding gene located on the human genome. A novel metastasis-related circRNA circNHSL1 from exons of the NHS like-1 (NHSL1), with a circBase ID of hsa_circ_0006835 has been identified. Also, circNHSL1 was up-regulated in both gastric cancer tissues and cell lines, and correlated with advanced clinical stage, distant metastasis, lymph node metastasis and poor prognosis. Importantly, circNHSL1 promoted invasion and metastasis of gastric cancer by acting as a miR-1306-3p sponge to relieve its repression on target SIX1. Furthermore, it has been demonstrated that SIX1 enhanced the expression of Vimentin in transcriptional level by directly binding to the promoter domain of Vimentin[65].

1. **PKD1L3**

The PKD1L3 gene encodes an ion channel protein that can interact with the PKD2L1 protein to form a candidate sour taste receptor. In the present study, we have analyzed the evolutionary patterns of PKD1L3 genes from 10 mammalian species. The results showed that PKD1L3 genes have evolved under a dominant purifying selection force. However, for some branches and sites, PKD1L3 genes were detected to have been operated by positive selection. Moreover, some of these positive evolutionary sites are likely to participate in acid stimulus recognition. In rodents, PKD1L3 genes evolved more rapidly than other mammalian lineages. Combined with other functional research reports, our results suggest that rodents may not be the most appropriate model for functional research on the PKD1L3 gene[66].

1. **RP4-535B20.4**

The RP4 plasmid has various genetic features to maintain self-replication, transmissibility, and survival in a wide range of Gram-negative and Gram-positive bacteria. Previous studies have shown the transfer of the RP4 plasmid from laboratory strains of E. coli to bacteria isolated from soil, sewage, and activated sludge. In a recent study, Heß et al. reported that in microcosm experiments, RP4 plasmid transferred to multiple bacterial strains from three donor E. coli strains. These past studies on RP4 conjugation were mainly conducted using laboratory or environmental donor E. coli strains. However, a major knowledge gap exists in understanding the transfer of a broad-host-range plasmid such as the RP4 to other bacteria of the human gut microbiota. No complete information regarding functionality of gene RP4-535B20.4 [67].

1. **NRBF2**

The gene NRBF2, also known as Nuclear Receptor Binding Factor 2, is a protein-coding gene found in humans. Reversing chemotherapy resistance in small cell lung cancer (SCLC) is crucial to improve patient prognosis. It has been shown that NRBF2 is a poor prognostic factor in SCLC. The effects of NRBF2 on chemoresistance were determined in SCLC. The molecular mechanisms of NRBF2 in the autophagy process in SCLC were examined. NRBF2 positively regulated autophagy, leading to drug resistance in SCLC. The MIT domain of NRBF2 directly interacted with the PB1 domain of P62. This interaction increased autophagic P62 body formation, revealing the regulatory role of NRBF2 in autophagy. Notably, NRBF2 was directly modulated by the transcription factor XRCC6. The MIT domain of NRBF2 interacts with the PB1 domain of P62 to regulate the autophagy process, resulting in SCLC chemoresistance. NRBF2 is likely a useful chemotherapy response marker and therapeutic target in SCLC[68].

1. **LCNL1**

LCN1, also called tear lipocalin (TLC), is a member of the lipocalin family, which is involved in various physiological processes such as transport of small hydrophobic molecules and immune response modulation. While there may not be direct evidence linking LCNL1 to cancer, its expression patterns and functions in different tissues and biological contexts could potentially influence cancer development and progression. LCN1 binds to macromolecules, which regulate tear viscosity, the binding and release of lipids, and endonuclease inactivation of viral DNA. Hepatic overexpression of apolipoprotein M (APOM) in low-density-lipoprotein-receptor-deficient mice has been shown to lead to an approximately 70% reduction in atherosclerosis[69].

**References**

1. Bensmana M, Huck S, Lefranc G, Lefranc M. The human immunoglobulin pseudo-gamma IGHGP gene shows no major structural defect. Nucleic acids research. 1988;16(7):3108.

2. Li L, Liu J, Huang W. E2F5 promotes proliferation and invasion of gastric cancer through directly upregulating UBE2T transcription. Digestive and Liver Disease. 2022;54(7):937–45.

3. Zhang Q, Song X, Song X. Contents in tumor-educated platelets as the novel biosource for cancer diagnostics. Frontiers in Oncology. 2023;13:1165600.

4. Hu H, Wang Z, Li M, Zeng F, Wang K, Huang R, et al. Gene expression and methylation analyses suggest DCTD as a prognostic factor in malignant glioma. Scientific reports. 2017;7(1):11568.

5. Li K, Pan W, Ma Y, Xu X, Gao Y, He Y, et al. A novel oncogene TRIM63 promotes cell proliferation and migration via activating Wnt/β-catenin signaling pathway in breast cancer. Pathology-Research and Practice. 2019;215(10):152573.

6. Ma HR, Cao L, Wang F, Cheng C, Jiang R, Zhou H, et al. Filamin B extensively regulates transcription and alternative splicing, and is associated with apoptosis in HeLa cells. Oncology Reports. 2020;43(5):1536–46.

7. Luo Y, Zhang M, Wang Z, Li Z, Chen X, Cao J, et al. Effects of SVEP1 on Lung Squamous Cell Carcinoma and its Association with Tumor Mutation Burden, Prognosis, and Immune Regulation. Combinatorial Chemistry & High Throughput Screening. 2023;26(2):313–29.

8. Della Corte CM, Viscardi G, Di Liello R, Fasano M, Martinelli E, Troiani T, et al. Role and targeting of anaplastic lymphoma kinase in cancer. Molecular cancer. 2018;17(1):30.

9. Huang Z, Shan R, Wen W, Li J, Zeng X, Wan R. The Emerging Roles of Circ-ABCB10 in Cancer. Frontiers in Cell and Developmental Biology. 2022;10:782938.

10. Lehnart SE, Wehrens XH. The role of junctophilin proteins in cellular function. Physiological reviews. 2022;102(3):1211–61.

11. Li X, Cheng R. TPO as an indicator of lymph node metastasis and recurrence in papillary thyroid carcinoma. Scientific Reports. 2023;13(1):10848.

12. Fadaka A, Bakare O, Sibuyi N, Klein A. Gene Expression Alterations and Molecular Analysis of CHEK1 in Solid Tumors. Cancers. 2020; 12: 662.

13. Qi Y, Wang M, Jiang Q. PABPC1——mRNA stability, protein translation and tumorigenesis. Frontiers in Oncology. 2022;12:1025291.

14. Li F, Zhao H, Su M, Xie W, Fang Y, Du Y, et al. HnRNP-F regulates EMT in bladder cancer by mediating the stabilization of Snail1 mRNA by binding to its 3′ UTR. EBioMedicine. 2019;45:208–19.

15. Sudhakaran M, Doseff AI. Role of heterogeneous nuclear ribonucleoproteins in the cancer-immune landscape. International Journal of Molecular Sciences. 2023;24(6):5086.

16. Miao H-K, Chen L-P, Cai D-P, Kong W-J, Xiao L, Lin J. MSH3 rs26279 polymorphism increases cancer risk: a meta-analysis. International journal of clinical and experimental pathology. 2015;8(9):11060.

17. Yu S, Sun L, Jiao Y, Lee LTO. The role of G protein-coupled receptor kinases in cancer. International journal of biological sciences. 2018;14(2):189.

18. Cen X, Lu Y, Lu J, Luo C, Zhan P, Cheng Y, et al. Heat shock protein HSPA13 promotes hepatocellular carcinoma progression by stabilizing TANK. Cell Death Discovery. 2023;9(1):443.

19. Hankey W, Frankel WL, Groden J. Functions of the APC tumor suppressor protein dependent and independent of canonical WNT signaling: implications for therapeutic targeting. Cancer and Metastasis Reviews. 2018;37:159–72.

20. Cai Q, Yang H-S, Li Y-C, Zhu J. Dissecting the roles of PDCD4 in breast cancer. Frontiers in Oncology. 2022;12:855807.

21. Ji M, Zhao Z, Li Y, Xu P, Shi J, Li Z, et al. FBXO6-mediated RNASET2 ubiquitination and degradation governs the development of ovarian cancer. Cell Death & Disease. 2021;12(4):317.

22. Kolmus K, Erdenebat P, Szymańska E, Stewig B, Goryca K, Derezińska-Wołek E, et al. Concurrent depletion of Vps37 proteins evokes ESCRT-I destabilization and profound cellular stress responses. Journal of cell science. 2021;134(1):jcs250951.

23. Li Q, Chen B, Song G, Zeng K, Chen X, Miao J, et al. Integrated analysis to identify the AC005154. 6/hsa-miR-29c-3p/CCNL2 axis as a novel prognostic biomarker associated with immune infiltration in prostate cancer. Cancer cell international. 2022;22(1):346.

24. Seillier M, Peuget S, Gayet O, Gauthier C, N'guessan P, Monte M, et al. TP53INP1, a tumor suppressor, interacts with LC3 and ATG8-family proteins through the LC3-interacting region (LIR) and promotes autophagy-dependent cell death. Cell Death & Differentiation. 2012;19(9):1525–35.

25. Li D, Yu T, Hu J, Wu J, Feng S, Xu Q, et al. Downregulation of CYP39A1 serves as a novel biomarker in hepatocellular carcinoma with worse clinical outcome. Oxidative Medicine and Cellular Longevity. 2021;2021(1):5175581.

26. Hsu H-M, Chu C-M, Chang Y-J, Yu J-C, Chen C-T, Jian C-E, et al. Six novel immunoglobulin genes as biomarkers for better prognosis in triple-negative breast cancer by gene co-expression network analysis. Scientific reports. 2019;9(1):4484.

27. Lafitte M, Moranvillier I, Garcia S, Peuchant E, Iovanna J, Rousseau B, et al. FGFR3 has tumor suppressor properties in cells with epithelial phenotype. Molecular cancer. 2013;12:1–13.

28. Ashour N, Angulo JC, González-Corpas A, Orea MJ, Lobo MV, Colomer R, et al. Epigenetic regulation of gfi1 in endocrine-related cancers: A role regulating tumor growth. International journal of molecular sciences. 2020;21(13):4687.

29. Choudhary C, Brandts C, Schwable J, Tickenbrock L, Sargin B, Ueker A, et al. Activation mechanisms of STAT5 by oncogenic Flt3-ITD. Blood, The Journal of the American Society of Hematology. 2007;110(1):370–4.

30. Song J, Merrill RA, Usachev AY, Strack S. The X-linked intellectual disability gene product and E3 ubiquitin ligase KLHL15 degrades doublecortin proteins to constrain neuronal dendritogenesis. Journal of Biological Chemistry. 2021;296.

31. He W, Kang Y, Zhu W, Zhou B, Jiang X, Ren C, et al. FOXF2 acts as a crucial molecule in tumours and embryonic development. Cell Death & Disease. 2020;11(6):424.

32. Wu J, Chen R, Shen H, Yan T, Qian Y, Zhang Y, et al. Transcriptome analysis of Ivosidenib-mediated inhibitory functions on non-small cell lung cancer. Frontiers in Oncology. 2021;11:626605.

33. Chen X, Zhang H, Xiao B. C9orf16 represents the aberrant genetic programs and drives the progression of PDAC. BMC cancer. 2022;22(1):1102.

34. Zhang J, Zeng Q, She M. The roles of FHL2 in cancer. Clinical and Experimental Medicine. 2023;23(7):3113–24.

35. Annett S, Moore G, Short A, Marshall A, McCrudden C, Yakkundi A, et al. FKBPL-based peptide, ALM201, targets angiogenesis and cancer stem cells in ovarian cancer. British Journal of Cancer. 2020;122(3):361–71.

36. Zhang J, Zhang X, Yang S, Bao Y, Xu D, Liu L. FOXH1 promotes lung cancer progression by activating the Wnt/β-catenin signaling pathway. Cancer cell international. 2021;21(1):293.

37. Chamorro MN, Schwartz DR, Vonica A, Brivanlou AH, Cho KR, Varmus HE. FGF‐20 and DKK1 are transcriptional targets of β‐catenin and FGF‐20 is implicated in cancer and development. The EMBO journal. 2005;24(1):73–84.

38. Alfarsi LH, El Ansari R, Craze ML, Masisi BK, Ellis IO, Rakha EA, et al. PPFIA1 expression associates with poor response to endocrine treatment in luminal breast cancer. BMC cancer. 2020;20:1–8.

39. Cao X, Shao Y, Meng P, Cao Z, Yan G, Yao J, et al. Nascent proteome and glycoproteome reveal the inhibition role of ALG1 in hepatocellular carcinoma cell migration. Phenomics. 2022;2(4):230–41.

40. Wang C-C, Shen W-J, Anuraga G, Hsieh Y-H, Khoa Ta HD, Xuan DTM, et al. Penetrating exploration of prognostic correlations of the FKBP gene family with lung adenocarcinoma. Journal of Personalized Medicine. 2022;13(1):49.

41. Kudryavtseva AV, Lukyanova EN, Kharitonov SL, Nyushko KM, Krasheninnikov AA, Pudova EA, et al. Bioinformatic identification of differentially expressed genes associated with prognosis of locally advanced lymph node-positive prostate cancer. Journal of bioinformatics and computational biology. 2019;17(01):1950003.

42. Li H, Fang L, Xiao X, Shen L. The expression and effects the CABYR-c transcript of CABYR gene in hepatocellular carcinoma. Bulletin du Cancer. 2012;99(3):E26–E33.

43. Kabisch M, Lorenzo Bermejo J, Dünnebier T, Ying S, Michailidou K, Bolla MK, et al. Inherited variants in the inner centromere protein (INCENP) gene of the chromosomal passenger complex contribute to the susceptibility of ER-negative breast cancer. Carcinogenesis. 2015;36(2):256–71.

44. He D-n, Wang N, Wen X-L, Li X-H, Guo Y, Fu S-h, et al. Multi-omics analysis reveals a molecular landscape of the early recurrence and early metastasis in pan-cancer. Frontiers in Genetics. 2023;14:1061364.

45. Zhao Y, Wang H, Zhou J, Shao Q. Glutathione peroxidase GPX1 and its dichotomous roles in cancer. Cancers. 2022;14(10):2560.

46. Dhoke NR, Kim H, Selvaraj S, Azzag K, Zhou H, Oliveira NA, et al. A universal gene correction approach for FKRP-associated dystroglycanopathies to enable autologous cell therapy. Cell reports. 2021;36(2).

47. Huang T, Tang L, Wang H, Lin L, Fu J. Carbonic anhydrase 12 gene silencing reverses the sensitivity of paclitaxel in drug-resistant breast cancer cells. Bioengineered. 2021;12(2):9806–18.

48. Shi Q, Shi Q-N, Xu J-W, Wang H-Y, Li Y-J, Zhang X-X, et al. rs9390123 and rs9399451 influence the DNA repair capacity of lung cancer by regulating PEX3 and PHACTR2‑AS1 expression instead of PHACTR2. Oncology Reports. 2022;47(3):1–10.

49. Guimaraes-Young A, Feddersen CR, Dupuy AJ. Sleeping Beauty mouse models of cancer: microenvironmental influences on cancer genetics. Frontiers in oncology. 2019;9:611.

50. Cui Z, Mo J, Wang L, Wang R, Cheng F, Wang L, et al. Integrated bioinformatics analysis of serine racemase as an independent prognostic biomarker in endometrial cancer. Frontiers in Genetics. 2022;13:906291.

51. Hayes NV, Blackburn E, Boyle MM, Russell GA, Frost TM, Morgan BJ, et al. Expression of neuregulin 4 splice variants in normal human tissues and prostate cancer and their effects on cell motility. Endocrine-related cancer. 2011;18(1):39.

52. Halberg N, Sengelaub CA, Navrazhina K, Molina H, Uryu K, Tavazoie SF. PITPNC1 recruits RAB1B to the Golgi network to drive malignant secretion. Cancer cell. 2016;29(3):339–53.

53. Aydin B, Arga KY. Co-expression network analysis elucidated a core module in association with prognosis of non-functioning non-invasive human pituitary adenoma. Frontiers in endocrinology. 2019;10:458214.

54. Ioannidis NM, Wang W, Furlotte NA, Hinds DA, Bustamante CD, Jorgenson E, et al. Gene expression imputation identifies candidate genes and susceptibility loci associated with cutaneous squamous cell carcinoma. Nature communications. 2018;9(1):4264.

55. Xu S, Zheng Z, Pathak JL, Cheng H, Zhou Z, Chen Y, et al. The emerging role of the serine incorporator protein family in regulating viral infection. Frontiers in Cell and Developmental Biology. 2022;10:856468.

56. Majumdar S, Gong EM, Di Vizio D, Dreyfuss J, DeGraff DJ, Hager MH, et al. Loss of Sh3gl2/endophilin A1 is a common event in urothelial carcinoma that promotes malignant behavior. Neoplasia. 2013;15(7):749–IN16.

57. Hong Y, Tian X, Wang M, Chen C, Sun A. Bioinformatics-based identification of SPNS3 (Spinster homolog 3) as a prognostic biomarker of apoptosis resistance in acute myeloid leukemia. Bioengineered. 2021;12(1):7837–48.

58. GONG H, ZHOU Z, LU T, XU K, LU W. Expression of long-chain non-coding RNA CTA-796E4. 4 in bladder cancer and its effect on cell proliferation and invasion. Chinese Journal of Postgraduates of Medicine. 2020:1074–8.

59. Feng Y, Singleton D, Guo C, Gardner A, Pakala S, Kumar R, et al. DNA homologous recombination factor SFR1 physically and functionally interacts with estrogen receptor alpha. PLoS One. 2013;8(7):e68075.

60. Li X, Kim W, Arif M, Gao C, Hober A, Kotol D, et al. Discovery of functional alternatively spliced PKM transcripts in human cancers. Cancers. 2021;13(2):348.

61. Lim LJ, Ling LH, Neo YP, Chung AY, Goh BK, Chow PK, et al. Highly deregulated lncRNA LOC is associated with overall worse prognosis in Hepatocellular Carcinoma patients. Journal of Cancer. 2021;12(11):3098.

62. Zhang L, Liu M, Zhang Z, Chen D, Chen G, Liu M. Machine learning based identification of hub genes in renal clear cell carcinoma using multi-omics data. Methods. 2022;207:110–7.

63. van Heesch S, Witte F, Schneider-Lunitz V, Schulz JF, Adami E, Faber AB, et al. The translational landscape of the human heart. Cell. 2019;178(1):242–60. e29.

64. Wei W, Liu C, Wang M, Jiang W, Wang C, Zhang S. Prognostic signature and tumor immune landscape of N7-methylguanosine-related lncRNAs in hepatocellular carcinoma. Frontiers in Genetics. 2022;13:906496.

65. Zhu Z, Rong Z, Luo Z, Yu Z, Zhang J, Qiu Z, et al. Circular RNA circNHSL1 promotes gastric cancer progression through the miR-1306-3p/SIX1/vimentin axis. Molecular cancer. 2019;18:1–20.

66. Chen D, Li P, Guo W, Ye F, Wu J, Wei D, et al. Molecular evolution of candidate sour taste receptor gene PKD1L3 in mammals. Genome. 2011;54(11):890–7.

67. Sher AA, VanAllen ME, Ahmed H, Whitehead-Tillery C, Rafique S, Bell JA, et al. Conjugative RP4 Plasmid-Mediated Transfer of Antibiotic Resistance Genes to Commensal and Multidrug-Resistant Enteric Bacteria In Vitro. Microorganisms. 2023;11(1):193.

68. Shen W, Luo P, Sun Y, Zhang W, Zhou N, Zhan H, et al. NRBF2 regulates the chemoresistance of small cell lung cancer by interacting with the P62 protein in the autophagy process. Iscience. 2022;25(6).

69. Du Z-P, Wu B-L, Wu X, Lin X-H, Qiu X-Y, Zhan X-F, et al. A systematic analysis of human lipocalin family and its expression in esophageal carcinoma. Scientific reports. 2015;5(1):12010.
