# Supplementary material for: PICDGI: A framework for predicting cancer driver genes through dynamic gene-gene interaction modeling of single-cell data
Source: PLoS Comput Biol. 2026 Apr 27;22(4):e1014143. doi: 10.1371/journal.pcbi.1014143 (PMC13119913; doi:10.1371/journal.pcbi.1014143)
Supplement: S10 Text — (DOCX) [file pcbi.1014143.s030.docx]

# **S10 Text. Pathway Enrichment Analysis of PICDGI-Predicted Driver Genes Across Patients**

To complement the expression- and activity-based validation performed with the Kim et al[1]. dataset, we conducted additional pathway enrichment analyses to evaluate whether the PICDGI-prioritized driver genes and their inferred interaction partners converge on biologically coherent pathways relevant to LUAD progression and metastasis. For each patient, we performed over-representation analysis (ORA) using both Hallmark and Gene Ontology Biological Process (GO-BP) reference sets. These analyses reveal that PICDGI-predicted drivers map to pathways strongly implicated in cancer progression, metabolic rewiring, stress adaptation, and microenvironmental remodeling, thereby supporting their functional relevance in the metastatic context profiled by Kim et al[1].

## **Pathway enrichment for Patient 1 drivers and interaction partners**

## For Patient 1, ORA of the union of PICDGI-prioritized drivers and their modulatory partners identified a significant enrichment for **Hallmark: Cholesterol Homeostasis** (adjusted p ≈ 1.6×10⁻²) (S1 Fig. A). This enrichment is consistent with lipid-metabolic rewiring observed in LUAD, where alterations in cholesterol synthesis and membrane dynamics support proliferative demands and facilitate adaptation during tumor progression. Several top-ranked genes recovered by PICDGI fall within or regulate this metabolic axis, reinforcing the biological relevance of the inferred driver set.

GO-BP analysis further highlighted pathways central to malignant evolution, including **cell-cycle checkpoint regulation, negative regulation of cell-cycle phase transition**, **microtubule-based process regulation**, and **centrosome localization** (S1 Fig. B). These processes are tightly linked to mitotic fidelity and genome stability, hallmarks of LUAD progression and metastatic competence. Additional enriched processes included **macroautophagy/autophagic cell death** and **responses to oxidative stress**, programs that support tumor cell survival during nutrient stress and therapeutic exposure. Enrichment in **pseudopodium assembly** and **myofibroblast remodeling** implicates cytoskeletal reorganization and stromal interaction, suggesting that PICDGI does not merely identify co-expressed genes but instead recovers regulatory modules that condition epithelial tumor evolution within the microenvironment.

Taken together, these data show that PICDGI-predicted drivers for Patient 1 are embedded in metabolic, mitotic, stress-response, and cytoskeletal programs known to escalate in metastatic LUAD.

**
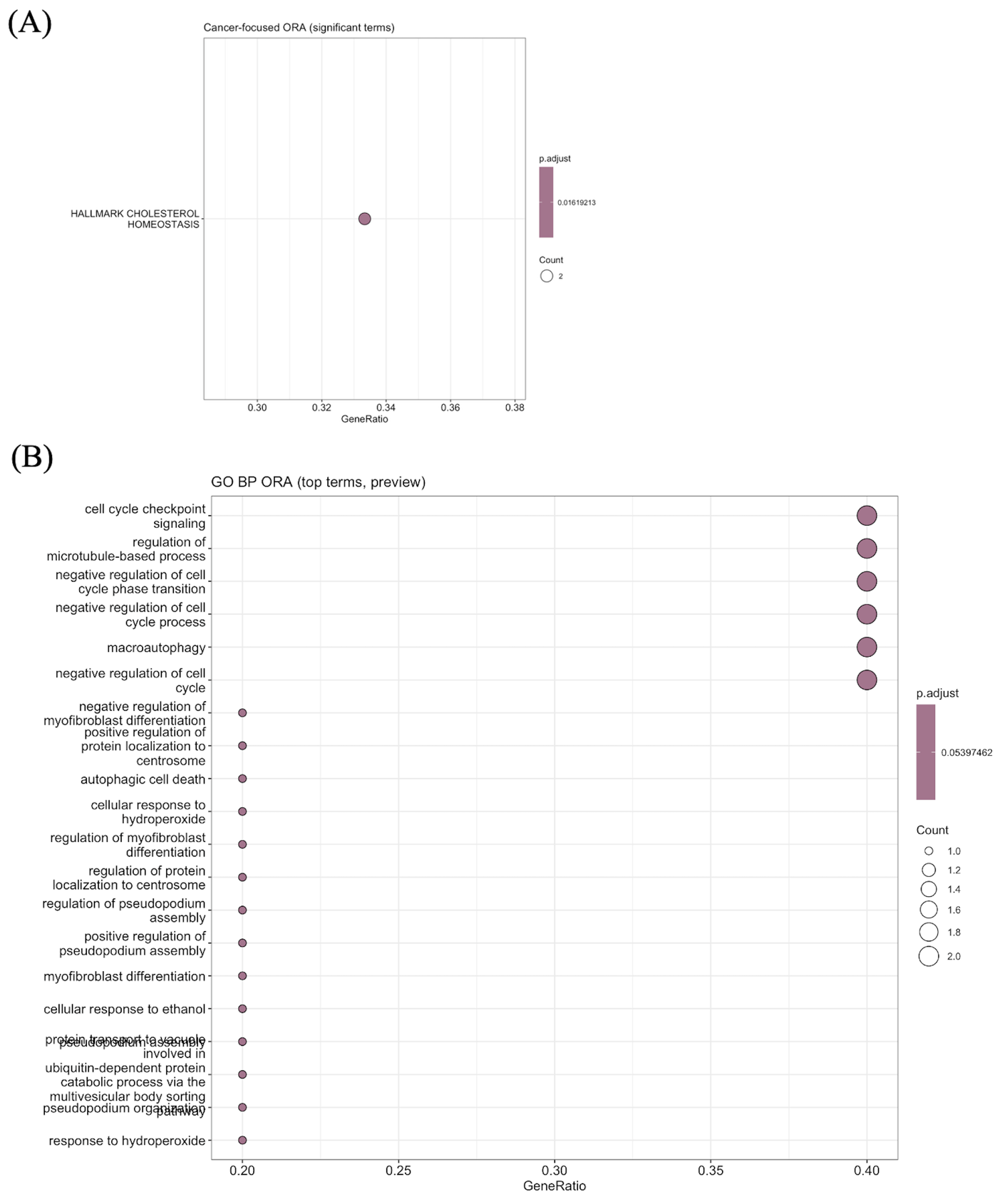
**

**S1 Fig. Pathway enrichment for patient P1 driver and modulator genes.** **A.** Cancer-focused ORA highlighting **Hallmark: Cholesterol Homeostasis** as significantly enriched, consistent with lipid-metabolic rewiring among prioritized genes. **B.** GO Biological Process ORA emphasizing malignancy-linked programs, including **cell-cycle checkpoint signaling, negative regulation of cell-cycle phase transition, regulation of microtubule-based processes/centrosome localization, macroautophagy/autophagic cell death, responses to hydroperoxide**, and **pseudopodium/myofibroblast programs**. Dot size = hit count; color = adjusted p; x-axis = GeneRatio.

## **Pathway enrichment for Patient 2 drivers and interaction partners**

## In Patient 2, ORA revealed a strong enrichment for cell-cycle and proliferative pathways, including **REACTOME: Cell-Cycle Checkpoints**, **Hallmark: G2M Checkpoint**, and **Hallmark: Mitotic Spindle** (S2 Fig. A). These pathways are canonical regulators of LUAD growth and metastatic fitness, aligning with increased cell-cycle activity observed in metastatic samples from Kim et al. Additional enriched signatures included perturbations of the **RB/P107 axis** and multiple **Polycomb-associated (PRC1/PRC2)** epigenetic programs, consistent with dedifferentiation and chromatin remodeling in advanced LUAD.


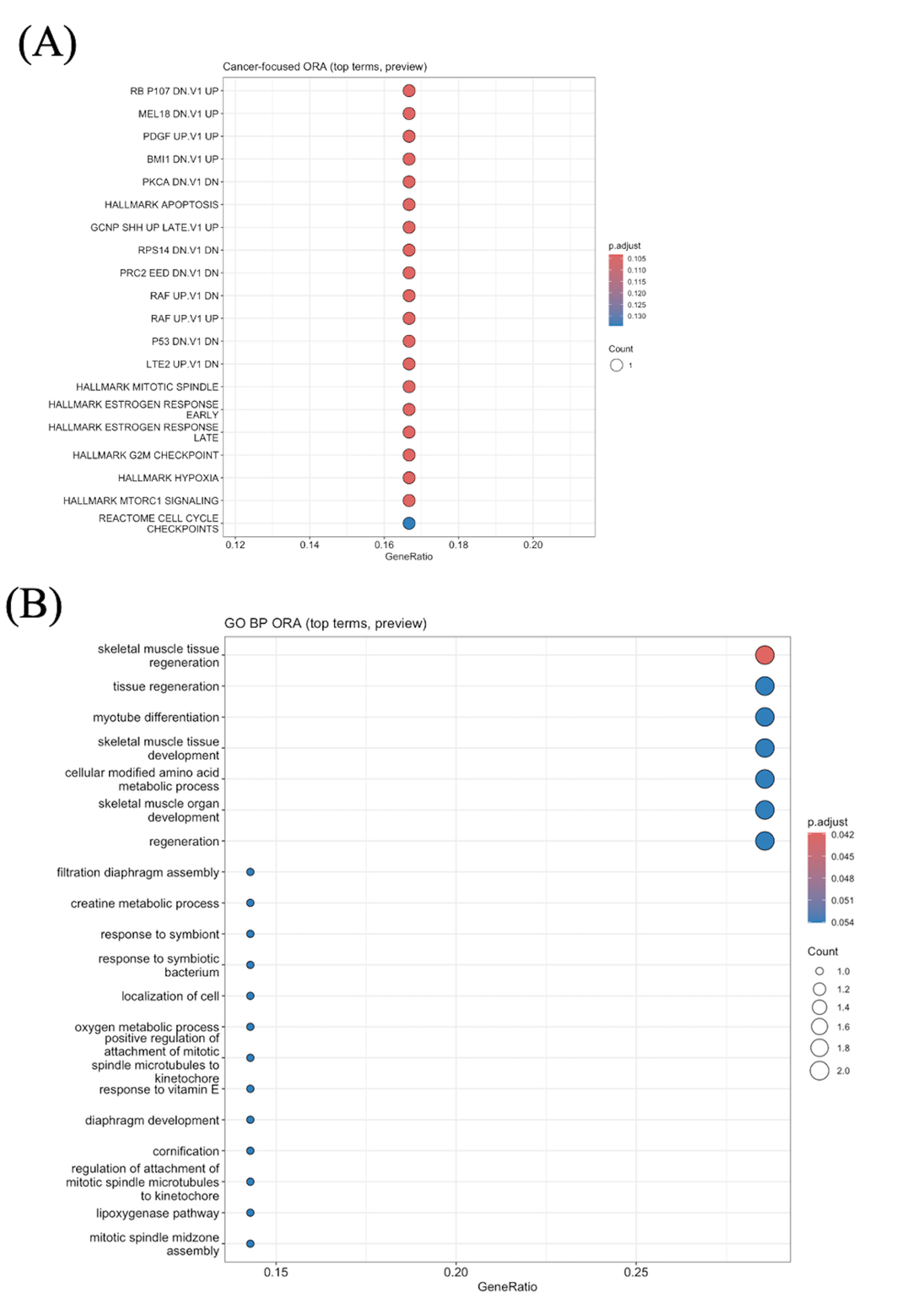


**S2 Fig. Pathway enrichment for patient P2 driver and modulator genes. A. Cancer-focused ORA showing coordinated activation of cell-cycle pathways (Reactome: Cell-Cycle Checkpoints, Hallmark: G2M Checkpoint, Mitotic Spindle) with RB/P107 down, Polycomb (BMI1/MEL18/PRC2) perturbation, PDGF/RAF/PKC signaling, mTORC1, Hypoxia, and Apoptosis. B. GO Biological Process ORA reinforcing mitotic control (spindle-kinetochore attachment, spindle midzone assembly), metabolic/redox remodeling (oxygen/creatine metabolism, lipoxygenase pathway), and regeneration/cell-localization terms. Dot size = hit count; color = adjusted p; x-axis = GeneRatio.**

We also observed enrichment in **PDGF**, **RAF**, **PKC**, and **mTORC1** signaling, together with **Hypoxia** and **Apoptosis** signatures, all pathways that are well described in LUAD progression and therapy resistance. These findings indicate that PICDGI-identified drivers for Patient 2 converge on proliferative, chromatin-regulatory, and growth-factor pathways that are central to metastatic-stage transcriptional reprogramming.

GO-BP enrichment reinforced these themes, highlighting **microtubule-kinetochore attachment**, **spindle midzone assembly**, and other mitotic processes directly tied to drivers recovered by PICDGI (S2 Fig. B). Additional enrichment in **oxygen metabolic processes**, **lipoxygenase activity**, and **creatine metabolism** indicates redox and metabolic adaptation consistent with increased metabolic stress in metastatic lesions. Terms involving **tissue regeneration**, **myogenic differentiation**, and **cell localization/motility** suggest stromal and extracellular matrix remodeling, in agreement with interaction patterns captured by PICDGI’s gene-gene influence modeling.

Overall, the Patient 2 driver set demonstrates convergent enrichment in proliferative, epigenetic, metabolic, and microenvironmental pathways, strongly supporting the biological plausibility of PICDGI predictions in metastatic LUAD.

## **Pathway enrichment for Patient 3 drivers and interaction partners**

## For Patient 3, cancer-focused ORA identified a striking enrichment for **autophagy-related pathways**, with three KEGG/MEDICUS reference sets corresponding to **autophagy vesicle nucleation, elongation, and maturation** significantly enriched (S3 Fig. A; FDR ≈ 0.014-0.015). These pathways involve the **PI3KC3 (class III PI3K) complexes I and II** and **mTORC1**, which together form the canonical regulatory triad governing autophagosome biogenesis and nutrient sensing. Autophagy regulation via PI3KC3 and mTORC1 is central to cancer cell survival under stress and is frequently upregulated in metastatic LUAD. The enrichment observed here indicates that PICDGI correctly identifies driver genes that converge on this survival axis.

**
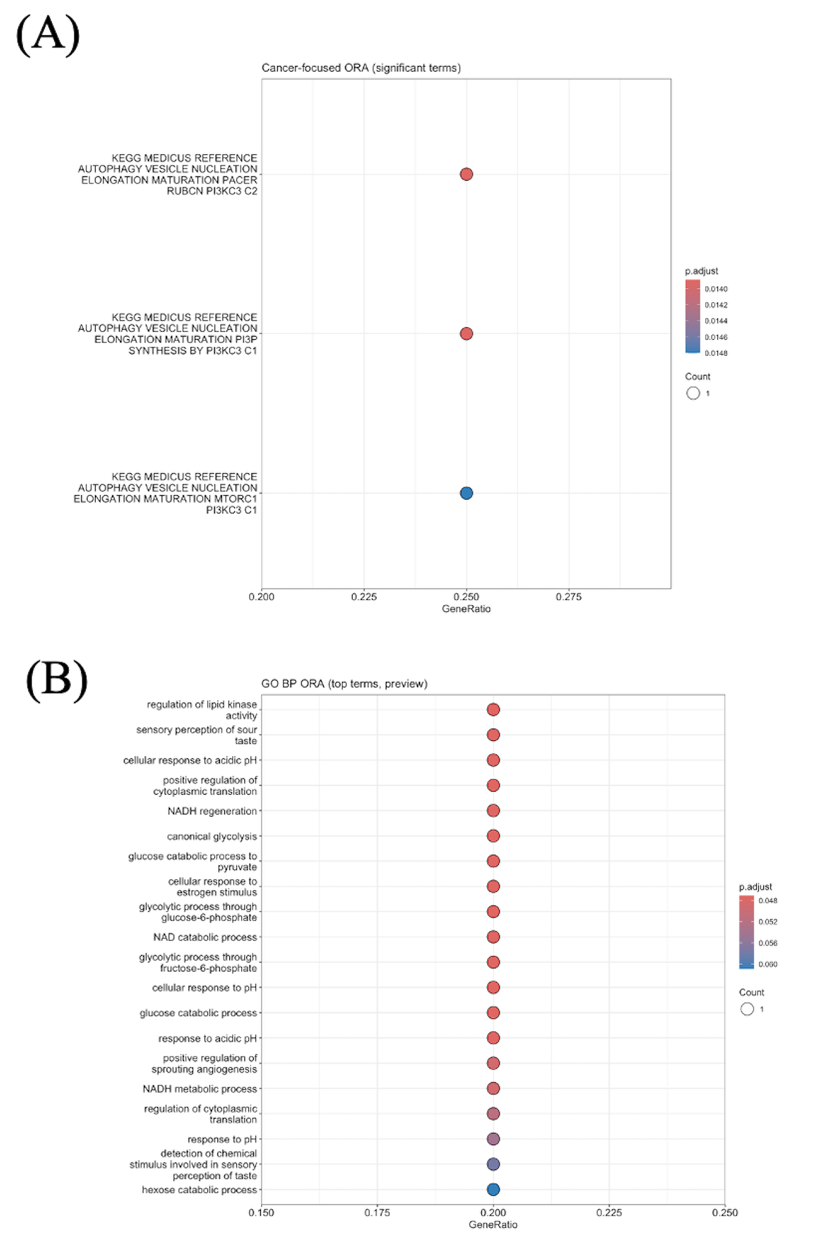
**

**S3 Fig. Pathway enrichment for patient P3 driver and modulator genes.** **A.** Cancer-focused ORA identifies a coherent **autophagy** axis, KEGG MEDICUS reference sets for **autophagy vesicle nucleation/elongation/maturation** involving the **PI3KC3 (class III PI3K) complex I/II** and **mTORC1** are significantly enriched. **B.** GO Biological Process ORA highlights **regulation of lipid kinase activity, regulation of cytoplasmic translation**, **NADH regeneration, canonical glycolysis** and related **glucose catabolic processes**, plus **cellular response to acidic pH** and **sprouting angiogenesis**, indicating coupled PI3K-mTOR/autophagy and metabolic stress-adaptation programs. Dot size denotes hit count; color denotes adjusted p-value; the x-axis shows GeneRatio.

GO-BP enrichment extended these findings by highlighting **regulation of lipid kinase activity**, **cytoplasmic translation**, **NADH regeneration**, and multiple **glycolytic processes**, reflecting the metabolic rewiring characteristic of aggressive LUAD lesions (S3 Fig. B). Terms related to **cellular response to acidic pH** and **sprouting angiogenesis** further point to adaptation to the hypoxic and lactate-rich metastatic microenvironment. Several pathways involving **cell migration and localization** were also enriched, suggesting that PICDGI captures gene modules influencing invasion and metastatic dissemination.

Collectively, the Patient 3 results show that PICDGI-prioritized drivers are strongly associated with autophagy regulation, bioenergetic remodeling, and cytoskeletal, microenvironmental adaptation, programs fundamental to metastatic survival and progression in LUAD.

## **Summary**

## Across all three patients, PICDGI-predicted driver genes and their modulatory partners consistently map to pathways that are **biologically central to LUAD progression**, including (1) metabolic rewiring (cholesterol synthesis, glycolysis, redox balance), (2) cell-cycle and mitotic control, (3) autophagy and nutrient-stress responses, (4) hypoxia, apoptosis, and mTOR signaling, and (5) cytoskeletal remodeling and microenvironmental interaction. These cross-patient enrichment patterns provide **functional validation** of PICDGI’s driver predictions within the Kim et al. dataset, demonstrating that the identified genes participate in pathways that intensify during metastatic evolution. This supplementary analysis therefore strengthens the biological grounding of PICDGI and supports its utility for identifying metastasis-associated cancer driver genes.

**References**

1. Kim N, Kim HK, Lee K, Hong Y, Cho JH, Choi JW, et al. Single-cell RNA sequencing demonstrates the molecular and cellular reprogramming of metastatic lung adenocarcinoma. Nature communications. 2020;11(1):2285.
