# Supplementary material for: PICDGI: A framework for predicting cancer driver genes through dynamic gene-gene interaction modeling of single-cell data
Source: PLoS Comput Biol. 2026 Apr 27;22(4):e1014143. doi: 10.1371/journal.pcbi.1014143 (PMC13119913; doi:10.1371/journal.pcbi.1014143)
Supplement: S11 Text — (DOCX) [file pcbi.1014143.s034.docx]

**S11 Text. External Validation of PICDGI Using an Independent Pediatric AML scRNA-seq Cohort and Cross-Dataset Driver Inference Analysis**

We selected the independent single-cell RNA-seq dataset generated by Mumme et al. (GEO: GSE235923) [1, 2] , which profiles pediatric acute myeloid leukemia (AML) from bone-marrow aspirates collected at three clinically meaningful stages: Diagnosis (Dx), End-of-Induction chemotherapy (EOI), and Relapse. The cohort includes 31 samples from 19 pediatric AML patients at diagnosis, 10 patients at end of induction chemotherapy, and 2 patients at relapse, capturing both temporal variation and inter-patient diversity. The study was designed to contrast relapse-associated versus continuous complete remission (CCR)-associated cases across these time points, using scRNA-seq to resolve malignant blasts and tumor-microenvironment populations.

In addition to providing raw and processed single-cell RNA-seq data across **Dx**, **EOI**, and **Relapse**, the study by **Mumme** et al. reports several **microenvironmental and leukemic programs directly relevant to causal and driver inference**. At diagnosis, relapse-associated samples are enriched for **exhausted T-cell states**, whereas CCR samples show increased representation of **inflammatory (M1-like) macrophages**, highlighting divergent immune contexts associated with disease outcome[2]. Following induction chemotherapy, residual leukemic blasts up-regulate transcriptional programs linked to **fatty-acid oxidation, cellular growth, and stemness**, consistent with metabolic reprogramming and therapy resistance observed in pediatric AML[2, 3]. Importantly, the authors also define a **7-gene AML blast signature** (CLEC11A, PRAME, AZU1, NREP, ARMH1, C1QBP, and TRH) that robustly distinguishes malignant blasts from non-malignant hematopoietic cells and is validated across independent pediatric AML cohorts[2]. This curated signature provides an objective biological benchmark for evaluating external consistency of driver-ranking approaches, enabling assessment of whether prioritized genes and programs align with experimentally supported leukemic blast biology.

This dataset is particularly suitable for evaluating the reproducibility of *PICDGI* on an independent scRNA-seq dataset for three main reasons. **First**, it is fully independent of our LUAD discovery analysis and spans **three longitudinal stages** (Dx → EOI → Relapse), enabling an out-of-sample test of our trajectory-based inference rather than a re-analysis of training data. **Second**, because the cohort resolves both **malignant blasts and immune/stromal compartments**, it allows us to assess whether PICDGI recovers driver dynamics and inferred regulatory interactions that are consistent with known relapse-associated biology (e.g., T-cell exhaustion, macrophage polarization) without any re-tuning. **Third**, the study’s curated signatures and stage labels provide objective anchors for **frozen-parameter** validation (e.g., rank overlap, AUC, and network-edge enrichment versus discovery results), thereby strengthening confidence that our model framework can be applied to **different disease contexts and platforms**.

**Processing and Annotation of Three Staged Single-Cell RNA-seq Datasets Using Seurat**

To mitigate imbalances in cell-type representation and preserve cell-type specific structure, we pooled and processed the three staging scRNA-seq datasets using a unified Seurat workflow to ensure comparability across samples. After loading raw count matrices, we performed stringent quality control by removing droplets with fewer than 200 detected genes, more than 6,000 genes, or more than 15-20% mitochondrial UMIs, and we excluded erythrocyte-only droplets with very high HBA/HBB expression. We normalized each dataset with SCTransform (v2; glmGamPoi), regressing out percent mitochondrial reads and cell-cycle scores. We then computed PCA on the top 3,000 highly variable genes, constructed a k-nearest neighbor graph on the first 30 PCs, and identified communities with Louvain clustering (resolution 0.4-0.8, tuned per dataset to separate major lineages without over-fragmentation). UMAP on the same PCs provided the low-dimensional representations shown in the S1 Fig.

To assign biological identities to single-cell clusters, we integrated **data-driven differential expression analysis** with **curated lineage- and leukemia-specific marker panels**. Differential markers were identified using FindAllMarkers (Wilcoxon rank-sum test; min.pct = 0.25, log2FC > 0.25, FDR-adjusted P < 0.05) and interpreted the clusters in the context of established hematopoietic and AML biology. Malignant myeloid and leukemic populations were defined using a comprehensive **AML blast gene set**, encompassing granule and protease genes (MPO, ELANE, CTSD, CTSG, AZU1, PRTN3, LYZ), inflammatory and innate immune markers (S100A8/A9, FCGR3B, MNDA, TYMP, LGALS3, CXCL8, LST1), transcriptional and developmental regulators central to AML pathogenesis (RUNX1, HOXA9, SOX4, CEBPE), stem and progenitor markers (CD34, CD117/KIT, CD33), and genes associated with leukemic proliferation and survival (BCL2, UBE2C, CD44, PRAME)[3-5].

Non-malignant immune populations were annotated using established lineage markers. **Immature B cells** were identified by TCL1A, VPREB3, FAM129C, and FCER2, distinguishing them from mature B cells and plasmablasts. T-cell populations were defined by CD3D/E and TRAC, with naïve or central-memory states marked by CCR7, TCF7, and LEF1, and cytotoxic states by GZMB, PRF1, and NKG7. NK cells were identified using KLRD1, GNLY, FGFBP2, and NKG7. Additional lineages were annotated using canonical markers, including endothelial cells (PECAM1, VWF, CLDN5), dendritic cells (CD1C, FCER1A, and CLEC9A), eosinophils (PRG2, RNASE2/3), fibroblasts (COL1A1, DCN, PDGFRA), and cycling states (MKI67, TOP2A, PCNA, HIST1H1B). Where appropriate, annotations were validated by Azimuth label transfer against PBMC references and by gene-set enrichment analysis of hallmark pathways.

In the first dataset (S1 Fig A, **Diagnosis (Stage 1)**), we observed a leukemic ecosystem dominated by **myeloid populations**, with relatively limited lymphoid and stromal infiltration. The transcriptional landscape was characterized by a prominent **AML blast-enriched core**, encompassing **LSC-like**, **myeloblast**, **myelomonocytic**, and **granulocytic** populations. These clusters expressed canonical AML-associated genes, including granule and protease markers (MPO, ELANE, CTSG, CTSD, AZU1, PRTN3, and LYZ), inflammatory markers (S100A8 and S100A9), and progenitor or stem-associated regulators (CD34, CD117/KIT, CD33, RUNX1, and HOXA9)[3, 4]. In addition, **cycling myeloid and cycling monocytic populations** were identified by the coordinated expression of cell-cycle genes (MKI67, TOP2A, PCNA, and UBE2C) together with AML blast markers, indicating active leukemic proliferation at diagnosis [1,2]. Collectively, these features demonstrate that malignant transcriptional programs are already distributed across multiple stages of myeloid differentiation at diagnosis, consistent with early involvement of leukemic stem and progenitor compartments. In contrast, non-malignant immune lineages contributed only minimally to the overall cellular landscape at this stage, highlighting the dominance of leukemic myeloid states at diagnosis in pediatric AML[2, 6].

The second dataset (S1 Fig. A, **End-of-Induction (Stage 2)**) exhibited the greatest diversity of leukemic and microenvironmental cell states. Although cytotoxic therapy reduced overall cellularity in several compartments, **malignant myeloid populations persisted**, including **early myeloid progenitors**, **cycling myeloid and monocytic populations**, **CD34⁺ granulocytic cells**, and **CXCL8⁺ inflammatory monocytic states**. These populations retained strong expression of canonical AML-associated genes (MPO, ELANE, AZU1, CTSG, S100A8/A9, CXCL8, CD44, and BCL2), consistent with **therapy-resistant leukemic cells** that have been shown to survive induction chemotherapy and seed relapse in pediatric AML[3, 4]. In parallel, we observed expansion of immune and stromal compartments, including **IFN-responsive and IFN-stimulated myeloid cells** marked by IFITM2, IFI30, ISG15, and DUSP6, as well as inflammatory monocytic states (LYZ, LST1, LGALS3, MNDA), reflecting treatment-associated immune activation and inflammatory remodeling of the bone marrow microenvironment[7, 8]. Non-malignant lymphoid populations including naïve and cytotoxic T cells, NK cells, and immature B cells were also readily resolved and remained largely distinct from malignant compartments. The **coexistence of persistent AML-enriched myeloid populations with predominantly non-malignant immune lineages** at this stage enabled robust estimation of cancer cell fractions across lineages (stacked bar plots, Supplementary S1B Fig) and provided critical context for downstream PICDGI-based driver inference.

The third dataset (S1 Fig. A, **Relapse (Stage 3)**) was again dominated by **malignant myeloid populations**, consistent with clonal persistence and re-expansion following therapy. We observed abundant **LSC-like**, **myeloblast**, **granulocytic**, **cycling granulocytic**, **monocytic**, and **inflammatory monocytic** states, all of which exhibited **high malignant cell fractions** after cell fraction analysis. These populations expressed strong **AML blast and inflammatory gene signatures**, including myeloid granule and protease genes (MPO, ELANE, CTSG, AZU1), inflammatory and innate immune markers (S100A8/A9, FCGR3B, CXCL8), and monocyte-associated markers (CD163), together with elevated expression of cell-cycle genes in cycling subsets. This transcriptional profile is consistent with prior studies demonstrating that pediatric AML relapse is driven by therapy-resistant leukemic stem and progenitor populations that retain proliferative capacity and inflammatory programs, rather than by expansion of non-malignant immune compartments[2-4].

Together, this standardized annotation strategy grounded in AML blast gene expression, lineage-defining markers, and malignant fraction dynamics provides a reproducible and quantitative foundation for downstream PICDGI analyses. By explicitly linking cell-state identity to cancer cell fraction trajectories across disease stages, this framework enables robust inference of leukemia cell-of-origin and principled prioritization of pediatric AML driver genes

**
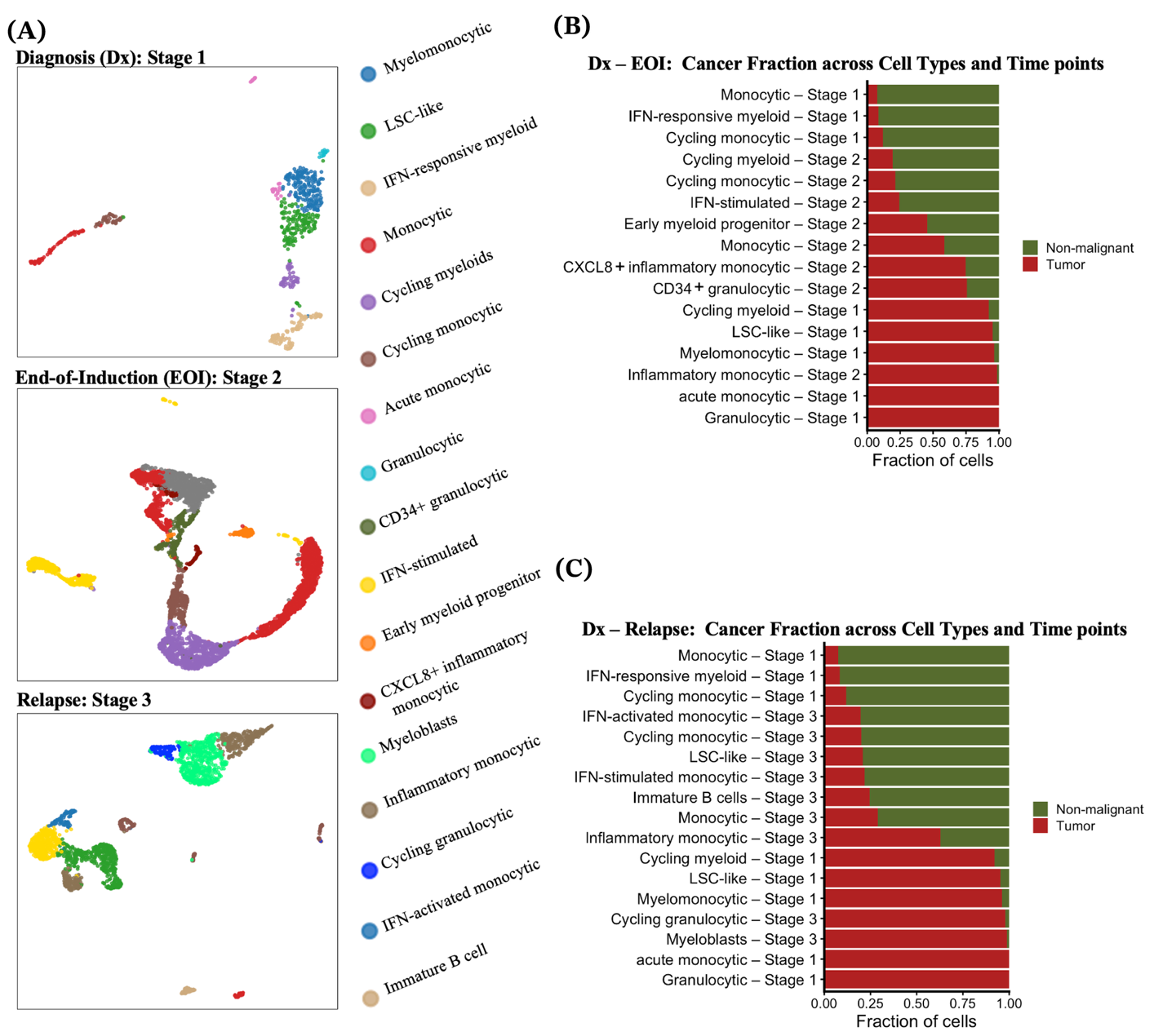
**

**S1 Fig. Cellular composition and cancer cell fraction dynamics across disease stages**. **A.** UMAP embeddings of single cells colored by annotated myeloid and lymphoid cell states at diagnosis (Dx), early induction/consolidation (EOI), and relapse, illustrating stage-specific shifts in cellular composition. Right panels display horizontal stacked bar plots summarizing the fraction of tumor (red) versus non-malignant (green) cells within each annotated cell type and stage. Cell types are ordered by increasing tumor fraction. **B.** Dx-EOI; several cycling, progenitor, and inflammatory myeloid populations show partial malignant involvement, whereas differentiated monocytic and granulocytic populations are predominantly malignant. **C.** Dx-Relapse; malignant fractions increase across multiple compartments after therapy, including LSC-like, cycling myeloid/monocytic, and granulocytic populations, highlighting clonal expansion and disease evolution over time.

**Myeloid lineage leukemic cells are the most likely cancer-progenitor population across stages**

Using PICDGI (cell-level malignancy scoring followed by aggregation by lineage and stage), we first assigned each cell a malignancy probability by integrating AML blast-associated gene programs with local neighborhood smoothing to distinguish leukemic from non-malignant hematopoietic cells. We then summarized these per-cell scores within each annotated cell type at each clinical stage: Diagnosis (Stage 1), End-of-Induction (Stage 2), and Relapse (Stage 3) to estimate the fraction of malignant cells (red) versus non-malignant cells (green) per lineage (**Dx-EOI** and **Dx-Relapse)**. The resulting small-multiple plots visualize these fractions across major myeloid, lymphoid, and progenitor compartments and highlight the persistence and dominance of malignant signal within myeloid populations across Dx-EOI and Dx-Relapse comparisons (S1 Figs. B-C).

From this analysis, the **myeloid leukemic compartment** emerges as the dominant malignant lineage in the Mumme/Bhasin pediatric AML cohort, with particularly strong and persistent malignant signals in stem-like, cycling, and differentiated myeloid populations. Three observations support this conclusion. **First**, at diagnosis, LSC-like, cycling myeloid/monocytic, and granulocytic populations already exhibit high malignant fractions relative to other annotated cell states, consistent with early leukemic involvement of the myeloid hierarchy in pediatric AML. **Second**, following induction therapy, malignant fractions persist within these same compartments, whereas non-myeloid and immune bystander populations remain largely non-malignant, indicating relative therapy resistance of leukemic progenitor and cycling myeloid cells. **Third**, at relapse, malignant fractions again dominate LSC-like, cycling myeloid, monocytic, and granulocytic populations, reflecting clonal persistence and re-expansion, a hallmark of pediatric AML relapse.

While isolated non-myeloid populations (e.g., IFN-stimulated or endothelial-like states) occasionally display malignant fractions at a single stage, these signals lack the **stage-to-stage continuity** required by PICDGI to infer a leukemia cell-of-origin. In contrast, the magnitude and reproducibility of malignant involvement within myeloid compartments across diagnosis, post-induction, and relapse satisfy all PICDGI criteria: **early presence, persistence after cytotoxic therapy, and enrichment at relapse** supporting the myeloid lineage, particularly LSC-like and cycling myeloid populations, as the leukemia-originating cellular compartment in this pediatric AML cohort.

Leveraging this **population-level cellular context**, we applied **PICDGI** to prioritize candidate cancer driver genes by integrating **malignant cell fraction dynamics** with **disease-transition aware gene ranking**. For each longitudinal comparison (**Dx-EOI** and **Dx-Relapse**), genes were ranked according to their **driver coefficient (DrCoef)**, and the top 20 candidates were visualized (S2 Figs. A-B), with previously established cancer drivers explicitly flagged. This analysis recovered multiple genes with well-documented relevance to **hematologic malignancies, including pediatric AML**, supporting the biological validity of the framework.

Most notably, ***PRDM1* (*BLIMP1*)**, a transcriptional regulator with established tumor-suppressive roles in hematologic cancers was highly ranked during the **Dx-Relapse transition**. *PRDM1* is recurrently altered in leukemias and lymphoid malignancies and plays a critical role in controlling differentiation, cell-cycle exit, and survival of malignant hematopoietic cells. Its prioritization by PICDGI during relapse is consistent with previous studies linking *PRDM1* dysregulation to leukemic persistence and disease progression, underscoring the ability of PICDGI to recover biologically meaningful driver signals in pediatric AML[2, 5, 9].

In addition, several **context-dependent oncogenic facilitators** known to support acute myeloid leukemia biology were consistently prioritized by PICDGI. ***STIP1***, a co-chaperone of the *HSP90/HSP70* complex, has been shown to promote leukemic cell survival and proteotoxic stress tolerance by stabilizing oncogenic signaling proteins, and its overexpression has been associated with poor prognosis in hematologic malignancies[10, 11]. ***GLO1***, a key enzyme in methylglyoxal detoxification, supports glycolytic flux and redox homeostasis, processes that are critical for the metabolic demands of rapidly proliferating AML blasts and have been linked to chemoresistance[12, 13]. **ISG15**, a ubiquitin-like modifier induced by type I interferon signaling, reflects inflammatory and stress-response programs frequently activated in pediatric AML and has been implicated in leukemic cell survival and immune evasion depending on cellular context[2, 8]. Finally, ***CD164***, a sialomucin adhesion receptor expressed on hematopoietic progenitors and AML blasts, has been associated with enhanced leukemic cell survival, bone marrow niche retention, and migratory capacity, highlighting its role in leukemia-microenvironment interactions[14].

Although these genes are not classical initiating driver mutations, their **recurrent enrichment among top-ranked candidates** and their well-established roles in stress adaptation, metabolism, immune signaling, and niche engagement underscore their functional importance in sustaining leukemic states and promoting therapy resistance.

Beyond these established AML-associated genes, PICDGI highlights a coherent group of **additional candidate genes** with biologically plausible roles in pediatric AML pathogenesis. These include genes involved in **protein homeostasis and proteasomal regulation**, such as ***PSMD6***, a component of the 26S proteasome that supports protein turnover and stress tolerance in rapidly proliferating malignant cells[15]. PICDGI also prioritizes genes involved in **RNA splicing and transcriptional control**, including ***SRRM1*** and ***WBP11***, consistent with extensive evidence that dysregulation of RNA processing and splicing programs contributes to leukemogenesis and disease progression in myeloid malignancies[16, 17].

In addition, PICDGI nominates genes linked to **metabolic and mitochondrial processes**, such as ***LAGE3***, ***MCUR1***, and ***ATP5MC3***, which are involved in mitochondrial translation, calcium uptake, and oxidative phosphorylation, respectively. These pathways are increasingly recognized as critical for leukemic stem cell survival, metabolic plasticity, and resistance to chemotherapy in AML[18, 19]. PICDGI further identifies genes associated with **immune modulation and cellular stress responses**, including ***TXNDC11***, implicated in redox and endoplasmic reticulum stress regulation, and ***SELPLG***, a leukocyte adhesion molecule mediating interactions between hematopoietic cells and the bone marrow microenvironment[20].

Although these genes are not currently classified as canonical AML driver genes, their **consistent upregulation across malignant myeloid populations and disease transitions** suggests that they may function as **context-dependent facilitators** of leukemic maintenance, therapy adaptation, or relapse-specific fitness rather than as initiating oncogenic events. Collectively, these observations indicate that PICDGI captures not only known drivers but also biologically coherent candidate genes that align with established mechanisms of pediatric AML progression and therapeutic resistance.

Importantly, the top-ranked genes form a relatively tight band of elevated DrCoef values, indicating a strong and coherent driver-associated signal rather than isolated transcriptional outliers. The enrichment of known pediatric AML-relevant drivers and facilitators near the top of the ranking, together with the functional convergence of newly nominated candidates, demonstrates that **PICDGI preferentially identifies genes linked to leukemic cell identity, survival, and progression**. Collectively, these results support PICDGI as a robust framework for prioritizing biologically meaningful cancer driver genes in pediatric AML, bridging single-cell cellular dynamics with gene-level driver discovery.


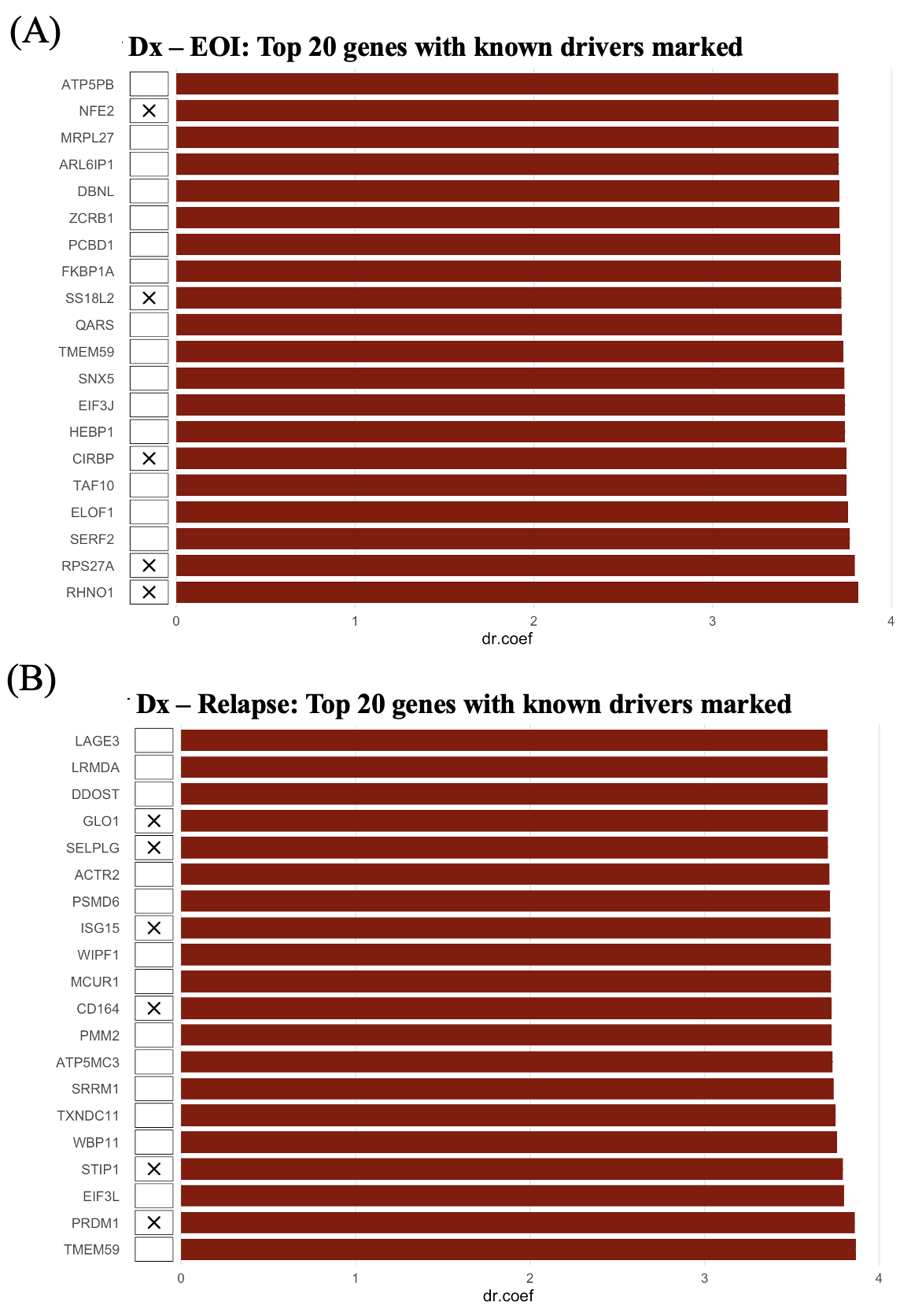


**S2 Fig. Differential gene ranking across disease transitions with known cancer drivers highlighted.** Horizontal bar plots show the top 20 genes ranked by driver-related coefficient (DrCoef) for the diagnosis: early induction/consolidation (A) and diagnosis-relapse (B) comparisons. Bars indicate the magnitude of the dr.coef for each gene, and genes previously reported as cancer drivers are marked with an “×”. In the Dx-EOI comparison, identified driver-associated genes include NFE2, a transcription factor recurrently implicated in myeloid malignancies through gain-of-function mutations that promote aberrant erythro-myeloid differentiation, as well as CIRBP, SS18L2, and RPS27A, which have been linked to cancer through roles in stress responses, chromatin regulation, or ribosomal dysregulation. In the Dx-Relapse comparison, known cancer-associated genes include PRDM1 (BLIMP1), a well-established tumor suppressor frequently inactivated in hematologic malignancies, particularly lymphoid cancers, and implicated in differentiation control. Additional marked genes such as GLO1, ISG15, CD164, SELPLG, and STIP1 are not canonical drivers but have documented roles in cancer cell survival, immune modulation, adhesion, metabolic stress tolerance, or therapy resistance, suggesting their contribution to malignant progression and relapse-associated adaptation rather than disease initiation. Together, these results indicate that while few classical driver genes dominate the top-ranks, relapse-associated transcriptional changes increasingly involve cancer-relevant pathways related to differentiation blockade, immune signaling, and cellular stress responses.

**References**

1. Mumme HL, Huang C, Ohlstrom D, Bakhtiari M, Raikar SS, DeRyckere D, et al. Identification of leukemia-enriched signature through the development of a comprehensive pediatric single-cell atlas. Nature Communications. 2025;16(1):4114.

2. Mumme H, Thomas BE, Bhasin SS, Krishnan U, Dwivedi B, Perumalla P, et al. Single-cell analysis reveals altered tumor microenvironments of relapse-and remission-associated pediatric acute myeloid leukemia. Nature Communications. 2023;14(1):6209.

3. Shlush LI, Mitchell A, Heisler L, Abelson S, Ng SW, Trotman-Grant A, et al. Tracing the origins of relapse in acute myeloid leukaemia to stem cells. Nature. 2017;547(7661):104–8.

4. van Galen P, Hovestadt V, Wadsworth II MH, Hughes TK, Griffin GK, Battaglia S, et al. Single-cell RNA-seq reveals AML hierarchies relevant to disease progression and immunity. Cell. 2019;176(6):1265–81. e24.

5. Zeng AG, Bansal S, Jin L, Mitchell A, Chen WC, Abbas HA, et al. A cellular hierarchy framework for understanding heterogeneity and predicting drug response in acute myeloid leukemia. Nature medicine. 2022;28(6):1212–23.

6. Papaemmanuil E, Gerstung M, Bullinger L, Gaidzik VI, Paschka P, Roberts ND, et al. Genomic classification and prognosis in acute myeloid leukemia. New England Journal of Medicine. 2016;374(23):2209–21.

7. Ivashkiv LB, Donlin LT. Regulation of type I interferon responses. Nature Reviews Immunology. 2014;14(1):36–49.

8. Desai SD. ISG15: A double edged sword in cancer. Oncoimmunology. 2015;4(12):e1052935.

9. Mandelbaum J, Bhagat G, Tang H, Mo T, Brahmachary M, Shen Q, et al. BLIMP1 is a tumor suppressor gene frequently disrupted in activated B cell-like diffuse large B cell lymphoma. Cancer cell. 2010;18(6):568–79.

10. Saini J, Sharma PK. Clinical, prognostic and therapeutic significance of heat shock proteins in cancer. Current drug targets. 2018;19(13):1478–90.

11. Guo X, Yan Z, Zhang G, Wang X, Pan Y, Huang M. STIP1 regulates proliferation and migration of lung adenocarcinoma through JAK2/STAT3 signaling pathway. Cancer Management and Research. 2019:10061–72.

12. Thornalley P. Glyoxalase I–structure, function and a critical role in the enzymatic defence against glycation. Biochemical Society Transactions. 2003;31(6):1343–8.

13. Antognelli C, Talesa VN. Glyoxalases in urological malignancies. International journal of molecular sciences. 2018;19(2):415.

14. Kulkarni R, Kale V. Physiological cues involved in the regulation of adhesion mechanisms in hematopoietic stem cell fate decision. Frontiers in Cell and Developmental Biology. 2020;8:611.

15. Cenci S, editor The proteasome in terminal plasma cell differentiation. Seminars in hematology; 2012: Elsevier.

16. Yoshida K, Ogawa S. Splicing factor mutations and cancer. Wiley Interdisciplinary Reviews: RNA. 2014;5(4):445–59.

17. Anande G, Deshpande NP, Mareschal S, Batcha AM, Hampton HR, Herold T, et al. RNA splicing alterations induce a cellular stress response associated with poor prognosis in acute myeloid leukemia. Clinical Cancer Research. 2020;26(14):3597–607.

18. Lagadinou ED, Sach A, Callahan K, Rossi RM, Neering SJ, Minhajuddin M, et al. BCL-2 inhibition targets oxidative phosphorylation and selectively eradicates quiescent human leukemia stem cells. Cell stem cell. 2013;12(3):329–41.

19. Jones CL, Inguva A, Jordan CT. Targeting energy metabolism in cancer stem cells: progress and challenges in leukemia and solid tumors. Cell stem cell. 2021;28(3):378–93.

20. Hetz C, Papa FR. The unfolded protein response and cell fate control. Molecular cell. 2018;69(2):169–81.
